# Multicenter Phase II Study of Sequential Radioembolization-Sorafenib Therapy for Inoperable Hepatocellular Carcinoma

**DOI:** 10.1371/journal.pone.0090909

**Published:** 2014-03-10

**Authors:** Pierce K. H. Chow, Donald Y. H. Poon, Maung-Win Khin, Harjit Singh, Ho-Seong Han, Anthony S. W. Goh, Su-Pin Choo, Hee-Kit Lai, Richard H. G. Lo, Kiang-Hiong Tay, Teong-Guan Lim, Mihir Gandhi, Say-Beng Tan, Khee-Chee Soo, for the Asia-Pacific Hepatocellular Carcinoma Trials Group

**Affiliations:** 1 National Cancer Centre, Singapore, Singapore; 2 Singapore General Hospital, Singapore, Singapore; 3 Duke-NUS Graduate Medical School, Singapore, Singapore; 4 Yangon Gastrointestinal and Liver Centre, Yangon, Myanmar; 5 Selayang Hospital, Selangor, Malaysia; 6 Seoul National University Bundang Hospital, Bundang, South Korea; 7 Singapore Clinical Research Institute, Singapore, Singapore; University of Bari Medical School, Italy

## Abstract

**Background:**

The safety and tolerability of sequential radioembolization-sorafenib therapy is unknown. An open-label, single arm, investigator-initiated Phase II study (NCT0071279) was conducted at four Asia-Pacific centers to evaluate the safety and efficacy of sequential radioembolization-sorafenib in patients with hepatocellular carcinoma (HCC) not amenable to curative therapies.

**Methods:**

Sorafenib (400 mg twice-daily) was initiated 14 days post-radioembolization with yttrium-90 (^90^Y) resin microspheres given as a single procedure. The primary endpoints were safety and tolerability and best overall response rate (ORR) using RECIST v1.0.Secondary endpoints included: disease control rate (complete [CR] plus partial responses [PR] and stable disease [SD]) and overall survival (OS).

**Results:**

Twenty-nine patients with Barcelona Clinic Liver Cancer (BCLC) stage B (38%) or C (62%) HCC received a median of 3.0 GBq (interquartile range, 1.0) ^90^Y-microspheres followed by sorafenib (median dose/day, 600.0 mg; median duration, 4.1 months). Twenty eight patients experienced ≥1 toxicity; 15 (52%) grade ≥3. Best ORR was 25%, including 2 (7%) CR and 5 (18%) PR, and 15 (54%) SD. Disease control was 100% and 65% in BCLC stage B and C, respectively. Two patients (7%) had sufficient response to enable radical therapy. Median survivals for BCLC stage B and C were 20.3 and 8.6 months, respectively.

**Conclusions:**

This study shows the potential efficacy and manageable toxicity of sequential radioembolization-sorafenib.

**Trial Registration:**

ClinicalTrials.gov NCT00712790.

## Introduction

Approximately 650,000 persons die each year from hepatocellular carcinoma (HCC), of whom at least two-thirds live in the Asia-Pacific region [1]. Consistent with the experience in most Western countries, ∼20% of patients within Asia-Pacific clinical practice are diagnosed at a sufficiently early stage to benefit from potentially curative therapies (resection, transplantation, ablation) [2]. The remainder suffers from locally advanced or systemic HCC and mortality from HCC continues to approximate its incidence [1].

Radioembolization with yttrium-90 (^90^Y) radiolabelled microspheres (also known as selective internal radiation therapy, SIRT) significantly regresses locoregional HCC, but does not address systemic disease [3], [4]. Conversely, while sorafenib has been shown to be an effective systemic therapy and confers a survival advantage, tumor regression is minimal and an objective tumor response is observed in <5% of patients by Response Evaluation Criteria In Solid Tumors (RECIST) [5], [6]. The addition of a proven systemic therapy (sorafenib) to therapy that reliably regresses locoregional tumor (radioembolization) could thereby confer an additional survival benefit.

The theoretical benefit of combined radiotherapy and sorafenib is supported by several preclinical studies. Radiation exposure is thought to induce the compensatory activations of multiple intracellular signaling pathway mediators, such as PI3K, MAPK, JNK and NF-kB [7] as well as the up-regulation of vascular endothelial growth factor (VEGF) [8]. It has been hypothesized that sorafenib-mediated inhibition of the Raf/MAPK and VEGF receptor pathways might enhance the efficacy of radiation [9]. Although the data are limited, *in-vivo* studies have shown that sorafenib alters the radiation response in a schedule-dependent manner [10]. Sorafenib administered after radiation therapy is associated with a greater delay in tumor growth than sorafenib pre-treatment [10], [11]. The efficacy and safety of three-dimensional conformal radiation therapy in augmenting the local response to sorafenib has been reported [9]. However, these studies are limited by the total irradiation dose that can be safely tolerated in patients with a higher tumor burden given the sensitivity of the normal parenchyma to radiation [12], [13].

^90^Y-microspheres are well tolerated by patients with non-cirrhotic livers and in those with cirrhotic livers without ascites and in whom total bilirubin is <2.0 mg/dL [14]. Radioembolization may also be used in HCC patients with portal vein thrombosis, a situation that precludes trans-arterial chemoembolization (TACE). Radioembolization has thus developed as an alternative to TACE, as an option in patients who are poor candidates for TACE or who have progressive disease after having received prior TACE [3], [4], [14].

The results of the Phase I study of this combination therapy have been previously reported [15]. We report here the efficacy of radioembolization followed by sorafenib in unresectable HCC in the Phase II study.

## Methods

### Study design

This was an open-label, single arm, investigator-initiated Phase II multicenter study conducted by the Asia-Pacific Hepatocellular Carcinoma Trials Group. Patients were recruited from seven tertiary medical centers in four Asia-Pacific countries (Malaysia; Myanmar; Singapore; South Korea) with radioembolization performed (as a single procedure) at one center (Singapore). The study was registered with the clinical trial registry of the Health Science Authority of Singapore (HSA) in June 2008, and ClinicalTrials.gov (Identifier: NCT00712790) in July 2008.

The previously reported Phase I found a greater incidence of grade 3 or 4 adverse events (mainly hand-foot syndrome) when sorafenib was given 11 days after radioembolization (4 events in 6 patients) than after 14 days (no events in 3 patients) when assessments were carried out for at least 30 days after commencement of sorafenib [15]. These results defined the optimal duration of 14 days between radioembolization and sorafenib treatment for the subsequent Phase II study. All patients from the Phase I study (recruited using the same inclusion/exclusion criteria) and treated with sorafenib from day 14 and followed-up using the same study design were also included in the efficacy and safety analyses for the Phase II study.

Both studies were conducted in accordance with ISO-14155-1 (2003), the World Medical Association Declaration of Helsinki and all applicable local regulations. Study protocol was approved by each institute's Human Research Ethics Committee namely, the Centralised Institutional Review Board (CIRB), SingHealth, Singapore; the Medical Research & Ethics Committee, Ministry of Health Malaysia; Institutional Review Board, Yangon GI & Liver Centre, Yangon; and Institutional Review Board of Seoul National University Bundang Hospital. Patients were informed of the nature of the study and provided written informed consent.

### Patients

The protocol for this trial and supporting TREND checklist are available as supporting information (see Protocol S1 and Checklist S1).

Patients with inoperable HCC including those with extrahepatic disease (except CNS metastases) and/or major vascular involvement (i.e. both Barcelona Clinic for Liver Cancer [BCLC] stages B and C) were eligible for inclusion. A confirmatory diagnosis of HCC was based on histology, or by meeting radiological criteria for HCC by dynamic contrast-enhanced computed tomography [CT] or magnetic resonance imaging [MRI]), with supporting evidence based on positive serology for hepatitis B or C virus, or serum alpha-fetoprotein above normal range (≥400 µg/L) [16], [17]. All patients were ≥18 years of age, had measurable disease (defined as ≥1 lesion of ≥10 mm), adequate renal function (creatinine ≤2.0 mg/dL), hemopoietic function (leukocytes ≥2,500/µL; neutrophils ≥1,500/µL; platelets ≥50,000/µL; hemoglobin >9.5 g/dL), and Eastern Cooperative Oncology Group (ECOG) performance status 0 or 1. In addition, eligible patients were required to have: 1) sufficient liver function for safe delivery of radioembolization, defined as: an absence of ascites or synthetic liver dysfunction (total bilirubin <2.0 mg/dL [<34.2 µmol/L]), International Normalized Ratio (INR)≤2.0; albumin ≥2.5 g/dL and aspartate transaminase (AST), alanine transaminase (ALT) and alkaline phosphatase (ALP) each ≤5× upper limit of normal; 2) hepatic arterial anatomy that would enable safe delivery of microspheres to the liver only; 3) without excess hepato-pulmonary shunting (>20%); or 4) without main trunk portal vein thrombosis (PVT). Premenopausal, sexually-active individuals were required to use two forms of contraception during the study. Patients were excluded if they were pregnant or breast feeding or had been previously treated with external beam radiotherapy to the liver or were currently receiving any other investigational agent.

### Radioembolization

Radioembolization is a form of brachytherapy during which ^90^Y microspheres are delivered via a temporary transfemoral catheter advanced under fluoroscopic guidance into the hepatic artery branches that supply the hepatic lesions. Pre-treatment planning and treatment is undertaken in the angiography suite by an interventional radiologist. Details of the procedure and post-procedure supportive care associated with ^90^Y-resin microspheres (SIR-Spheres; Sirtex Medical Limited, North Sydney, Australia) administration have been previously described [3], [18]. Prior to treatment, eligible patients underwent CT or MRI imaging to determine the extent of hepatic and extra-hepatic disease. A hepatic angiography was then conducted to map the hepatic arterial anatomy, coil embolize vessels as required, and determine the extent of hepato-pulmonary shunting and uptake in tumor following administration of technetium-99m macroaggregated albumin (^99m^Tc-MAA). Planar imaging of ^99m^Tc-MAA was used for treatment planning and calculating the tumor-to-normal (T∶N) ratio, with Single Photon Emission Computed Tomography (SPECT) imaging employed in cases where further information was needed for the accurate assessment of the extent of multifocal disease. Radioembolization activity (in gigabecquerels [GBq]) was calculated using the Partition Model [19], where feasible, or Body Surface Area (BSA) method [20] when there was multifocal disease for which discrete regions of interest could not be applied or clearly defined. For activity calculations using the Partition Model, the distribution of ^99m^Tc-MAA during the simulation were assumed to be identical to ^90^Y-resin microspheres, and the activity was calculated in discrete “areas-of-interest” for the tumor, normal parenchyma and lung compartments, limiting the maximum permitted exposure for the non-tumoral liver compartment to 70 Gy [19] and lung exposure to 30 Gy. On the day of treatment, ^90^Y-resin microspheres were selectively infused into the affected lobe(s) or segment(s), or whole liver via a micro-catheter placed in the hepatic artery [14].

### Sorafenib

Sorafenib (400 mg twice-daily) was initiated 14 days post-radioembolization and then given continuously until tumor progression or the emergence of drug-related adverse events. Guidelines for dose adjustments and dose interruptions to sorafenib were as per the standardized schedule reported in the Sorafenib Hepatocellular Carcinoma Assessment Randomized Protocol (SHARP) study [6] which required discontinuation after two dose reductions (first to 400 mg once daily and then to 400 mg every two days).

### Assessment and follow-up

Assessments were made at baseline, 2 weeks post-radioembolization and thereafter at 4-weekly intervals. Baseline imaging assessment was conducted just prior to the start of study therapy and every 3 months or at the investigator's discretion until disease progression. If a complete or partial response was detected on CT, then a confirmatory CT scan was performed between 28 and 35 days later. All responding patients were regularly assessed for eligibility of radical therapy. Patients who progressed were assessed at 12-weekly intervals until death or 18 months after the end of the study. Adverse events and their severity and relationship to the study treatment were recorded from the date of consent to 28 days after the last dose of sorafenib. Toxicity was assessed using the National Cancer Institute's Common Terminology Criteria for Adverse Events (CTCAE) version 3.0.

### Endpoints and statistical analysis

The primary endpoints were both safety/tolerability and best overall response rate (ORR), using RECIST version 1.0. Secondary endpoints were: disease control rate (DCR), progression-free survival (PFS), overall survival (OS) and health-related quality of life (HRQoL) using the EuroQol 5-Dimensions (EQ-5D) Index.

Adverse events were reported from the date of radioembolization, when sorafenib therapy started, then at monthly intervals thereafter. If an adverse event increased in severity over the next defined interval, it was recorded as a new event in the next interval. PFS and OS were measured from study entry. HRQoL was evaluated at study entry, every month during the treatment period and at 6-month intervals thereafter [21].

The sample size for Phase II was computed using the A'Hern single-stage design (2001) [22]. Assuming a target best ORR of 30% and a no-further-interest ORR of 10%, with type I error of 5% and power of at least 80%, a sample size of at least 25 patients would be required. These 25 patients will include 3 to 6 already recruited under the appropriate Phase I cohort. The study protocol allowed for a maximum of 35 patients to be recruited, which allows for possible lost to follow-up. A best ORR of at least 24% was required to conclude potential efficacy. Best ORR was calculated with 95% exact confidence intervals (CI). Baseline patient characteristics, ORR, DCR, PFS and OS were stratified by BCLC stage to allow meaningful comparisons with other treatment modalities. PFS and OS were summarized using the Kaplan-Meier technique; median values and 95% CI were reported.

A graphical plot was used to explore the pattern of HRQoL over time. To reduce the influence of extreme values in the graphical plot, locally weighted regression was used for smoothing EQ-5D index [23]. A Mixed-effect model for repeated-measures data was also performed for EQ-5D index. The model included a patient-specific random intercept, together with the following fixed predictors: baseline EQ-5D index; BCLC stage, and interaction between BCLC stage and time as a continuous variable with a linear trend. This parameterization estimates a separate intercept and linear time trend for each BCLC stage while adjusting for the corresponding baseline EQ-5D index. SAS version 9.2 (SAS Institute, NC, USA) was used for all analyses.

## Results

### Patients

**Figure 1:**
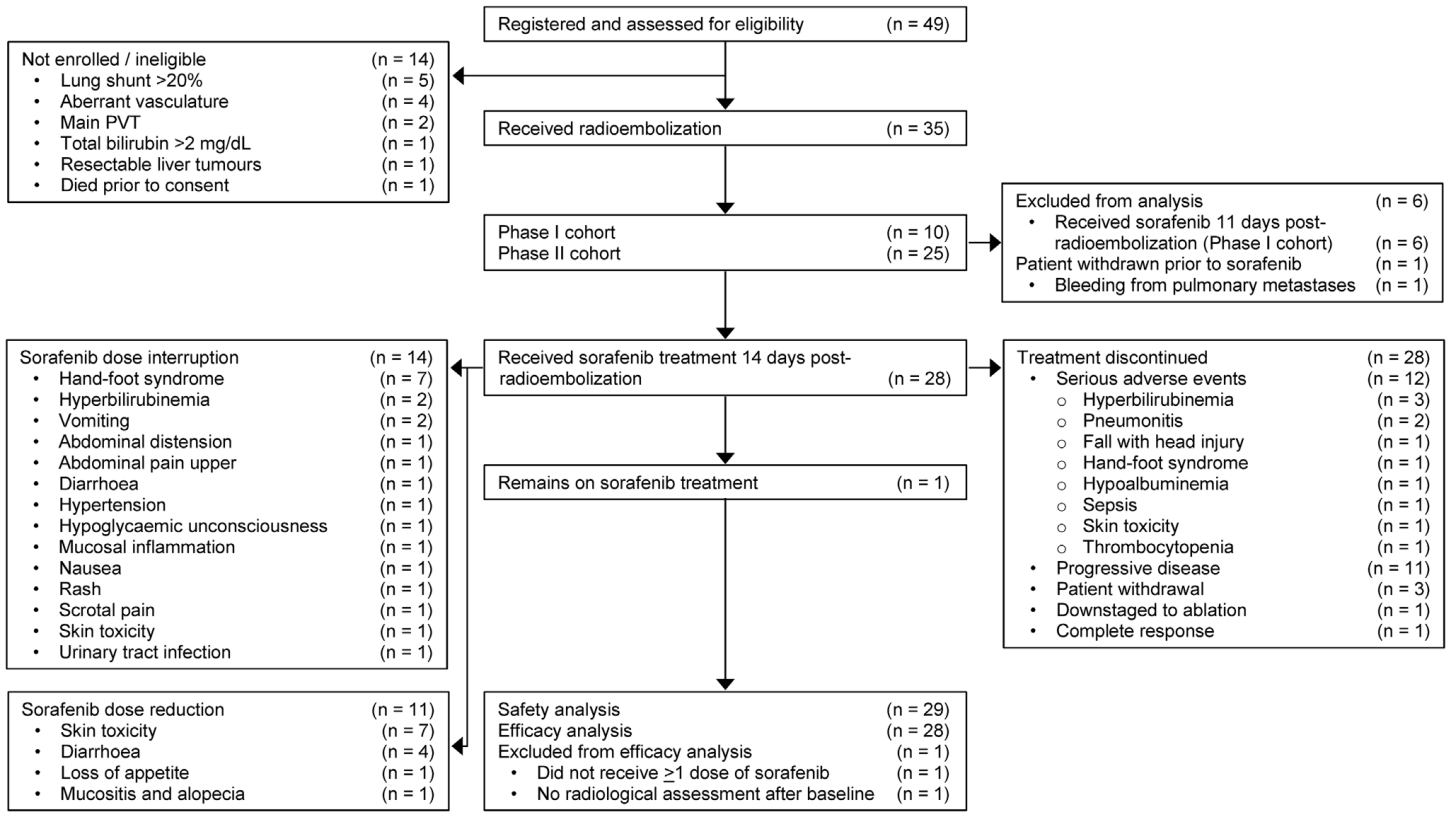
CONSORT diagram of Phase II trial of radioembolization followed by sequential sorafenib in patients with HCC.

Between June 2008 and May 2009, 49 patients were assessed for eligibility and 29 patients were enrolled (including 4 patients from the initial Phase I study who had received sorafenib on day 14), received radioembolization and were included in the intention-to-treat analysis for safety (see CONSORT diagram; Figure 1). Sorafenib was contraindicated in one patient with bleeding due to pulmonary metastases and did not receive any further CT scans after the baseline assessment and was excluded from the efficacy analyses. Median follow-up was 10.9 months (range, 2.1–33.8 months). Patient characteristics of 29 patients are summarized in Table 1. Approximately two-thirds of patients had BCLC stage C disease (62%); of whom 57% had macrovascular invasion and 61% extra-hepatic disease. Most (69%) had >25% of the liver volume effected by tumor and/or pre-existing liver dysfunction (41% albumin <35 g/L; 14% total bilirubin >1.2 mg/dL); 7 (24%) had received prior liver-directed therapy (resection, RFA, TACE or ^131^I-lipiodol).

**Table 1:** Baseline patient and treatment characteristics.

Characteristics		BCLC stage B (N = 11)	BCLC stage C (N = 18)	Overall(N = 29)
**Gender, N (%)**	Male	7 (64)	14 (78)	21 (72)
	Female	4 (36)	4 (22)	8 (28)
**Ethnic group, N (%)**	Chinese	5 (45)	15 (83)	20 (69)
	Malay	0 (0)	1 (6)	1 (3)
	Indian	0 (0)	1 (6)	1 (3)
	Myanmar	6 (55)	0 (0)	6 (21)
	Korean	0 (0)	1 (5.6)	1 (3.4)
**Age, years, mean ± SD**		62.6±14.8	65.8±7.2	64.6±10.6
**Prior Procedures, N (%)**	Total	3 (27)	4 (22)	7 (24)
	Surgical resection	2† (18)	3† (17)	5 (17)
	Ablative (RFA)	1 (9)	0 (0)	1 (3)
	Vascular (TACE)	0 (0)	2 (11)	2 (7)
	^131^I-lipiodol	0 (0)	1 (4)	1 (3)
**Child-Pugh class, N (%)**	A	10 (91)	10 (56)	20 (69)
	B	1 (9)	8 (44)	9 (31)
**ECOG performance status, N (%)**	0	11 (100)	11 (61)	22 (76)
	1	0 (0)	7 (39)	7 (24)
**Macro-vascular invasion, N (%)**		0 (0)	8 (57)*	8 (32)
**Extra-hepatic spread, N (%)**		0 (0)	11 (61)	11 (38)
**TNM stage, N (%)**	I	2 (18)	0 (0)	2 (7)
	II	5 (45)	0 (0)	5 (17)
	IIIA	4 (36)	7 (39)	11 (38)
	IV	0 (0)	11 (61)	11 (38)
**Total bilirubin**	mean, mg/dL	0.70	1.06	0.93
	>1.2 mg/dL, N (%)	0 (0)	4 (22)	4 (14)
**Albumin**	mean, g/L	31.7	30.3	30.8
	<35 g/L, N (%)	3 (27)	9 (50)	12 (41)
**Radioembolization: ^90^Y activity administered, GBq**	median (IQR)	2.0 (1.5)	3.0 (0.7)	3.0 (1.0)
**Target treatment, N (%)**	Whole liver	7 (64)	13 (72)	20 (69)
	Right lobe	4 (36)	5 (28)	9 (31)
**Target tumor volume, mL**	median (IQR)	336 (488)	786 (1021)	484 (944)
**Target liver volume, mL**	median (IQR)	1282 (813)	2254 (1368)	1843 (1186)
**Sorafenib daily dose per patient, mg**	median (IQR)	600.0 (324.6)	638.8 (319.7)	600.0 (319.7)
**Sorafenib treatment duration, months,**	median (IQR)	6.9 (7.3)	3.0 (3.9)	4.1 (4.8)
**Sorafenib patients receiving >80% planned dose, N (%)**		4 (36)	7 (39)	11 (38)

* Four patients have missing information in the BCLC stage C group;

† One patient in each cohort received repeat surgical resections.

### Dosing

Planar and SPECT imaging were used for treatment planning in 25 and 4 patients, respectively. The Partition Model was used for the calculation of administered ^90^Y activity in all patients. The mean T∶N ratio was 4.8 (SD ± 3.5). The treatment approach reflected the tumor burden and distribution of tumors within the liver. Patients received a median activity of 3.0 GBq (interquartile range [IQR], 1.0), by whole-liver (69%) and right-lobe (31%) infusion. Median target liver and tumor volumes were 1843 mL (IQR: 1186) and 484 mL (IQR: 994), respectively. Mean lung shunting was 8.1%. A median of 600 mg (range, 127–791) sorafenib was administered daily over a median of 4.1 months (range, 0–20.4) (Table 1). The median daily sorafenib dose was 676 mg (month 1), 665 mg (month 2), 641 mg (month 3) and 566 mg thereafter. Sorafenib dose discontinuations and dose reductions were experienced in 4% and 39% of patients overall, and by 0% and 64% of patients with BCLC stage B, and by 6% and 24% of patients with BCLC stage C, respectively.

### Safety and tolerability

**Figure 2:**
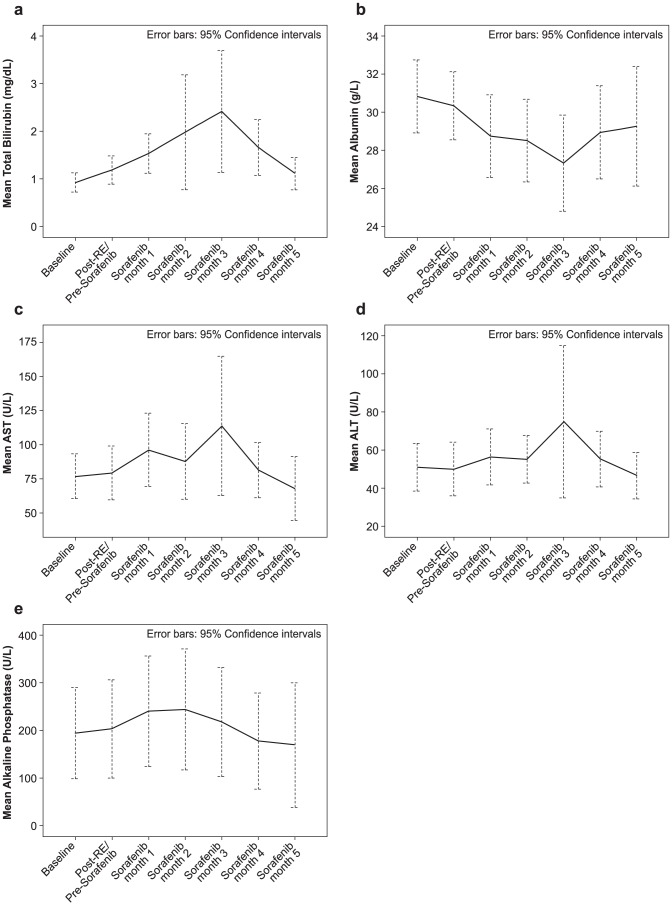
Liver function tests, stratified by time, for (A) total bilirubin, (B) albumin, (C) AST, (D) ALT and (E) alkaline phosphatase at baseline and after radioembolization followed by sorafenib. Post-RE: Post-radioembolization/Pre-sorafenib period of 14 days.

Treatment-related toxicities and mean ±95% CI changes from baseline liver function tests are presented in Tables 2 and 3, plus Figure 2, respectively. Twenty-eight of 29 (97%) patients experienced ≥1 toxicity following the treatment; 15 (52%) were grade 3 or higher. Toxicities in 5 (17%) patients occurred post-radioembolization and prior to sorafenib administration; all were grade 1–2 except one grade 3 ascites.

**Table 2:** Treatment-related toxicities,* stratified by time.

	Overall incidence (N = 29)		Post-RE and		Post-sorafenib							
			Pre-sorafenib (N = 9)		Month 1 (N = 29)		Month 2 (N = 29)		Month 3(N = 27)		Month 4+ (N = 27)	
Grade, N (%) of patients	1–2	≥3	1–2	≥3	1–2	≥3	1–2	≥3	1–2	≥3	1–2	≥3
**Any**	13 (45)	15 (52)	4 (14)	1 (3)	12 (41)	7 (24)	6 (21)	3 (10)	5 (19)	3 (11)	10 (37)	4 (15)
**Blood and lymph**												
Anemia	1 (3)				1 (3)							
Leukopenia	1 (3)				1 (3)							
Thrombocytopenia	1 (3)								1 (4)			
**Gastrointestinal**												
Abdominal distension		1 (3)						1 (3)				
Ascites		1 (3)		1 (3)								
Diarrhea	9 (31)				6 (21)		1 (3)				2 (8)	
Nausea	2 (7)				1 (3)				1 (4)			
Upper GI hemorrhage		1 (3)										1 (4)
Vomiting	3 (10)	1 (3)	3 (9)					1 (3)				
**General/administration site**												
Lethargy	1 (3)				1 (3)							
Mucosal inflammation	1 (3)								1 (4)			
Pyrexia	1 (3)		1 (3)		1 (3)							
**Hepatobiliary**												
Elevated ALP	1 (3)	1 (3)	1 (3)			1 (3)						
Elevated ALT	1 (3)		1 (3)									
Elevated AST	1 (3)	2 (7)	1 (3)			1 (3)			1 (4)	1 (4)		
Hepatitis	1 (3)								1 (4)		1 (4)	
Hyperbilirubinemia		3 (10)			1 (3)	1 (3)	1 (3)	1 (3)		2 (7)		
Hypoalbuminemia		1 (3)				1 (3)						
**Infections and infestations**												
Sepsis		1 (3)				1 (3)						
Viral infection		1 (3)				1 (3)						
**Injury, poisoning and procedural complications**												
Fall		1 (3)										1 (4)
Radiation skin injury	1 (3)		1 (3)									
Skin toxicity		1 (3)				1 (3)						
**Metabolism and nutrition**												
Decreased appetite	1 (3)				1 (3)							
**Renal and urinary**												
Urinary tract infection	1 (3)						1 (3)					
**Reproductive system**												
Scrotal pain	1 (3)						1 (3)					
**Respiratory, thoracic and mediastinal**												
Chest discomfort					1 (3)							
Hemoptysis		1 (3)		1 (3)								
Pneumonia		1 (3)		1 (3)								
Pneumonitis	1 (3)	1 (3)†								1 (4)	1 (4)†	
**Skin and subcutaneous**												
Acne	1 (3)								1 (4)			
Alopecia	6 (21)				2 (7)		1 (3)				3 (10)	
Hand-foot syndrome	7 (24)	5 (17)			5 (17)	2 (7)	1 (3)	1 (3)	1 (4)		3 (10)	2 (7)
Rash	5 (17)		1 (3)		3 (10)		2 (7)					
**Vascular**												
Gingival bleeding	1 (3)										1 (4)	
Hypertension	2 (7)								1 (4)		2 (7)	

* Treatment-related toxicities included all those assessed as either definitely, probably, possibly related to treatment and unlikely to be related to treatment (excluding only those events assessed as definitely unrelated to treatment); If a toxicity occurred multiple times to the same patient, it was counted once for that patient at the highest grade that was assessed.Post-RE: Post-radioembolization/Pre-sorafenib period of 14 days; N = number of patients alive at each time interval; National Cancer Institute Common Terminology Criteria for Adverse Events (CTCAE) version 3.

Abbreviations: ALP: alkaline phosphatase; ALT: alanine transaminase; AST: aspartate aminotransferase GI: gastrintestinal.

† This was a grade 5 event.

**Table 3:** Comparison of laboratory adverse events by severity from baseline to 90 days post-treatment.

	N (%) patients (N = 29)		N (%) patients (N = 29)	
	Pre-Treatment		≤90 days Post-Radioembolization	
**Grade**	1–2	≥3	1–2	≥3
Total Bilirubin	2 (7)	0	14 (48)	2 (7)
Albumin	22 (76)	0	20 (69)	5 (17)
Alanine transaminase	17 (59)	0	20 (69)	1 (3)
Aspartate aminotransferase	21 (72)	1 (3)	20 (69)	8 (28)
Alkaline phosphatase	9 (31)	1 (3)	13 (45)	4 (14)

National Cancer Institute Common Terminology Criteria for Adverse Events (CTCAE) version 3; includes all events occurring up to and including 90 days post-radioembolization.

Twenty-seven patients (93%) experienced toxicities following sorafenib administration during month 1 (66% any grade; 24% grade ≥3), month 2 (31% any grade; 10% grade ≥3), month 3 (30% any grade; 11% grade ≥3), through month 4 and beyond (52% any grade; 15% grade ≥3). These toxicities resulted in sorafenib dose reduction in 11 (39%) and discontinuation in 1 (4%) of patients (Figure 2 and Table 4). Two patients experienced serious disabling/incapacitating hand-foot syndrome which resolved with active management over 1–2 months in both cases. The median duration of severe (n = 5; 17%) and any (n = 12; 41%) hand-foot syndrome was 19 days and 35 days, respectively. Diarrhea (all grade 1 or 2) was recorded in 9 (31%) patients over a median duration of 70 days.

**Table 4:** Sorafenib dose modifications.

Dose modification	BCLC stage B (N = 11)	BCLC stage C (N = 17)	Overall (N = 28)
Dose delay, N (%)	6 (55)	10 (59)	16 (57)
Dose further delay, N (%)	0	1 (6)	1 (4)
Dose reduction, N (%)	7 (64)	4 (24)	11 (39)
Dose resumed to starting dose, N (%)	1 (9)	2 (12)	3 (11)
Dose permanently discontinued following modification, N (%)	0	1 (6)	1 (4)

Two patients experienced serious liver-related adverse events which may have been related to treatment. Both cases of serious liver-related adverse events were secondary to disease progression and resolved with active management over 2.5 weeks and 3 months, respectively. A third patient with abdominal extension and symptoms of confusion and jaundice due to hyperbilirubinemia and infection (which may have been treatment-related) was hospitalized, received antibiotic treatment and sorafenib treatment was temporarily interrupted; symptoms were recorded over 4 days. The duration of severe (grade 3+) changes in bilirubin in 2 patients (7%) was recorded over a median of 25 days. One patient had severe upper gastrointestinal hemorrhage at 6.3 months and 7.6 months after the initiation of sorafenib therapy which lasted 8 days and 3 days, respectively. The duration of mild (grade 1–2) radiation skin injury in one patient was 11 days.

One patient with progressive disease died 3 months post-treatment due to respiratory distress attributed to therapy. The patient had a 17% lung-shunt fraction and was administered 3.0 GBq ^90^Y. The pulmonary radiation exposure was 25 Gy. This patient had an unresolved grade 2 sorafenib-related hand-foot syndrome at 1 month post-treatment, before presenting with respiratory symptoms at 2.5 months, whereupon sorafenib was discontinued. The patient died two weeks later. A further patient with a lung dose of 15 Gy was reported to have mild (grade 1) pneumonitis 4.7 months post-radioembolization.

### Response rates

Best overall response (complete response or partial response) was observed in 7 of 28 patients (25%; 95% CI, 11–45%), which met the pre-determined criteria of 7 responses for potential efficacy. There were 2 (7%) complete responses, 5 (18%) partial responses, 15 (54%) stable disease and 5 (18%) progressive disease. The disease control rate was 79% (95% CI, 59–92%) overall, and 100% (11 of 11 cases) and 65% (11 of 17 cases) in BCLC stage B and C, respectively. Ten (59%) of the 17 patients with BCLC stage C had extrahepatic spread; disease control beyond the liver was not evident as assessed by RECIST 1.0.

Two patients (7%) with BCLC stage B had a sufficient tumor response to enable radical therapy; both patients received RFA and were censored at the time of the procedures.

### Progression and overall survival

**Figure 3:**
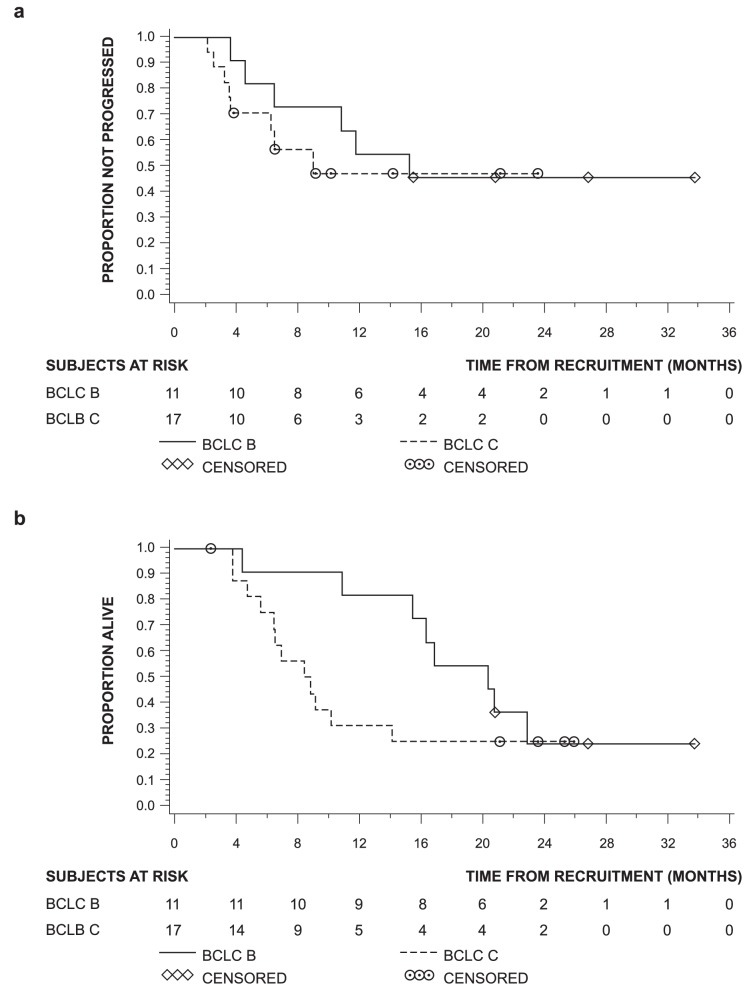
(A) Time to progression and (B) overall survival.

Median time to progression for BCLC stage B was 15.2 months and 9.0 months for BCLC stage C (Table 5, Figure 3). Median PFS for BCLC stage B and C patients were 15.2 and 6.5 months, respectively.

**Table 5:** Summary of efficacy measures.

	BCLC stage B (N = 11)	BCLC stage C (N = 17)	Overall (N = 28)
**Best overall response rate**, N (%) patients			
Complete response (CR)	1 (9%)	1 (6%)	2 (7%)
Partial response (PR)	4 (36%)	1 (6%)	5 (18%)
Stable response (SD)	6 (55%)	9 (53%)	15 (54%)
Progressive disease	0	5 (29%)	5 (18%)
Not done	0	1 (6%)	1 (4%)
**Overall response rate** (CR+PR), % patients (95% CI)	46% (17–77)	12% (2–36)	25% (11–45)
**Disease control rate** (CR+PR+SD), % patients (95% CI)	100% (72–100)	65% (38–86)	79% (59–92)
**Time to Progression**, months, median, (95% CI)	15.2 (4.6–nr)	9.0 (3.5–nr)	not applicable
**Progression-free survival**, months, median (95% CI)	15.2 (4.6–nr)	6.5 (3.5–9.1)	not applicable
**Overall survival**, months, median (95% CI)	20.3 (10.9 nr)	8.6 (5.6–14.2)	not applicable

CI: confidence interval nr: not reached.

Median overall survivals for BCLC stage B and C patients were 20.3 and 8.6 months, respectively.

### Health-related Quality of life

**Figure 4:**
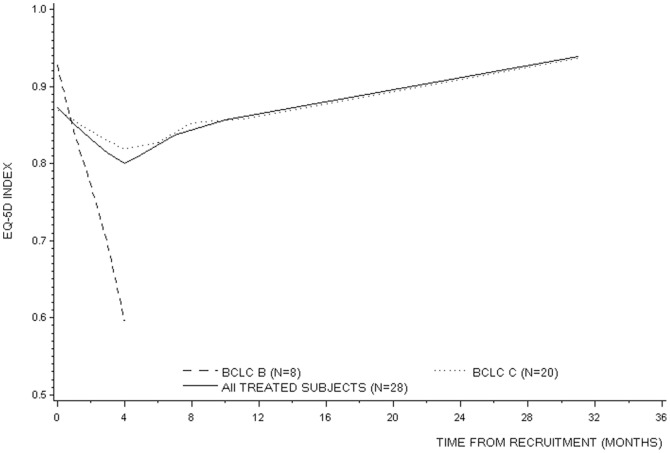
Smoothed EQ-5D quality of life index over time stratified by BCLC stage.

Figure 4 shows the smoothed EQ-5D index using locally-weighted regression over time in patients stratified by BCLC stage. The evolution of patient's EQ-5D index over time was positively associated with their respective baseline EQ-5D index (Beta coefficient = 0.374). EQ-5D index in BCLC Stage B decreased over time (Beta coefficient = −0.004), while it increased in BCLC Stage C (Beta coefficient = 0.001).

## Discussion

This study represents the first prospective Phase II evaluation of sequential radioembolization-sorafenib therapy in patients from Asia-Pacific region. The majority of patients included in this trial had advanced HCC and a high tumor burden in the liver (median tumor volume in the liver 448 mL), and were not ideal candidates for TACE. The combination of radioembolization-sorafenib appears to be manageable and consistent with previously published experience with each treatment [6], [14], [24]. Excluding hand-foot syndrome, 23% of events were grade 3 or above. Most events were transient and managed with sorafenib dose adjustments or discontinuation. By comparison, treatment-emergent adverse events were reported in 98% of patients (including 39% with grade 3 and 6% with grade 4 events) in the Sorafenib Hepatocellular Carcinoma Assessment Randomized Protocol (SHARP) study [6]. The evaluation of the combination of doxorubicin-eluting beads (DEB) with sorafenib found that most patients experienced at least one grade 3 to 4 toxicity, the most common being grade 3–4 fatigue in 36% of patients, and required dose reductions in 73% of patients [25]. By comparison, the most commonly reported adverse event with radioembolization (in a similar cohort of 325 patients) was fatigue occurring in 54% of patients, including 2% with grade 3 events, with an increase in total bilirubin reported as the most commonly reported grade 3+ event in 6% of patients at 3 months post-treatment [14].

The incentive for therapeutic intervention to palliate symptoms or extend survival of HCC must be balanced against the degree of hepatic functional reserve and the ability of the patient to tolerate the procedure [26], [27]. Compromised hepatic function as manifested by thrombocytopenia, excessive elevations in transaminases and bilirubin, jaundice and ascites were reported in 14% of patients following radioembolization-sorafenib in this study. These cases resolved upon withdrawal of sorafenib and the administration of steroid therapy.

Liver dysfunction with sorafenib is a rare event [28], [29](occurring in <1% of the sorafenib-treated patients in the Asia-Pacific trial) [5]. The risk of radioembolization-induced liver disease (REILD) reported by Sangro et al 2008 [30] increased significantly with high total bilirubin (>3 mg/dL), jaundice and ascites in the absence of overt tumor progression and/or bile duct dilatation. As advised by Lau *et al* 2012, the dose for uninvolved, normal parenchyma should never be >70 Gy and should preferably remain <50 Gy with some institutions, especially in Asia, having set even lower thresholds of 40–43 Gy [31]. Further study by Sangro *et al* 2013 has shown that the frequency and severity of this complication can be significantly reduced through modifications to the activity calculations (which overall lowered the prescribed ^90^Y activity), combined with lowering the threshold for radioembolization from a total bilirubin of 3 to 2 mg/dL and the routine use of ursodeoxycholic acid and low-dose steroid over the two months post-radioembolization [32].

There was one case of thrombocytopenia which was a modest (grade 1) event at 3.5 months post-treatment followed by patient death due to progressive disease 2 months later. Thrombocytopenia has been commonly observed in HCC patients following sorafenib administration [6] and has been rarely reported following radioembolization [30], [33], [34].

Pneumonitis is an uncommon event (≥1/1000 to <1/100) associated to sorafenib treatment [6] and is associated with excessive lung radiation secondary to pulmonary shunting of ^90^Y-microspheres [19], [35]. The case of radiation pneumonitis reported in this study was attributed to treatment and, as a result, sorafenib was discontinued at 2.5 months post-treatment. The estimated pulmonary radiation exposure was 25 Gy, slightly below the recommended threshold of 30 Gy in order to mitigate the risk of pulmonary tissue damage. The patient had no prior history of chronic obstructive pulmonary disease that would have increased the risk of lung tissue damage.

The nature and frequency of serious adverse events observed in the current study are not unexpected for this population of HCC patients with advanced disease against a background of cirrhosis, two-thirds of whom presented with macrovascular invasion, extra-hepatic disease and/or liver dysfunction [5], [14], [24]. In a European Phase II study including a similar proportion of patients with BCLC stage B and C, Mazzaferro *et al.* 2013 [27] recently reported a 23% and 36% rate of liver decompensation at 3 and 6 months, respectively after radioembolization. While investigators from Chicago observed that in patients with PVT, 55% of patients decompensated from Child-Pugh A to B by the time of progression at 5.6 months after radioembolization [26]. The one case of possible radiation/drug-induced liver disease who expired approximately 3.5 months after commencing therapy points to the tenuous condition that these patients often present with.

The limitations of this study are its small size and single-arm design. There was a significant overlap between the patient population in this study and other published studies with sorafenib in predominantly advanced HCC, thus allowing for meaningful comparisons. The response rate of 25.0% (including 7% with CR) and corresponding disease control rate of 79% (by RECIST 1.0 criteria) with radioembolization-sorafenib combination is consistent with experience with radioembolization alone (response rate [40%, including 10% with CR] and disease control rate [79%] by EASL criteria [27]) and compares favorably with the 2–9% partial response and 35–95% disease control rate of sorafenib alone or in combination with either conventional or drug-eluting TACE (RECIST 1.0 criteria) [5], [6], [25], [36]. The median overall survival of 20.3 months for BCLC stage B and 8.6 months for BCLC stage C patients in the current study also compare favorably with the overall survival following bland embolization in Asia-Pacific patients with intermediate or advanced HCC (median 18.2 and 6.8 months, respectively) [37], as well as for sorafenib both in intermediate and predominately advanced patients in the SHARP study (median 14.5 and 9.7 months, respectively) and in the advanced population represented in the Asia-Pacific study (median overall survival 6.5 months; 5.6 months in those with PVT and/or extra-hepatic disease) [5], [24], [38]. Selective delivery of internal radiation therapy (proportionate to the burden and spread of tumors in the liver) in conjunction with the anti-proliferative and anti-angiogenic properties afforded by sorafenib may provide a benefit greater than that afforded by either agent alone. Further investigations are ongoing in a European multicenter randomized Phase III study (SORAMIC: Sorafenib in combination with local micro-therapy guided by Gd-EOB-DTPA-enhanced MRI in patients with inoperable hepatocellular carcinoma) designed to compare overall survival following sorafenib alone versus sequential radioembolization-sorafenib in patients with intermediate- or advanced-stage HCC (NCT001126645). In addition, radioembolization is being compared to sorafenib in two Phase III trials in Asia-Pacific and European patients with HCC (NCT01135056; NCT01482442).

In summary, the results of the current study provide provisional evidence of the potential efficacy and manageable toxicity of sorafenib and radioembolization in a population with predominantly advanced disease. Further investigation of radioembolization and sorafenib in randomized multicenter trials are now ongoing (NCT001126645, NCT01135056, NCT01482442, NCT01556490) for patients with intermediate- and advanced-stage HCC as well as for pre-transplant HCC (NCT00846131).

## Supporting Information

Protocol S1 **Trial Protocol.**
(PDF)

Checklist S1 **TREND checklist.**
(PDF)
