# Supplementary material for: Multicenter Phase II Study of Sequential Radioembolization-Sorafenib Therapy for Inoperable Hepatocellular Carcinoma
Source: PLoS One. 2014 Mar 10;9(3):e90909. doi: 10.1371/journal.pone.0090909 (PMC3948695; doi:10.1371/journal.pone.0090909)
Supplement: Protocol S1 — Trial Protocol. (PDF) [file pone.0090909.s001.pdf]

Asia-Pacific

Hepatocellular Carcinoma Trials Group

Protocol AHCC05

PHASE I/II STUDY OF SIR-SPHERES® PLUS SORAFENIB AS FIRST LINE TREATMENT IN PATIENTS WITH NON-RESECTABLE PRIMARY HEPATOCELLULAR CARCINOMA

SUMMARY FOR PROTOCOL AMENDMENT

Protocol Title: Phase I/II Study of SIR-Spheres Plus Sorafenib as First Line Treatment in Patients with Non-Resectable Primary Hepatocellular Carcinoma

Approved Version: Version 7.0, dated 11 Dec 2008
Amended Version: Version 8.0, dated 28 Mar 2009

S/N	PAGE NO. INVOLVED	DESCRIPTION	AMENDED FROM	AMENDED TO
			VERSION 7.0 DATED 11 DEC 2008	VERSION 8.0 DATED 28 MAR 2009
1	1	**Title**	**December 2008**	**March 2009**
2	2	**Study In-charge Title**	**Principal Investigator**	**Protocol Chair**
3	3	**Participation of the following invited centers is pending the approval of their IRB**	**1. China (Beijing) –** *Cancer Institute Hospital*;<br>**2. India –** *TATA Memorial Centre*;<br>**3. Indonesia (Bali, Jakarta) –** *University of Udayana, University of Indonesia*;<br>**4. Korea (Seoul, Suwon) –** ~~*Seoul National Bundang Hospital*~~, *St Vincent Hospital*;<br>**5. Malaysia (Selangor) –** ~~*Hospital Selayang,*~~ *University Malaya Hospital*;<br>**6. ~~Myanmar (Yangon);~~**<br>**7. New Zealand (Auckland) –** *University of Auckland*;<br>**8. Philippines (Manila, Davao City) –** *Santo Toma, Davao Doctors Hospital*;<br>**9. Singapore –** ~~***SGH,***~~ ~~***NCC***~~, *NUH, CGH, TTSH, AH*;<br>**10. Taiwan –** *Chang Gung Memorial Hospital*;<br>**11. Thailand (Bangkok) –** *National Cancer Institute*;<br>**12. Vietnam (Ha Noi, Ho Chi Minh City) –** *Cho Ray Hospital, K Hospital, Phu Tho Hospital, Viet Duc University Hospital*	**1. China (Beijing) –** *Cancer Institute Hospital*;<br>**2. India –** *TATA Memorial Centre*;<br>**3. Indonesia (Bali, Jakarta) –** *University of Udayana, University of Indonesia*;<br>**4. Korea (, Suwon) –***St Vincent Hospital*;<br>**5. Malaysia (Selangor) –**, *University Malaya Hospital*;<br>**6. New Zealand (Auckland) –** *University of Auckland*;<br>**7. Philippines (Manila, Davao City) –** *Santo Toma, Davao Doctors Hospital*;<br>**8. Singapore –** *NUH, CGH, TTSH, AH*;<br>**9. Taiwan –** *Chang Gung Memorial Hospital*;<br>**10. Thailand (Bangkok) –** *National Cancer Institute*;<br>**11. Vietnam (Ha Noi, Ho Chi Minh City) –** *Cho Ray Hospital, K Hospital, Phu Tho Hospital, Viet Duc University Hospital*<br><br>**Section "Participation of the following invited centers is pending the approval of their IRB" shifted from page 3 to page 5.**
4	3b	**Steering Committee**	**Assoc. Prof. Pierce Chow Kah Hoe<br>Duke-NUS Graduate Medical School<br>Singapore<br>2 Jalan Bukit Merah<br>Singapore 169547<br>Tel: +65 6516 7666 Fax: +65 6224 6242<br>Email: pierce.chow@gms.edu.sg**	**Assoc. Prof. Pierce Chow Kah Hoe<br>Singapore General Hospital<br>Outram Road<br>Singapore 169608<br>Duke-NUS Graduate Medical School<br>Singapore<br>2 Jalan Bukit Merah<br>Singapore 169547<br>Tel: +65 63214051 Fax: +65 62209323<br>Email: gsupc@singnet.com.sg**

5	4	Participating Centers	The original text do not have this section	Study Principal Investigator<br>Assoc. Prof. Pierce Chow Kah Hoe<br>Senior Consultant Hepato-Biliary Surgeon<br>Singapore General Hospital, Outram Road<br>Singapore 169608<br>Tel: +65 63214051 Fax: +65 62209323<br>Email: gsupc@singnet.com.sg<br><br>Seoul National University Bundang Hospital<br>Prof Han Ho Seong<br>Seoul National University Bundang Hospital<br>300 Gumi-dong, Bundang-gu, Seongnam-si,<br>Gyeonggi-do 463-707, Korea<br>Tel: +031-787-4055 Fax: +031-787-4055<br>Email: hanhs@snubh.org<br><br>Selayang Hospital<br>Datuk (Dr) Harjit Singh<br>Department of Hepato-Pancreato-Biliary Surgery<br>Hospital Selayang<br>Lebuhraya Selayang-Kepong<br>Batu Caves<br>68100 Selangor, Malaysia<br>Tel: +603-6120 2122 Fax: +603-6120 7564<br>Email: harjit@selayanghospital.gov.my<br><br>Yangon GI and Liver Center<br>Prof Khin Maung Win<br>No. 191 – 193<br>30th Street, Upper Block<br>Pabedan Township<br>Yangon, Myanmar<br>Tel: +95-1-256128 Fax: +95-1-222965<br>Email: 30thstreetclinic@mptmail.net.mm

<table>
<tr><td>5</td><td>4</td><td>Participating Centers</td><td>The original text do not have this section</td><td>Singapore General Hospital
Dr. Alexander Chung Yaw Fui
Department of Surgery
Singapore General Hospital
Outram Road
Singapore 169608
Tel: +65 6321 4051 Fax: + 65 6220 9323
Email: alexander.chung.y.f@sgh.com.sg

National Cancer Center
Dr. Donald Poon Yew Hee
Department of Medical Oncology
National Cancer Centre
11 Hospital Drive
Singapore 169610
Tel: +65 6436 8000 Fax: +65 6324 0875
Email: dmopyh@nccs.com.sg</td></tr>
<tr><td>6</td><td>12</td><td>1.1 Contact Details
Principal Investigator</td><td>Assoc. Prof. Pierce Chow Kah Hoe
Duke-NUS Graduate Medical School Singapore
2 Jalan Bukit Merah
Singapore 169547
Tel: +65 6516 7666 Fax: +65 6224 6242
Email: pierce.chow@gms.edu.sg</td><td>Assoc. Prof. Pierce Chow Kah Hoe
Department of General Surgery
Singapore General Hospital,
Outram Road
Singapore 169608
Tel: +65 63214051 Fax: +65 62209323
Email: gsupc@singnet.com.sg</td></tr>
<tr><td>7</td><td>12</td><td>1.1 Contact Details
Study Radiologists</td><td>The original text do not have this information</td><td>Dr Albert Low
Department of Diagnostic Radiology
Singapore General Hospital
Outram Road
Singapore 169608
Tel: +65 6326 5029 Fax: + 65 6326 5161
Email: albert.low.sc@sgh.com.sg</td></tr>
</table>

8	12	1.1 Contact Details<br>Study Interventional Radiologists	The original text do not have this information	Assoc. Prof. Tay Kiang Hiong<br>Department of Diagnostic Radiology<br>Singapore General Hospital<br>Outram Road<br>Singapore 169608<br>Tel: +65 6326 5029 Fax: + 65 6326 5161<br>Email: tay.kiang.hiong@sgh.com.sg<br><br>Dr. Richard Lo Hoau Gong<br>Department of Diagnostic Radiology<br>Singapore General Hospital<br>Outram Road<br>Singapore 169608<br>Tel: +65 6321 4409 Fax: + 65 6224 1407<br>Email: richard.lo.h.g@sgh.com.sg
9	13	1.1 Contact Details<br>Study Medical Oncologist	The original text do not have this information	Dr. Donald Poon Yew Hee<br>Department of Medical Oncology<br>National Cancer Centre<br>11 Hospital Drive<br>Singapore 169610<br>Tel: +65 6436 8000 Fax: +65 6324 0875<br>Email: dmopyh@nccs.com.sg
10	13	1.1 Contact Details<br>Study Statisticians	Dr Chen Yu Ming<br>Singapore Clinical Research Institute<br>31 Biopolis Way<br>#02-01 Nanos<br>Singapore 138669<br>Tel: +65 6508 80331 Fax: +65 6508 8317<br>Email: yuming.chen@scri.edu.sg<br><br>Assoc. Prof. Tan Say Beng<br>Singapore Clinical Research Institute<br>31 Biopolis Way<br>#02-01 Nanos<br>Singapore 138669<br>Tel: +65 6508 8303 Fax: +65 6508 8317<br>Email: saybeng@cteru.com.sg	Ms. Zhang Xiaoe<br>Singapore Clinical Research Institute<br>31 Biopolis Way<br>#02-01 Nanos<br>Singapore 138669<br>Tel: +65 6508 80328 Fax: +65 6508 8317<br>Email: Xiaoe.zhang@scri.edu.sg<br><br>Assoc. Prof. Tan Say Beng<br>Singapore Clinical Research Institute<br>31 Biopolis Way<br>#02-01 Nanos<br>Singapore 138669<br>Tel: +65 6508 8303 Fax: +65 6508 8317<br>Email: saybeng.tan@scri.edu.sg

11	14	1.1 Contact Details<br>Trial Manager	**Data Manager**<br>Ms. Priscilla Li Choi Nar<br>Singapore Clinical Research Institute<br>31 Biopolis Way<br>#02-01 Nanos<br>Singapore 138669<br>Tel: +65 6508 8357 Fax: +65 6508 8317<br>Email: priscilla.li@scri.edu.sg	**Trial Manager**<br>Ms. Priscilla Li Choi Nar<br>Singapore Clinical Research Institute<br>31 Biopolis Way<br>#02-01 Nanos<br>Singapore 138669<br>Tel: +65 6508 8357 Fax: +65 6508 8317<br>Email: priscilla.li@scri.edu.sg
12	14	1.1 Contact Details<br>Overseas Site Investigators	The original text do not have this information	Korea Site Investigators<br>Prof Han Ho Seong<br>Seoul National University Bundang Hospital<br>300 Gumi-dong, Bundang-gu, Seongnam-si, Gyeonggi-do 463-707, Korea<br>Tel: +031-787-4055 Fax: +031-787-4055<br>Email: hanhs@snubh.org<br><br>Malaysia Site Investigators<br>Datuk (Dr) Harjit Singh<br>Department of Hepato-Pancreato-Biliary Surgery<br>Hospital Selayang<br>Lebuhraya Selayang-Kepong<br>Batu Caves 68100 Selangor, Malaysia<br>Tel: +603-6120 2122 Fax: +603-6120 7564<br>Email: harjit@selayanghospital.gov.my<br><br>Myanmar Site Investigators<br>Prof Khin Maung Win<br>Yangon GI and Liver Center<br>No. 191 – 193<br>30th Street, Upper Block<br>Pabedan Township<br>Yangon, Myanmar<br>Tel: +95-1-256128 Fax: +95-1-222965<br>Email: 30thstreetclinic@mptmail.net.mm

13	15	1.2 Independent Data and Safety Monitoring Committee	The original text do not have this section	An independent Data and Safety Monitoring Committee (DSMC) comprising an international panel of senior clinicians and experienced trialist with known expertise in the management of hepatocellular carcinoma will be appointed to advice the Steering Committee on safety and ethical aspects of the trial.<br><br>DSMC Chair<br>Prof Joseph Lau<br>Dept of Surgery<br>Prince of Wales Hospital<br>Chinese University of Hong Kong<br>Shatin, New Territories<br>Hong Kong<br>Tel: +852 2632 2415<br>Email: josephlau@surgery.cuhk.edu.hk<br><br>DSMC Member<br>Prof Thomas Leung<br>Hong Kong Sanatorium &<br>Hospital Oncology Centre<br>Comprehensive Oncology Centre<br>4/F Central Block,<br>2 Village Road,<br>Happy Valley, Hong Kong<br>Tel: +852 2835 8877<br>Email: thomaswtleung@hksh.com<br><br>DSMC Member<br>Prof Bruno Sangro<br>Dept of Internal Medicine<br>Clinica Universitaria de Navarra<br>Avda. Pio XII 36<br>31192 Pamplona<br>Spain<br>Tel: +349 4829 6637<br>Email: bsangro@unav.es

14	34	7.1.1 Complications and Toxic Effects of SIR-Spheres	The most common potential serious complications result from either (i) inadvertent administration of SIR-Spheres into the gastrointestinal tract resulting in gastritis/duodenitis or (ii) radiation induced liver disease resulting from a radiation overdose to the normal liver parenchyma. The incidence of gastritis/duodenitis can be reduced by meticulous attention to the administration procedure so as to ensure that there is a minimal chance of SIR-Spheres entering the numerous small arteries supplying the gastrointestinal tract (Salem 2006; Liu 2005). Radiation induced liver disease is largely, but not totally, preventable by using appropriate SIRT doses and making allowances for dose reduction when there is increased risk of causing radiation damage such as in pre-existing liver damage, poor liver reserve or small volume tumour mass in the liver. The reported incidence of gastritis/duodenitis is <10%, while the reported rate of radiation induced liver disease is < 1%.	The most common potential serious complications result from either (i) inadvertent administration of SIR-Spheres into the gastrointestinal tract resulting in gastritis/duodenitis or (ii) radiation induced liver disease resulting from a radiation overdose to the normal liver parenchyma. The incidence of gastritis/duodenitis can be reduced by meticulous attention to the administration procedure so as to ensure that there is a minimal chance of SIR-Spheres entering the numerous small arteries supplying the gastrointestinal tract (Salem 2006; Liu 2005). Radiation induced liver disease is largely, but not totally, preventable by using appropriate SIRT doses and making allowances for dose reduction when there is increased risk of causing radiation damage such as in pre-existing liver damage, poor liver reserve or small volume tumour mass in the liver. The reported incidence of gastritis/duodenitis is <10%, while the reported rate of radiation induced liver disease is < 1%; radiation induced liver disease may lead to death.
15	34	7.1.1 Complications and Toxic Effects of SIR-Spheres	Previously reported radiation pneumonitis has not been observed where appropriate pre-treatment workup and dose reductions are followed.	The incidence of radiation pneumonitis (inflammation of the lungs due to radiation) is expected to be low where appropriate pre-treatment workup and dose reductions are followed. The risk of radiation pneumonitis nevertheless exists and has been reported.
16	39	9.1 Patient Eligibility	b) Space occupying lesion of the liver demonstrated by ultrasound, CT scan (non-dynamic) or MRI (non-dynamic) and:<br>• Serum alpha-feto protein level of at least 400 mcg/L done at anytime or	b) Space occupying lesion of the liver demonstrated by ultrasound, CT scan (dynamic or non-dynamic) or MRI (dynamic or non-dynamic) and:<br>• Serum alpha-feto protein level of at least 400 mcg/L done at anytime or

**17**	**42**	**9.3.2 Exclusion Criteria Specific to This Investigational Study**	**The original text do not have this criteria**	**d) Subjects with inferior vena cava (IVC) tumour thrombus or invasion.**
**18**	**57**	**12.2 Serial Study Measurements**	**The original text do not have this information**	**ECG and MUGA scan (recommended).**
**19**	**57**	**12.2 Serial Study Measurements *(Table)***	**ECG and MUGA scan**	**ECG and MUGA scan *(recommended)***
**20**	**58**	**Table 12.2 Study Treatment Plan**	**ECG and MUGA scan**	**ECG and MUGA scan *(recommended)***
**21**	**59**	**Notes to Study Plan**	**a) ECG and MUGA scan to be performed at Baseline, every 12 weeks during protocol therapy of Sorafenib Treatment and End of Study (as determined by physician).**	**a) ECG and MUGA scan to be performed at Baseline, every 12 weeks during protocol therapy of Sorafenib Treatment and End of Study (recommended).**
**22**	**61**	**14.2.1 RECIST Guidelines**	**All other lesions (or sites of disease) should be identified as non-target lesions and should also be recorded at baseline. Measurements of these lesions are not required, but the presence or absence of each should be noted throughout follow-up.**	**All other lesions (or sites of disease) should be identified as non-target lesions and should also be recorded at baseline. Non-target lesions include measurable lesions that exceed the maximum numbers per organ or total of all involved organs as well as non-measurable lesions. Measurements of these lesions are not required, but the presence or absence of each should be noted throughout follow-up.**<br><br>**As for local response in hepatic lesions, RECIST will be used. The data on which lesions were treated with SIR-SPHEREs will be recorded, so that the local responses between treated and untreated lesions may be differentiated and allow reasonable analysis of local activity subsequently.**

23	62	14.2.2 Response Criteria	Progressive Disease (PD): At least a 20% increase in the sum of the longest diameter of the target lesions, taking as a reference the smallest sum longest diameter recorded since treatment started or the appearance of new lesions.	Progressive Disease (PD): At least a 20% increase in the sum of the longest diameter of the target lesions, taking as a reference the smallest sum longest diameter recorded since treatment started or the appearance of one or more new lesions.
24	62	14.2.3 Exploratory Response Criteria of Local Tumor Ablation	The original text do not have this section	A percentage tumour necrosis as response evaluation criteria with the following categories will be used as secondary assessment.<br><br>Complete response (CR): 100% tumour necrosis in the post-treatment scan<br><br>Partial response (PR): 30-99% increase in the percentage tumour necrosis<br><br>Insufficient response (IR): less than 30% increase in the percentage tumour necrosis<br><br>Notes:<br><br>1) Necrosis is defined by an area of tumour that enhances by no more than 10 Hounsfield units in every postcontrast phase of the CT scan.<br><br>2) Percentage increase of tumour necrosis is defined as the increase in the extent of tumour necrosis from the baseline scan to the post-treatment scan.<br><br>3) The extent or percentage of tumour necrosis is determined as the area of necrosis divided by total tumour area X 100% in an axial section through the tumour that shows the largest area of necrosis.

**25**	**67**	**17.1.1 Adverse event (AE) (ISO 14155-1:2003)**	**Adverse Events will be recorded from the date of signature of the informed consent up to 30 days after the last dose of <u>chemotherapy</u> is administered. If the AE is a treatment-related toxicity, follow-up will continue until resolution.**	**Adverse Events will be recorded from the date of signature of the informed consent up to 30 days after the last dose of <u>protocol therapy</u> is administered. If the AE is a treatment-related toxicity, follow-up will continue until resolution.**
**26**	**68**	**17.2 Reporting**	**SAE will be recorded from the date of signature of the informed consent up to 30 days after the last dose of <u>chemotherapy</u> is administered.**	**SAE will be recorded from the date of signature of the informed consent up to 30 days after the last dose of <u>protocol therapy</u> is administered.**

SingHealth

Tel: (65) 6225 0488
Fax: (65) 6557 2138
Singapore Health Services Pte Ltd
11 Third Hospital Avenue
#07-00 SNEC Building
Singapore 168751
www.singhealth.com.sg

Reg No 200002698Z

SHS IRB Ref No: 2008/422/B

4 May 2009

A/Prof Pierce Chow
Department of General Surgery
Singapore General Surgery

Dear A/Prof Chow

APPLICATION TO CONDUCT RESEARCH IN SINGHEALTH

Protocol No: AHCC05
Protocol Title: Phase I/II Study of SIR-SPHERES® plus Sorafenib (Chemo-Radiotherapy) as First Line Treatment in Patients with Non-Resectable Primary Hepatocellular Carcinoma
Protocol Amendment version 8 dated 28 Mar 2009

Thank you for your email clarification dated 30 Apr 2009.

We are pleased to inform you that the SingHealth Centralised Institutional Review Board B has approved the protocol amendment stated above on 16 Apr 2009.

The IRB has also no objection to the nomination of Dr Alexander Chung as the SGH site PI, and the addition of Dr Albert Low Su Chong as a study diagnostic radiologist for the above stated study.

SHS CIRB B Review Composition:

Name	IRB Membership	Designation, Institution	Gender
Vijay Kumar Sethi	Chairman	Senior Consultant, Department of Radiation Oncology, National Cancer Centre	Male
Lim Soon Thye	Member	Senior Consultant, Department of Medical Oncology, National Cancer Centre	Male
Toh Chee Keong	Member	Consultant, Department of Medical Oncology, National Cancer Centre	Male
Serene Lim	Member	Senior Legal Counsel, Singapore Health Services	Female
Edward Poon	Member	Nursing Director, Dover Park Hospice	Male
Winnie Lee	Alternate Member	Senior Clinical Pharmacist, Department of Pharmacy, Singapore General Hospital	Female
Tan Peng Chin	Alternate Member	Managing Director, Tan Peng Chin LLC	Male

Yours sincerely

Dr Vijay Sethi
Chairman, Institutional Review Board B
SingHealth

SingHealth

Tel: (65) 6225 0488
Fax: (65) 6557 2138
Singapore Health Services Pte Ltd
11 Third Hospital Avenue
#07-00 SNEC Building
Singapore 168751
www.singhealth.com.sg
Reg No 200002698Z

Cc Dr Alexander Chung (SGH site PI)
Dept of General Surgery, SGH

Cc Head, Department of General Surgery, SGH

Cc Institutional Representative, SGH

Cc Dr Donald Poon (NCC site PI)
Dept of Medical Oncology, NCC

Cc Head, Department of Medical Oncology, NCC

Cc Institutional Representative, NCC

Please quote SHS IRB Ref No: 2008/422/B in future correspondences with the Board.

Singapore
General Hospital
SingHealth

Tel: (65) 6222 3322
Fax: (65) 6224 9221
Singapore General Hospital
Outram Road
Singapore 169608
www.sgh.com.sg
Reg No 198703907 Z

27 March 2009

Dr Aw Swee Eng
Chairman IRB
Bowyer Block A Level 3
Singapore General Hospital
Singapore 169608

IRB Ref: #305 / 2007

Dear Dr Aw,

Protocol Amendment, Change of Principal Investigator and Addition of Study Radiologist for IRB Ref: #305/2007 – Phase I/ II Study of SIR-Spheres plus Sorafenib as First Line Treatment in Patients with Non-Resectable Primary Hepatocellular Carcinoma

I would like to notify the board that I would like to nominate Dr Alexander Chung as the Site Principal Investigator for Singapore General Hospital as I will be the International Study Principal Investigator for the above stated study.

Also, I would like to include 1 additional Study Diagnostic Radiologist: Dr Albert Low Su Chong for the above stated study.

Please find enclosed the following documents for your review and approval:

1. 1 original and 10 copies of Amended Protocol Version 8.0, dated 27 Mar 2009 with tracked changes
2. 1 original and 10 copies of Summary of Protocol Amendments Version 8.0, dated 27 Mar 2009
3. Latest, signed and dated CV (of Dr Albert Low)
4. Updated CITI certificate (of Dr Albert Low)

Please be informed that there are no amendments made to the Patient Information Sheet and Informed Consent Form.

Thank you and I look forward to your favorable reply.

Best Regards

A/PROF PIERCE CHOW KAH HOE
PRINCIPAL INVESTIGATOR

Members of the SingHealth Group
Changi General Hospital • KK Women's and Children's Hospital • Singapore General Hospital
National Cancer Centre Singapore • National Dental Centre • National Heart Centre • National Neuroscience Institute
Singapore National Eye Centre • SingHealth Polyclinics

Accredited by
Joint Commission
INTERNATIONAL

SUMMARY FOR PROTOCOL AMENDMENT

Protocol Title: Phase I/II Study of SIR-Spheres Plus Sorafenib as First Line Treatment in Patients with Non-Resectable Primary Hepatocellular Carcinoma

Approved Version: Version 6.1, dated 20 Oct 2008
Amended Version: Version 7.0, dated 11 Dec 2008

S/N	PAGE NO. INVOLVED	DESCRIPTION	AMENDED FROM	AMENDED TO
			VERSION 6.1 DATED 20 OCT 2008	VERSION 7.0 DATED 11 DEC 2008
1	1	Cover Page, Title	Phase I/II Study of SIR-Spheres Plus Sorafenib (Chemo-Radiotherapy) as First Line Treatment in Patients with Non-Resectable Primary Hepatocellular Carcinoma	Phase I/II Study of SIR-Spheres Plus Sorafenib as First Line Treatment in Patients with Non-Resectable Primary Hepatocellular Carcinoma
2	1	Title	October 2008	December 2008
3	2	Title	"Phase I/II Study of SIR-Spheres Plus Sorafenib (Chemo-Radiotherapy) as First Line Treatment in Patients with Non-Resectable Primary Hepatocellular Carcinoma"	"Phase I/II Study of SIR-Spheres Plus Sorafenib as First Line Treatment in Patients with Non-Resectable Primary Hepatocellular Carcinoma"
4	2	Co-Investigator Details	Assoc. Prof Tan Say Beng<br>Clinical Trials and Epidemiology Research Unit<br>226 Outram Road<br>Blk A #03-02<br>Singapore 169039<br>Tel: +65 6325 7060 Fax: +65 6324 2700<br>Email: saybeng@cteru.com.sg	Assoc. Prof Tan Say Beng<br>Singapore Clinical Research Institute<br>31 Biopolis Way<br>#02-01 Nanos<br>Singapore 138669<br>Tel: +65 6508 8303 Fax: +65 6508 8317<br>Email: saybeng.tan@scri.edu.sg
5	3b	Page 3b, Steering Committee	The original text do not have Page 3b	Page 3b is added with addition of the Steering Committee Members:<br><br>Assoc. Prof. Pierce Chow Kah Hoe<br>Duke-NUS Graduate Medical School Singapore<br>2 Jalan Bukit Merah<br>Singapore 169547<br>Tel: +65 6516 7666 Fax: +65 6224 6242<br>Email: pierce.chow@gms.edu.sg

5	3b	Page 3b, Steering Committee	The original text do not have Page 3b	**Prof. Soo Khee Chee**<br>**11 Hospital Drive**<br>**National Cancer Centre**<br>**Singapore 169610**<br>**Tel: +65 6436 8205 Fax: +65 6220 7759**<br>**Email: admskc@nccs.com.sg**<br><br>**Assoc. Prof. Tan Say Beng**<br>**Singapore Clinical Research Institute**<br>**31 Biopolis Way**<br>**#02-01 Nanos**<br>**Singapore 138669**<br>**Tel: +65 6508 8303 Fax: +65 65088317**<br>**Email: saybeng.tan@scri.edu.sg**<br><br>**Dr. Anthony Goh Soon Whatt**<br>**Department of Nuclear Medicine**<br>**Singapore General Hospital**<br>**Outram Road**<br>**Singapore 169608**<br>**Tel: +65 6321 4649 Fax: + 65 6224 0938**<br>**Email: anthony.goh.s.w@sgh.com.sg**<br><br>**Dr. Lai Hee Kit**<br>**Department of Nuclear Medicine**<br>**Singapore General Hospital**<br>**Outram Road**<br>**Singapore 169608**<br>**Tel: +65 6321 4878 Fax: +65 62240938**<br>**Email: lai.hee.kit@singhealth.com.sg**<br><br>**Assoc. Prof. Tay Kiang Hiong**<br>**Department of Diagnostic Radiology**<br>**Singapore General Hospital**<br>**Outram Road**<br>**Singapore 169608**<br>**Tel: +65 6326 5029 Fax: + 65 6326 5161**<br>**Email: tay.kiang.hiong@sgh.com.sg**

5	3b	Page 3b, Steering Committee	The original text do not have Page 3b	Dr. Richard Lo Hoau Gong<br>Department of Diagnostic Radiology<br>Singapore General Hospital<br>Outram Road<br>Singapore 169608<br>Tel: +65 6321 4409 Fax: + 65 6224 1407<br>Email: richard.lo.h.g@sgh.com.sg<br><br>Dr. Donald Poon Yew Hee<br>Department of Medical Oncology<br>National Cancer Centre<br>11 Hospital Drive<br>Singapore 169610<br>Tel: +65 6436 8000 Fax: +65 6324 0875<br>Email: dmopyh@nccs.com.sg<br><br>Dr. Choo Su Pin<br>Department of Medical Oncology<br>National Cancer Centre<br>11 Hospital Drive<br>Singapore 169610<br>Tel: +65 6436 8000 Fax: + 65 6227 2759<br>Email: choosupin@nccs.com.sg<br><br>Lim Teong Guan<br>Singapore General Hospital<br>Outram Road<br>Singapore 169608<br>Tel: +65 6321 4110 Fax: +65 6321 1335<br>Email: lim.teong.guan@sgh.com.sg
6	7	Section 1.1 Contact Details, Study Statiscian	Tel: +65 6325 7063 Fax: +65 6324 2700<br>yuming@cteru.com.sg	Tel: +65 6508 8331 Fax: +65 6508 8317<br>yuming.chen@scri.edu.sg
7	7	Section 1.1 Contact Details, Study Statiscian	Assoc. Prof Tan Say Beng<br>Clinical Trials and Epidemiology Research Unit<br>226 Outram Road<br>Blk A #03-02<br>Singapore 169039<br>Tel: +65 6325 7060 Fax: +65 6324 2700<br>Email: saybeng@cteru.com.sg	Assoc. Prof Tan Say Beng<br>Singapore Clinical Research Institute<br>31 Biopolis Way<br>#02-01 Nanos<br>Singapore 138669<br>Tel: +65 6508 8303 Fax: +65 6508 8317<br>Email: saybeng.tan@scri.edu.sg

8	8	Section 1.1 Contact Details, Study Monitor	Tel: +65 6325 7097 Fax: +65 6324 2700<br>Email: yeechien@cteru.com.sg	Tel: +65 6508 8354 Fax: +65 6508 8317<br>Email: yeechien.chua@scri.edu.sg
9	8	Section 1.1 Contact Details, Data Manager	Tel: +65 6325 7081 Fax: +65 6324 2700<br>Email: priscilla@cteru.com.sg	Tel: +65 6508 8357 Fax: +65 6508 8317<br>Email: priscilla.li@scri.edu.sg
10	9	Overall Synopsis Of The Clinical Investigation, Section 2	"This Phase I/II trial will evaluate the safety and activity of chemo-radiotherapy comprising a regimen of Sorafenib chemotherapy plus SIR-Spheres yttrium-90 microspheres (chemo-radiotherapy, also known as "chemo-SIRT"), for first-line treatment of patients with primary hepatocellular carcinoma (HCC) in whom surgical resection is not feasible."	"This Phase I/II trial will evaluate the safety and activity of a regimen comprising Sorafenib chemotherapy plus SIR-Spheres yttrium-90 microspheres, for first-line treatment of patients with primary hepatocellular carcinoma (HCC) in whom surgical resection is not feasible."
11	10	Approval And Agreement, Section 3	I have read and understand the requirements of this study protocol, "Phase I/II study of SIR-Spheres plus Sorafenib (chemo-radiotherapy) as first line treatment in patients with non-resectable primary hepatocellular carcinoma".	I have read and understand the requirements of this study protocol, "Phase I/II study of SIR-Spheres plus Sorafenib as first line treatment in patients with non-resectable primary hepatocellular carcinoma".
12	32	Section 9, Design Of The Clinical Study, Line 4	"The study will recruit a maximum of 31 patients."	"The study will recruit a maximum of 35 patients."
13	32	Section 9, Design Of The Clinical Study, Study Design Diagram	"PAHSE II"	"PHASE II"
14	35	Section 9.3.2, Exclusion Criteria Specific to This Investigational Study, Exclusion Criteria (a)	"Subjects who have had previous hepatic artery directed therapy within the previous 3 months."	"Subjects who have had any previous hepatic artery directed therapy."

15	35	Section 9.3.2, Exclusion Criteria Specific to This Investigational Study, Exclusion Criteria (b)	"Subjects who have had intravenous chemotherapy within the previous 4 weeks or those who have not recovered from adverse events due to agents administered more than 6 weeks previously."	"Subjects who have had prior chemotherapy or other medical agents used to treat Hepatocellular carcinoma."
16	35	Section 9.3.2, Exclusion Criteria Specific to This Investigational Study, Exclusion Criteria (c)	"Prior external hepatic radiation therapy for HCC, more than two prior systemic chemotherapy regimes for HCC or any other concomitant therapy for HCC or any investigational agent planned while on this protocol."	"Prior external hepatic radiation therapy for HCC, or any other concomitant therapy for HCC or any investigational agent planned while on this protocol."
17	50	Section 12.2, Serial Study Measurements, Serial Study Measurement Table, Point 5	The original text do not have this point	ECG and MUGA scan: Baseline, 12 weekly during protocol therapy of Sorafenib Treatment thereafter and End of Study (as appropriate)
18	51	Section 12.2, Serial Study Measurements, Table 12.2 Study Treatment Plan	The original text do not have this point	Addition of ECG and MUGA scan in plan, Point 14
19	52	Section 12.2, Serial Study Measurements, Notes to Study Plan	The original text do not have this point	ECG and MUGA scan to be performed at Baseline, every 12 weeks during protocol therapy of Sorafenib Treatment and End of Study (as determined by physician)
20	56	Study Design And Sample Size, Section 15.1, First Paragraph	"This Phase I/II study will evaluate the safety and initial effectiveness of combining Sorafenib therapy with SIR-Spheres (chemo-radiotherapy) for the treatment of patients with primary HCC in whom surgical resection is not feasible."	"This Phase I/II study will evaluate the safety and initial effectiveness of combining Sorafenib therapy with SIR-Spheres for the treatment of patients with primary HCC in whom surgical resection is not feasible."
21	92	Informed Consent Form, Appendix 11, Study Title	"This Phase I/II study will evaluate the safety and initial effectiveness of combining Sorafenib therapy with SIR-Spheres (chemo-radiotherapy) for the treatment of patients with primary HCC in whom surgical resection is not feasible."	"This Phase I/II study will evaluate the safety and initial effectiveness of combining Sorafenib therapy with SIR-Spheres for the treatment of patients with primary HCC in whom surgical resection is not feasible."

**22**	**93**	**Patient Information Sheet, Appendix 12, Study Title**	**“This Phase I/II study will evaluate the safety and initial effectiveness of combining Sorafenib therapy with SIR-Spheres (chemo-radiotherapy) for the treatment of patients with primary HCC in whom surgical resection is not feasible.”**	**“This Phase I/II study will evaluate the safety and initial effectiveness of combining Sorafenib therapy with SIR-Spheres for the treatment of patients with primary HCC in whom surgical resection is not feasible.”**

Singapore General Hospital
SingHealth

Tel: (65) 6222 3322
Fax: (65) 6224 9221
Singapore General Hospital
Outram Road
Singapore 169608
www.sgh.com.sg

SINGAPORE GENERAL HOSPITAL<br>INSTITUTIONAL REVIEW BOARD<br>Outram Road<br>Singapore 169608<br>Tel: 6321 3518<br>Fax: 6222 1720	
**IRB APPROVAL FORM**<br>**(Amendments)**<br>**A: DETAILS OF STUDY**	
**IRB Ref. Number:**	305/2007
**Title Of Study:**	Phase I/ II Study of Sir-Spheres® Plus Sorafenib (Chemo-radiotherapy) as first line treatment in patients with non-resectable primary hepatocellular carcinoma. Protocol AHCC05
**Principal Investigator:**	A/Prof Pierce Chow, Senior Consultant, Dept of General Surgery
**Sponsor (if any):**	-
**B: DOCUMENTS REVIEWED:**<br>**Document:**	
▪ Amended Protocol Version 7.0 – 11 Dec 08<br>▪ Amended Patient Information Sheet & Informed Consent Form Version 5 – 11 Dec 08	
**C: The IRB has reviewed the above-mentioned documents, and has approved the proposed amendments.**<br><br>The IRB operates in accordance with the ICH/ Singapore Guideline for Good Clinical Practices, and with the applicable regulatory requirement(s).	
**IRB Approval Date:**	22 December 2008
**D: THE FOLLOWING ARE TO BE OBSERVED UPON IRB APPROVAL**	
1. No subject should be admitted to the trial before the Health Sciences Authority issues the Clinical Trial Certificate (applicable only for drug trials). A copy of the Clinical Trial Certificate should be submitted to the IRB.<br><br>2 The Principal Investigator should ensure that this study is conducted in compliance with the Singapore Guideline for Good Clinical Practice, the ethical guidelines of which are applicable to all studies to be carried out, and to ensure that the study is carried out in accordance to the guidelines and the submitted protocol. The Principal Investigator should meet with his collaborator(s) regularly to assess the progress of the study, and be familiar and comply with all applicable research policies in the Institution.<br><br>3. This approval is valid for till **<u>4 February 2009</u>**.	

4. No deviation from, or changes of, the protocol should be initiated without prior written IRB approval of an appropriate amendment, except when necessary to eliminate immediate hazards to the subjects or when the change(s) involve(s) only logistical or administrative aspects of the trial (e.g. change of monitor(s), telephone number(s).

5. Only the approved Patient Information Sheet and Consent Form should be used. It must be signed by each subject prior to enrolling in the study and initiation of any protocol procedures. Three copies of the Informed Consent Form should be signed and dated. Each subject or the subject's legally accepted representative should be given a copy of the signed consent form. The remaining 2 copies should be kept by the PI and in the case report respectively.

6. The Principal Investigator should promptly inform the IRB of:
   a. deviations from or changes of the protocol made to eliminate immediate hazards to the trial subjects.
   b. changes increasing the risk to subjects and/or affecting significantly the conduct of the trial.
   c. all adverse events within the stipulated time lines.
   d. new information that may affect adversely the safety of the subjects or the conduct of the trial.
   e. completion of the study.

7. Study Status Report should be submitted to the IRB for the following:
   a. Annual review: Status of the study should be reported to the IRB at least annually using the Study Status Report.
   b. Study renewal: the Study Status Report is to be submitted one month prior to expiry of the approval period.
   c. Study completion or termination: the Study Status Reports is to be submitted within three months of study completion or termination.

E: COMMENTS / FEEDBACK FROM IRB MEMBERS, IF ANY

NA

F: SIGNATURE OF INSTITUTIONAL REVIEW BOARD CHAIRPERSON

Dr Aw Swee Eng	[signature]
Name	Signature

SUMMARY FOR PROTOCOL AMENDMENT

Protocol Title: Phase I/II Study of SIR-Spheres Plus Sorafenib (Chemo-Radiotherapy) as First Line Treatment in Patients with Non-Resectable Primary Hepatocellular Carcinoma

Approved Version: Version 6, dated 8 Sep 2008
Amended Version: Version 6.1, dated 20 Oct 2008

S/N	PAGE NO. INVOLVED	DESCRIPTION	AMENDED FROM	AMENDED TO
			VERSION 6 DATED 8 SEP 2008	VERSION 6.1 DATED 20 OCT 2008
1	6	Content Page No. for Patient Information Sheet Index	Introduction 98<br>Purpose of Study 98<br>Who can be in the study? 98<br>Patient's Responsibility 98<br>Possible Side Effects 98<br>Anticipated expense 98<br>Confidentiality 98<br>Contact person 98<br>Voluntary participation 98	Introduction 93<br>Purpose of Study 93<br>Who can be in the study? 94<br>Patient's Responsibility 94<br>Possible Side Effects 95<br>Anticipated expense 96<br>Confidentiality 97<br>Contact person 97<br>Voluntary participation 98
2	7	Section 1.1 Contact Details, Study Statistician	Dr. Chen Yu Ming<br>Singapore Clinical Research Institute<br>226 Outram Road<br>Blk A #03-02<br>Singapore 169039	Dr. Chen Yu Ming<br>Singapore Clinical Research Institute<br>31 Biopolis Way<br>#02-01 Nanos<br>Singapore 138669
3	8	Section 1.1 Contact Details, Study Monitor	Ms. Chua Yee Chien<br>Singapore Clinical Research Institute<br>226 Outram Road<br>Blk A #03-02<br>Singapore 169039	Ms. Chua Yee Chien<br>Singapore Clinical Research Institute<br>31 Biopolis Way<br>#02-01 Nanos<br>Singapore 138669
4	8	Section 1.1 Contact Details, Data Manager	Ms. Priscilla Li Choi Nar<br>Singapore Clinical Research Institute<br>226 Outram Road<br>Blk A #03-02<br>Singapore 169039	Ms. Priscilla Li Choi Nar<br>Singapore Clinical Research Institute<br>31 Biopolis Way<br>#02-01 Nanos<br>Singapore 138669

5	34	Section 9.2 Inclusion Criteria, (g)	ECOG performance status 0 – 1.	ECOG performance status 0 – 1 (see Appendix 1).
6	59	Section 17.1.1 Adverse Event, Line 6	“Adverse Events will be recorded from the date of signature of the informed consent up to 28 days after the last dose of chemotherapy is administered.”	“Adverse Events will be recorded from the date of signature of the informed consent up to 30 days after the last dose of chemotherapy is administered.”
7	59	Section 17.1.1 Adverse Event, Line 7	“If the AE is a SIRT-related toxicity, …”	“If the AE is a treatment-related toxicity, …”
8	59	Section 17.2 Reporting	Blank	"SAE will be recorded from the date of signature of the informed consent up to 30 days after the last dose of chemotherapy is administered."
9	61	Section 18 Ethical Considerations, Line 2	“… World Medical Association Declaration of Helsinki (see Appendix 11)”	“… World Medical Association Declaration of Helsinki (see Appendix 10)”
10	61	Section 18.1 Informed Consent, Last Line	“A copy of the Informed Consent Document can be found in Appendix 13.”	“A copy of the Informed Consent Document can be found in Appendix 11.”
11	62	Section 19 Publication Policy, Line 3	“As an investigator-initiated study, the manufacturer (Sirtex Medical) does not have ownership of the original data or influence over what data can be submitted for publication and/or presentation at meetings.”	“Consistent with this being an investigator-initiated study, the manufacturer/sponsor (Sirtex Medical/ Bayer Schering Pharma) does not have the ownership of the original data or influence over what data can be submitted for publication and/or presentation at meetings.”
12	82 - 99	Missing Page No. at Footer	Blank	Page No. 82 to 99

Singapore
General Hospital
SingHealth

Tel: (65) 6222 3322
Fax: (65) 6224 9221
Singapore General Hospital
Outram Road
Singapore 169608
www.sgh.com.sg

SINGAPORE GENERAL HOSPITAL
INSTITUTIONAL REVIEW BOARD
Outram Road
Singapore 169608
Tel: 6321 3518
Fax: 6222 1720

IRB APPROVAL FORM
(Amendments)

A: DETAILS OF STUDY

**IRB Ref. Number:**	305/2007
**Title Of Study:**	Phase I/ II Study of Sir-Spheres® Plus Sorafenib (Chemo-radiotherapy) as first line treatment in patients with non-resectable primary hepatocellular carcinoma. Protocol AHCC05
**Principal Investigator:**	A/Prof Pierce Chow, Senior Consultant, Dept of General Surgery
**Sponsor (if any):**	-

B: DOCUMENTS REVIEWED:

Document:

- Protocol Amendment Ver 6.1 – 20 Oct 08

C: The IRB has reviewed the above-mentioned document, and has approved the proposed amendments.

The IRB operates in accordance with the ICH/ Singapore Guideline for Good Clinical Practices, and with the applicable regulatory requirement(s).

IRB Approval Date: 31 October 2008

D: THE FOLLOWING ARE TO BE OBSERVED UPON IRB APPROVAL

1. No subject should be admitted to the trial before the Health Sciences Authority issues the Clinical Trial Certificate (applicable only for drug trials). A copy of the Clinical Trial Certificate should be submitted to the IRB.

2 The Principal Investigator should ensure that this study is conducted in compliance with the Singapore Guideline for Good Clinical Practice, the ethical guidelines of which are applicable to all studies to be carried out, and to ensure that the study is carried out in accordance to the guidelines and the submitted protocol. The Principal Investigator should meet with his collaborator(s) regularly to assess the progress of the study, and be familiar and comply with all applicable research policies in the Institution.

3. This approval is valid for till **4 February 2009**.

4. No deviation from, or changes of, the protocol should be initiated without prior written

IRB approval of an appropriate amendment, except when necessary to eliminate immediate hazards to the subjects or when the change(s) involve(s) only logistical or administrative aspects of the trial (e.g. change of monitor(s), telephone number(s).

5. Only the approved Patient Information Sheet and Consent Form should be used. It must be signed by each subject prior to enrolling in the study and initiation of any protocol procedures. Three copies of the Informed Consent Form should be signed and dated. Each subject or the subject's legally accepted representative should be given a copy of the signed consent form. The remaining 2 copies should be kept by the PI and in the case report respectively.

6. The Principal Investigator should promptly inform the IRB of:
   a. deviations from or changes of the protocol made to eliminate immediate hazards to the trial subjects.
   b. changes increasing the risk to subjects and/or affecting significantly the conduct of the trial.
   c. all adverse events within the stipulated time lines.
   d. new information that may affect adversely the safety of the subjects or the conduct of the trial.
   e. completion of the study.

7. Study Status Report should be submitted to the IRB for the following:
   a. Annual review: Status of the study should be reported to the IRB at least annually using the Study Status Report.
   b. Study renewal: the Study Status Report is to be submitted one month prior to expiry of the approval period.
   c. Study completion or termination: the Study Status Reports is to be submitted within three months of study completion or termination.

E: COMMENTS / FEEDBACK FROM IRB MEMBERS, IF ANY

NA

F: SIGNATURE OF INSTITUTIONAL REVIEW BOARD CHAIRPERSON

Dr Aw Swee Eng	[signature]
Name	Signature

SUMMARY FOR 3rd PROTOCOL AMENDMENT

Approved Version: Version 5 dated 23 June 2008
Third Amended Version: Version 6 dated 8 Sep 2008

S/N	SECTION NAME/ NO.	PAGE NO. INVOLVED	AMENDED FROM	TO
			**VERSION 5 dated 23 June 2008**	**VERSION 6 dated 8 Sep 2008**
1	Content Page	2	The sequence of Assoc. Prof. Pierce Chow's name in the original text:<br>Assoc. Prof. Pierce KH Chow	The sequence of Assoc. Prof. Pierce Chow's name has to be revised from:<br>'Assoc. Prof. Pierce KH Chow' to '**Assoc. Prof. Pierce Chow Kah Hoe**'
			The postal code of Principal Investigator, Assoc. Prof. Pierce KH Chow in the original text is '168608'	The postal code of Prinicpal Investigator 'Assoc. Prof. Pierce Chow' has to be revised to '169608'
		3	The sequence of Dr. Richard Lo's name in the original text:<br>Dr. Richard Hoau Gong Lo	The sequence of Dr. Richard Lo's name has to be revised from:<br>'Dr. Richard Hoau Gong Lo' to '**Dr. Richard Lo Hoau Gong**'
		3a	The original text do not have Page 3a	Page 3a is added with addition of the following Co-Investigator(s):<br><br>Dr. Sidney Yu Wing Kwong<br>Department of Nuclear Medicine<br>Singapore General Hospital<br>Outram Road<br>Singapore 169608<br>Tel: +65 6326 5666 Fax: +65 6224 0938<br>Email: gnmywk@sgh.com.sg<br><br>Mr. Somanesan<br>Department of Nuclear Medicine<br>Singapore General Hospital<br>Outram Road<br>Singapore 169608<br>Tel: +65 6326 5667 Fax: +65 6224 0938<br>Email: gnmss@sgh.com.sg

1	Content Page	3a	The original text do not have Page 3a	Assoc. Prof. Koo Wen Hsin<br>Department of Medical Oncology<br>National Cancer Centre<br>11 Hospital Drive<br>Singapore 169610<br>Tel: +65 6436 8000 Fax: +65 6227 2759<br>Email: dmokwh@nccs.com.sg<br><br>Assoc. Prof. Simon Ong<br>Department of Medical Oncology<br>National Cancer Centre<br>11 Hospital Drive<br>Singapore 169610<br>Tel: +65 6436 8000 Fax: +65 6227 2759<br>Email: dmooyk@nccs.com.sg
			The original text do not have Page 3a	Dr. Toh Han Chong<br>Department of Medical Oncology<br>National Cancer Centre<br>11 Hospital Drive<br>Singapore 169610<br>Tel: +65 6436 8000 Fax: +65 6227 2759<br>Email: dmothc@nccs.com.sg<br><br>Dr. Choo Su-Pin<br>Department of Medical Oncology<br>National Cancer Centre<br>11 Hospital Drive<br>Singapore 169610<br>Tel: +65 6436 8000 Fax: +65 6227 2759<br>Email: choosupin@nccs.com.sg

1	Content Page	3a	The original text do not have Page 3a	Dr. John Chia Whay Kuang<br>Department of Medical Oncology<br>National Cancer Centre<br>11 Hospital Drive<br>Singapore 169610<br>Tel: +65 6436 8000 Fax: +65 6227 2759<br>Email: nmocwk@nccs.com.sg<br><br>Dr. Ang Mei Kim<br>Department of Medical Oncology<br>National Cancer Centre<br>11 Hospital Drive<br>Singapore 169610<br>Tel: +65 6436 8000 Fax: +65 6227 2759<br>Email: Ang.Mei.Kim@nccs.com.sg
			The original text do not have Page 3a	Dr. Alexander Chung Yaw Fui<br>Department of Surgery<br>Singapore General Hospital<br>Outram Road<br>Singapore 169608<br>Tel: +65 6321 4051 Fax: +65 6220 9323<br>Email: alexander.chung.y.f@sgh.com.sg<br><br>Dr. Cheow Peng Chung<br>Department of Surgery<br>Singapore General Hospital<br>Outram Road<br>Singapore 169608<br>Tel: +65 6321 4051 Fax: +65 6220 9323<br>Email: gsucpc@sgh.com.sg

1	Content Page	3a	The original text do not have Page 3a	Prof. London Lucien Ooi Peng Jin<br>Department of Surgical Oncology<br>National Cancer Centre<br>11 Hospital Drive<br>Singapore 169610<br>Tel: +65 6436 8154 Fax: +65 6225 7559<br>Email: dsoopj@nccs.com.sg<br><br>Dr. Tan Yu Meng<br>Department of Surgical Oncology<br>National Cancer Centre<br>11 Hospital Drive<br>Singapore 169610<br>Tel: +65 6436 8000 Fax: +65 6225 7559<br>Email: Tan.Y.M@nccs.com.sg
			The original text do not have Page 3a	Assoc. Prof. Chow Wan Cheng<br>Department of Gastroenterology<br>Singapore General Hospital<br>Outram Road<br>Singapore 169608<br>Tel: +65 6321 4684 Fax: +65 62273623<br>Email: gm2cwc@sgh.com.sg<br><br>Dr. Tan Chee Kiat<br>Department of Gastroenterology<br>Singapore General Hospital<br>Outram Road<br>Singapore 169608<br>Tel: +65 6321 4684 Fax: +65 62273623<br>Email: gm2tck@sgh.com.sg
2	1. General Information<br>1.1 Contact Details	7	The sequence of Assoc. Prof. Pierce Chow's name in the original text:<br>Assoc. Prof. Pierce KH Chow	The sequence of Assoc. Prof. Pierce Chow's name has to be revised from:<br>'Assoc. Prof. Pierce KH Chow' to '**Assoc. Prof. Pierce Chow Kah Hoe**'

2	1. General Information<br>1.1 Contact Details	7	The Study Statistician is 'to be determined'	Dr. Chen Yu Ming<br>Singapore Clinical Research Institute<br>226 Outram Road<br>Blk A #03-02<br>Singapore 169039<br>Tel: +65 6325 7063 Fax: +65 6324 2700<br>Email: yuming@cteru.com.sg<br>is added as the Study Statistician
		8	The Study Monitor is 'to be determined'	Ms. Chua Yee Chien<br>Singapore Clinical Research Institute<br>226 Outram Road<br>Blk A #03-02<br>Singapore 169039<br>Tel: +65 6325 7097 Fax: +65 6324 2700<br>Email: yeechien@cteru.com.sg<br>is added as the Study Monitor
			The contact details of the Data Manager in the original text:<br>Ms. Li Choi Nar Priscilla<br>Senior Clinical Project Coordinator<br>Clinical Trials and Epidemiology Research Unit<br>226 Outram Road<br>Blk A #02-02<br>Singapore 169039	The contact details of the Data Manager in the original text has to be revised as follows:<br>Ms. Priscilla Li Choi Nar<br>Singapore Clinical Research Institute<br>226 Outram Road<br>Blk A #03-02<br>Singapore 169039
3	16. Deviations and Amendments<br>16.3 Early Stopping Due to Toxicity	58	The original text:<br>These toxicities will be reviewed by the IDSMC both at the formal interim analysis after the first 6 patients have been followed for 3 months, and continuously as grade 3 or 4 adverse events are reported.	'These toxicities will be reviewed by the IDSMC both at the formal interim analysis after the first 6 patients have been followed for 3 months, and continuously as grade 3 or 4 adverse events are reported.' has to be revised as 'These toxicities will be reviewed by the Safety Committee after the first 6 patients have been followed for 3 months, and continuously as grade 3 or 4 adverse events are reported.' with deletion of 'IDSM both at the formal interim analysis'

4	Appendix 4	74	The original text:<br>4 frames; 300"/ frame. 64 x 64 matrix Word mode. Image anterior and posterior abdomen. Image anterior and posterior thorax	'4 frames; 300"/ frame. 64 x 64 matrix Word mode. Image anterior and posterior abdomen. Image anterior and posterior thorax' has to be replaced by 'Anterior and posterior static images are acquired with 256 x 256 x 16 matrix size, full field of view for 180 seconds covering patient's lungs and liver'
5	Appendix 5	75	The original text:<br>4. Immediately following the break-through scan, perform 99m Tc MAA SPECT imaging of the liver (128 x 128 matrix, 120 views over 360, 20 sec/view, with attenuation correction)	'4. Immediately following the break-through scan, perform 99m Tc MAA SPECT imaging of the liver (128 x 128 matrix, 120 views over 360, 20 sec/view, with attenuation correction)' has to be revised as '4. Immediately following the break-through scan, perform 99m Tc MAA SPECT imaging of the liver (128 x 128 matrix, 120 views over 360, 20 sec/view)' with deletion of 'with attenuation correction'
			The original text:<br>The counts per pixel in a SPECT image corrected for attenuation (ignoring the partial volume effect) is directly proportional to activity per unit mass, and thus can be used as a surrogate.	'The counts per pixel in a SPECT image corrected for attenuation (ignoring the partial volume effect) is directly proportional to activity per unit mass, and thus can be used as a surrogate.' has to be revised as 'The counts per pixel in a SPECT image is directly proportional to activity per unit mass, and thus can be used as a surrogate.' with deletion of 'corrected for attenuation (ignoring the partial volume effect)'
6	Appendix 13	98	The orginal appendix include:<br>IDSMB<br>Independent Data and Safety Monitoring Board	'IDSMB - Independent Data and Safety Monitoring Board' has to be removed from this version.

Singapore General Hospital
SingHealth

Tel: (65) 6222 3322
Fax: (65) 6224 9221
Singapore General Hospital
Outram Road
Singapore 169608
www.sgh.com.sg

SINGAPORE GENERAL HOSPITAL
INSTITUTIONAL REVIEW BOARD
Outram Road
Singapore 169608
Tel: 6321 3518
Fax: 6222 1720

IRB APPROVAL FORM
(Amendments)

A: DETAILS OF STUDY

**IRB Ref. Number:**	305/2007
**Title Of Study:**	Phase I/ II Study of Sir-Spheres® Plus Sorafenib (Chemo-radiotherapy) as first line treatment in patients with non-resectable primary hepatocellular carcinoma. Protocol AHCC05
**Principal Investigator:**	A/Prof Pierce Chow, Senior Consultant, Dept of General Surgery
**Sponsor (if any):**	-

B: DOCUMENTS REVIEWED:

Document:

- Amended Protocol Ver 6 – 8 Sep 08

C: The IRB has reviewed the above-mentioned document, and has approved the proposed amendments. The IRB has also granted approval for the increase of target total number of SGH patients 30 and increase of target total recruitment number to 35.

The IRB operates in accordance with the ICH/ Singapore Guideline for Good Clinical Practices, and with the applicable regulatory requirement(s).

IRB Approval Date: 2 October 2008

D: THE FOLLOWING ARE TO BE OBSERVED UPON IRB APPROVAL

1. No subject should be admitted to the trial before the Health Sciences Authority issues the Clinical Trial Certificate (applicable only for drug trials). A copy of the Clinical Trial Certificate should be submitted to the IRB.

2 The Principal Investigator should ensure that this study is conducted in compliance with the Singapore Guideline for Good Clinical Practice, the ethical guidelines of which are applicable to all studies to be carried out, and to ensure that the study is carried out in accordance to the guidelines and the submitted protocol. The Principal Investigator should meet with his collaborator(s) regularly to assess the progress of the study, and be familiar and comply with all applicable research policies in the Institution.

3. This approval is valid for till **4 February 2009**.

4. No deviation from, or changes of, the protocol should be initiated without prior written IRB approval of an appropriate amendment, except when necessary to eliminate immediate hazards to the subjects or when the change(s) involve(s) only logistical or administrative aspects of the trial (e.g. change of monitor(s), telephone number(s).

5. Only the approved Patient Information Sheet and Consent Form should be used. It must be signed by each subject prior to enrolling in the study and initiation of any protocol procedures. Three copies of the Informed Consent Form should be signed and dated. Each subject or the subject's legally accepted representative should be given a copy of the signed consent form. The remaining 2 copies should be kept by the PI and in the case report respectively.

6. The Principal Investigator should promptly inform the IRB of:
   a. deviations from or changes of the protocol made to eliminate immediate hazards to the trial subjects.
   b. changes increasing the risk to subjects and/or affecting significantly the conduct of the trial.
   c. all adverse events within the stipulated time lines.
   d. new information that may affect adversely the safety of the subjects or the conduct of the trial.
   e. completion of the study.

7. Study Status Report should be submitted to the IRB for the following:
   a. Annual review: Status of the study should be reported to the IRB at least annually using the Study Status Report.
   b. Study renewal: the Study Status Report is to be submitted one month prior to expiry of the approval period.
   c. Study completion or termination: the Study Status Reports is to be submitted within three months of study completion or termination.

E: COMMENTS / FEEDBACK FROM IRB MEMBERS, IF ANY

Nil

F: SIGNATURE OF INSTITUTIONAL REVIEW BOARD CHAIRPERSON

Dr Aw Swee Eng	[signature]
Name	Signature

SUMMARY FOR 2nd PROTOCOL AMENDMENT

Approved Version: Version 3 dated 10 Apr 2008
Second Amended Version: Version 5 dated 23 Jun 2008

<table>
<tr><th rowspan="2">S/N</th><th rowspan="2">SECTION NAME/ NO.</th><th rowspan="2">PAGE NO. INVOLVED</th><th>AMENDED FROM</th><th>TO</th></tr>
<tr><th>VERSION 3 dated 10 Apr 2008</th><th>VERSION 5 dated 23 Jun 2008</th></tr>
<tr><td rowspan="3">1</td><td rowspan="3">Content Page</td><td rowspan="3">3</td><td>Dr Lai Hee Kit is not a Co-Investigator in this version</td><td>Dr Lai Hee Kiat is added as a Co-Investigator</td></tr>
<tr><td>The title of Tay Kiang Hiong is recorded as 'Dr'</td><td>The title of Dr Tay Kiang Hiong has been revised from 'Dr' to 'A/Prof'</td></tr>
<tr><td>The original text:
This study is supported by:
Bayer Schering Pharma
Sirtex Medical</td><td>It has to be revised from:
This study is supported by:
Bayer Schering Pharma
Sirtex Medical

to

This investigator-initiated study is supported by:
1. National Medical Research Council, Singapore
2. Bayer Schering Pharma
3. Sirtex Medical</td></tr>
</table>

2	Participation of the following invited centres is pending the approval of their IRB	3	This section is not included in this version	1. China (Beijing) – Cancer Institute Hospital; 2. India – TATA Memorial Centre; 3. Indonesia (Bali, Jakarta) – University of Udayana, University of Indonesia; 4. Korea (Seoul, Suwon) – Seoul National Bundang Hospital, St Vincent Hospital; 5. Malaysia (Selangor) – Hospital Selayang, University Malaya Hospital; 6. Myanmar (Yangon); 7. New Zealand (Auckland) – University of Auckland; 8. Philippines (Manila, Davao City) – Santo Toma, Davao Doctors Hospital; 9. Singapore – SGH, NCC, NUH, CGH, TTSH, AH; 10. Taiwan – Chang Gung Memorial Hospital; 11. Thailand (Bangkok) – National Cancer Institute; 12. Vietnam (Ha Noi, Ho Chi Minh City) – Cho Ray Hospital, K Hospital, Phu Tho Hospital, Viet Duc University Hospital
3	1. General Information<br>1.1 Contact Details	7	There is no Study Diagnostic Radiologist in this version	Assoc. Prof Thng Choon Hua<br>Department of Oncologic Radiology<br>National Cancer Centre<br>11 Hospital Drive<br>Singapore 169610<br>Tel: +65 6436 8010<br>Fax: +65 6226 5660<br>Email: dditch@nccs.com.sg<br>is added as the Study Diagnostic Radiologist

		8	The Study co-ordinator is to be determined	Ms. Ng Lin Eng<br>Clinical Trials Resource Centre<br>Singapore General Hospital<br>Outram Road<br>Singapore 169608<br>Tel: +65 6372 4753 Fax: +65 6220 9067<br>Email: ng.lin.eng@sgh.com.sg<br>is added as the Study Co-ordinator
4	9. Design of the Clinical Study	32	The existing flowchart has to be amended.	The content in the first text box has to be amended from 'Recruit: Eligible patients with unresectable primary HCC' to '**ELIGIBLE PATIENTS**'<br><br>Additional text '**SIRT**' has to be added between the first and the middle text box.<br><br>The content in the middle text box has to be revised as '**PHASE I: Patients commencing Sorafenib after SIRT 14 days or 11 days after SIRT**'<br><br>The content in the last text box has to be revised as '**PHASE II: Sorafenib commencing 11 or 14 days after SIRT**'
			The original text:<br>SIR-Spheres will be administered at the calculated (patient-specific) activity, described in Section 1**2**: Treatment	There is a typo error of the section number for the sentence 'SIR-Spheres will be administered at the calculated (patient-specific) activity, described in Section 12: Treatment '. The section number should be amended from 1**2** to 1**1**.

5	9.1 Patient Eligibility	33	The original text:<br>a) Histology consistent with HCC and its histological variants such as poorly differentiated HCC and sacomatoid HCC or	'a) Histology consistent with HCC and its histological variants such as poorly differentiated HCC and sacomatoid HCC or' has to be revised as ' 'a) Histology consistent with HCC and its histological variants such as poorly differentiated HCC and sacomatoid HCC **done at anytime** or' with addition of 'done at anytime'
			The original text:<br>b) Space occupying lesion of the liver demonstrated by ultrasound, CT scan (non-dynamic) or MRI (non-dynamic) and either :<br>Serum alpha-feto protein level of at least 400 mcg/L or	'b) Space occupying lesion of the liver demonstrated by ultrasound, CT scan (non-dynamic) or MRI (non-dynamic) and either :<br>Serum alpha-feto protein level of at least 400 mcg/L or ' has to be revised as 'b) Space occupying lesion of the liver demonstrated by ultrasound, CT scan (non-dynamic) or MRI (non-dynamic) and ~~either~~ :<br>Serum alpha-feto protein level of at least 400 mcg/L **done at anytime** or' with deletion of the word 'either' and addition of 'done at anytime'
			The original text:<br>c) Radiological evidence of HCC by dynamic contrast-enhanced CT scan or dynamic contrast-enhanced MRI* and serology positive for Hepatitis B or C and alpha-feto protein above normal range	c) Radiological evidence of HCC by dynamic contrast-enhanced CT scan or dynamic contrast-enhanced MRI* and serology positive for Hepatitis B or C and alpha-feto protein above normal range' has to be revised as 'c) Radiological evidence of HCC by dynamic contrast-enhanced CT scan or dynamic contrast-enhanced MRI* and serology positive for Hepatitis B or C and alpha-feto protein above normal range **done at anytime**' with addition of 'done at anytime'

			The original text:<br>In order to be considered eligible for the study and to receive study treatment, patients must fulfil the inclusion and exclusion criteria specified in **10**.2 and **10**.3 below.	There is a typo error of the numbers for the sentence 'In order to be considered eligible for the study and to receive study treatment, patients must fulfil the inclusion and exclusion criteria specified in 10.2 and 10.3 below'. The section number should be amended from **10** to **9**.
6	9.2 Inclusion Criteria	34	The original text:<br>Total bilirubin < 2.0 mg/dL (SIR-Spheres should not be administered as a whole liver treatment if the total bilirubin is ≥ 2X the institutional upper limit of normal).	There are 2 typo errors of the symbol for Total bilirubin. It has to be revised from 'Total bilirubin < 2.0 mg/dL (SIR-Spheres should not be administered as a whole liver treatment if the total bilirubin is ≥ 2X the institutional upper limit of normal).' to 'Total bilirubin **≤** 2.0 mg/dL (SIR-Spheres should not be administered as a whole liver treatment if the total bilirubin is **>** 2X the institutional upper limit of normal).'
			The original text:<br>(i) Life expectancy of at least 3 months without any active treatment. This is defined as a patient who has OKUDA I and II inoperable HCC.	(i) Life expectancy of at least 3 months without any active treatment. This is defined as a patient who has OKUDA I **and** II inoperable HCC.' has to be revised to '(i) Life expectancy of at least 3 months without any active treatment. This is defined as a patient who has OKUDA I **or** II inoperable HCC.' with the word '**and**' replaced by '**or**'.
7	9.3.1 Contraindications to SIR-Spheres	35	The original text:<br>c) Abnormal synthetic and excretory liver function tests (LFTs) as determined by serum albumin (must be ≤ 2.5 g/dL) and total bilirubin (must be > 2.0 mg/dL), respectively	There is a typo error of the symbol for serum albumin. It should be < 2.5 g/dL instead of ≤ 2.5 g/dL. Therefore, it should be revised as<br>c) Abnormal synthetic and excretory liver function tests (LFTs) as determined by serum albumin (must be **<** 2.5 g/dL) and total bilirubin (must be > 2.0 mg/dL), respectively
8	10.3 Haematological and Serological Assessment	37	'Complete blood count' is the only investigation that need to be completed within 28 days of study entry under 'Haematological'	'**INR, Prothrombin Time and Prothrombin Time (control)**' are added as another investigation that need to be completed within 28 days of study entry under 'Haematological'

9	11.1.3 Administration of SIR-Spheres	45	'Bremstrahlung scan will be performed in the Department of Nuclear Medicine & PET at 4 - 24 hours after the SIR-Spheres therapy procedure, to visualize the in vivo distribution of the administered SIR-Spheres' is not included in this version.	'Bremstrahlung scan will be performed in the Department of Nuclear Medicine & PET at 4 - 24 hours after the SIR-Spheres therapy procedure, to visualize the in vivo distribution of the administered SIR-Spheres' is added under section 11.1.3 Administration of SIR-Spheres
10	12.1 Eligibility Assessment	50	The original text:<br>'Complete blood count' is listed under '12.1 Eligiblity Assessment'	'**INR, Prothrombin Time and Prothrombin Time (control)**' are added behind 'Complete blood count'
11	12.2 Serial Study Measurements	50	The original text in the table of 12.2 Serial Study Measurements is:<br>'CBC and platelets'	'**and Prothrombin Time and Prothrombin Time (control)**' are added behind 'CBC and platelets'
			The original text in the table of 12.2 Serial Study Measurements for 'Quality of life assessment' with the detail is recorded as '12 weekly until progression'	The detail of 'Quality of life assessment' has to be revised from '**12 weekly until progression**' to '**Baseline and every visit thereafter plus at first progression of disease**'
12	Table 12.2 Study Treatment Plan	51	There is no 'Prothrombin Time & Prothrombin Time (Control)' in the table 12.2 Study Treatment Plan	'Prothrombin Time and Prothrombin Time (control)' are added.
			There is no 'INR' in the table 12.2 Study Treatment Plan	'INR' is added.
13	Notes to Study Plan	52	The original text:<br>(g) EQ-5D Quality of life questionnaires filled out at baseline, 3, 6, 12, 24, 36 months then yearly thereafter plus at first progression of disease.	The sentence has to be revised from '(g) EQ-5D Quality of life questionnaires filled out at baseline, 3, 6, 12, 24, 36 months then yearly thereafter plus at first progression of disease.' to '(g) EQ-5D Quality of life questionnaires filled out at baseline and every visit thereafter plus at first progression of disease.' The text ', 3, 6, 12, 24, 36 months then yearly' has been deleted and replaced by 'and every visit'.

14	14.1 Toxicity and Safety (Primary Endpoint)	54	The original text:<br>Defintions and requirements for reporting adverse events (AEs) and serious adverse events (SAEs) are detailed in section **<u>18</u>**.	There is a typo error of the number for the sentence 'Defintions and requirements for reporting adverse events (AEs) and serious adverse events (SAEs) are detailed in section 17'. The section number should be amended from **<u>18</u>** to 17.
15	14.5 Quality of Life (Secondary Endpoint)	55	The original text:<br>The EQ-5D (see Appendix 9) will be collected at baseline, prior to commencing protocol treatment and then at 3, 6, 12, 24 and 36 month intervals, then yearly thereafter.	The sentence has to be revised from 'The EQ-5D (see Appendix 9) will be collected at baseline, prior to commencing protocol treatment and then at 3, 6, 12, 24 and 36 month intervals, then yearly thereafter.' to 'The EQ-5D (see Appendix 9) will be collected at baseline, prior to commencing protocol treatment and every visit thereafter' . The text ' then at 3, 6, 12, 24 and 36 month intervals, then yearly' has been deleted and replaced by 'every visit'.
16	Appendix 4	74	The original dose is '150MBq'	The dose has to be revised from '150MBq' to '150MBq to 200MBq'
			Interpretation: If lung/ liver ratio is > 10% then there is need for dose reduction of SIR-Spheres' is included as part of Appendix 4	'Interpretation: If lung/ liver ratio is > 10% then there is need for dose reduction of SIR-Spheres' has to be removed from Appendix 4

Singapore General Hospital
SingHealth

Tel: (65) 6222 3322
Fax: (65) 6224 9221
Singapore General Hospital
Outram Road
Singapore 169608
www.sgh.com.sg

SINGAPORE GENERAL HOSPITAL
INSTITUTIONAL REVIEW BOARD
Outram Road
Singapore 169608
Tel: 6321 3518
Fax: 6222 1720

IRB APPROVAL FORM
(Amendments)

A: DETAILS OF STUDY

**IRB Ref. Number:**	305/2007
**Title Of Study:**	Phase I/ II Study of Sir-Spheres® Plus Sorafenib (Chemo-radiotherapy) as first line treatment in patients with non-resectable primary hepatocellular carcinoma. Protocol AHCC05
**Principal Investigator:**	A/Prof Pierce Chow, Senior Consultant, Dept of General Surgery
**Sponsor (if any):**	-

B: DOCUMENTS REVIEWED:

Document:

- Protocol Amendment Ver 5.0 dated 23 Jun 08
- Amended Patient Information Sheet & Consent Form Ver 4 dated 5 June 08

C: The IRB has reviewed the above-mentioned documents, and has approved the proposed amendments.

The IRB operates in accordance with the ICH/ Singapore Guideline for Good Clinical Practices, and with the applicable regulatory requirement(s).

**IRB Approval Date:**	27 June 2008

D: THE FOLLOWING ARE TO BE OBSERVED UPON IRB APPROVAL

1. No subject should be admitted to the trial before the Health Sciences Authority issues the Clinical Trial Certificate (applicable only for drug trials). A copy of the Clinical Trial Certificate should be submitted to the IRB.

2 The Principal Investigator should ensure that this study is conducted in compliance with the Singapore Guideline for Good Clinical Practice, the ethical guidelines of which are applicable to all studies to be carried out, and to ensure that the study is carried out in accordance to the guidelines and the submitted protocol. The Principal Investigator should meet with his collaborator(s) regularly to assess the progress of the study, and be familiar and comply with all applicable research policies in the Institution.

3. This approval is valid for till **4 February 2009**.

4. No deviation from, or changes of, the protocol should be initiated without prior written IRB approval of an appropriate amendment, except when necessary to eliminate immediate hazards to the subjects or when the change(s) involve(s) only logistical or administrative aspects of the trial (e.g. change of monitor(s), telephone number(s).

5. Only the approved Patient Information Sheet and Consent Form should be used. It must be signed by each subject prior to enrolling in the study and initiation of any protocol procedures. Three copies of the Informed Consent Form should be signed and dated. Each subject or the subject's legally accepted representative should be given a copy of the signed consent form. The remaining 2 copies should be kept by the PI and in the case report respectively.

6. The Principal Investigator should promptly inform the IRB of:
   a. deviations from or changes of the protocol made to eliminate immediate hazards to the trial subjects.
   b. changes increasing the risk to subjects and/or affecting significantly the conduct of the trial.
   c. all adverse events within the stipulated time lines.
   d. new information that may affect adversely the safety of the subjects or the conduct of the trial.
   e. completion of the study.

7. Study Status Report should be submitted to the IRB for the following:
   a. Annual review: Status of the study should be reported to the IRB at least annually using the Study Status Report.
   b. Study renewal: the Study Status Report is to be submitted one month prior to expiry of the approval period.
   c. Study completion or termination: the Study Status Reports is to be submitted within three months of study completion or termination.

E: COMMENTS / FEEDBACK FROM IRB MEMBERS, IF ANY

Nil

F: SIGNATURE OF INSTITUTIONAL REVIEW BOARD CHAIRPERSON

Dr Aw Swee Eng
Name

[signature]
Signature

G: COMPOSITION OF INSTITUTIONAL REVIEW BOARD

Name	IRB Membership	Designation	Gender
Dr Aw Swee Eng	Chairperson	Senior Consultant, Dept of Nuclear Medicine & PET	Male
Dr Yu Su Ling	Deputy Chairperson	Senior Consultant, Dept of Obstetrics & Gynaecology	Female
Prof Ho Lai Yun	Member	Senior Consultant, Dept of Neonatal & Developmental Medicine	Male
Dr Hsu Li Fern	Member	Consultant, Dept of Cardiology, NHC	Male
Dr Peter Lim Khek Keong	Member	-	Male

Singapore
General Hospital
SingHealth

Tel: (65) 6222 3322
Fax: (65) 6224 9221
Singapore General Hospital
Outram Road
Singapore 169608
www.sgh.com.sg

SINGAPORE GENERAL HOSPITAL
INSTITUTIONAL REVIEW BOARD
Outram Road
Singapore 169608
Tel: 6321 3518
Fax: 6222 1720

IRB APPROVAL FORM (Amendments)

A: DETAILS OF STUDY

**IRB Ref. Number:**	305/2007
**Title Of Study:**	Phase I/ II Study of Sir-Spheres® Plus Sorafenib (Chemo-radiotherapy) as first line treatment in patients with non-resectable primary hepatocellular carcinoma. Protocol AHCC05e
**Principal Investigator:**	A/Prof Pierce Chow, Senior Consultant, Dept of General Surgery
**Sponsor (if any):**	-

B: DOCUMENTS REVIEWED:

Document:

- Amended Protocol Ver 3 dated 10 Apr 08

C: The IRB has reviewed the above-mentioned document, and has approved the proposed amendments.

The IRB operates in accordance with the ICH/ Singapore Guideline for Good Clinical Practices, and with the applicable regulatory requirement(s).

**IRB Approval Date:**	9 May 2008

D: THE FOLLOWING ARE TO BE OBSERVED UPON IRB APPROVAL

1. No subject should be admitted to the trial before the Health Sciences Authority issues the Clinical Trial Certificate (applicable only for drug trials). A copy of the Clinical Trial Certificate should be submitted to the IRB.

2 The Principal Investigator should ensure that this study is conducted in compliance with the Singapore Guideline for Good Clinical Practice, the ethical guidelines of which are applicable to all studies to be carried out, and to ensure that the study is carried out in accordance to the guidelines and the submitted protocol. The Principal Investigator should meet with his collaborator(s) regularly to assess the progress of the study, and be familiar and comply with all applicable research policies in the Institution.

3. This approval is valid for till **4 February 2009**.

4. No deviation from, or changes of, the protocol should be initiated without prior written

IRB approval of an appropriate amendment, except when necessary to eliminate immediate hazards to the subjects or when the change(s) involve(s) only logistical or administrative aspects of the trial (e.g. change of monitor(s), telephone number(s).

5. Only the approved Patient Information Sheet and Consent Form should be used. It must be signed by each subject prior to enrolling in the study and initiation of any protocol procedures. Three copies of the Informed Consent Form should be signed and dated. Each subject or the subject's legally accepted representative should be given a copy of the signed consent form. The remaining 2 copies should be kept by the PI and in the case report respectively.

6. The Principal Investigator should promptly inform the IRB of:
   a. deviations from or changes of the protocol made to eliminate immediate hazards to the trial subjects.
   b. changes increasing the risk to subjects and/or affecting significantly the conduct of the trial.
   c. all adverse events within the stipulated time lines.
   d. new information that may affect adversely the safety of the subjects or the conduct of the trial.
   e. completion of the study.

7. Study Status Report should be submitted to the IRB for the following:
   a. Annual review: Status of the study should be reported to the IRB at least annually using the Study Status Report.
   b. Study renewal: the Study Status Report is to be submitted one month prior to expiry of the approval period.
   c. Study completion or termination: the Study Status Reports is to be submitted within three months of study completion or termination.

E: COMMENTS / FEEDBACK FROM IRB MEMBERS, IF ANY

Nil

F: SIGNATURE OF INSTITUTIONAL REVIEW BOARD CHAIRPERSON

Dr Aw Swee Eng
Name

[signature]
Signature

G: COMPOSITION OF INSTITUTIONAL REVIEW BOARD

**Name**	**IRB Membership**	**Designation**	**Gender**
Dr Aw Swee Eng	Chairperson	Senior Consultant, Dept of Nuclear Medicine & PET	Male
Dr Yu Su Ling	Deputy Chairperson	Senior Consultant, Dept of Obstetrics & Gynaecology	Female
Dr K Puvanendran	Member	Senior Consultant, Dept of Neurology	Male
Dr Ho Kah Leng	Member	Associate Consultant, Cardiology, NHC	Female
Mr Kim Seah Teck Kim	Member	Partner, A.Ang, Seah and Hoe	Male
Dr Peter Lim Khek Keong	Member	-	Male

Asia-Pacific
Hepatocellular Carcinoma Trials Group

Protocol AHCC05

PHASE I/II STUDY OF SIR-SPHERES® PLUS SORAFENIB (CHEMO-RADIOTHERAPY) AS FIRST LINE TREATMENT IN PATIENTS WITH NON-RESECTABLE PRIMARY HEPATOCELLULAR CARCINOMA

April 2008

PHASE I/II STUDY OF SIR-SPHERES® PLUS SORAFENIB (CHEMO-RADIOTHERAPY) AS FIRST LINE TREATMENT IN PATIENTS WITH NON-RESECTABLE PRIMARY HEPATOCELLULAR CARCINOMA

Principal Investigator:

Assoc. Prof. Pierce KH Chow
Senior Consultant Hepato-Biliary Surgeon
Singapore General Hospital, Outram Road
Singapore 168608
Tel: +65 63214051
Fax: +65 62209323
Email: gsupc@singnet.com.sg

Duke-NUS Graduate Medical School Singapore
2 Jalan Bukit Merah
Singapore 169547
Tel: +65 6516 7666
Fax: +65 6224 6242
Email: pierce.chow@gms.edu.sg

Co-Investigators:

Prof. Soo Khee Chee
11 Hospital Drive
National Cancer Centre
Singapore 169610
Tel: +65 6436 8205
Fax: +65 6220 7759
Email: admskc@nccs.com.sg

Assoc. Prof. Tan Say Beng
Clinical Trials and Epidemiology Research Unit
226 Outram Road
Blk A #03-02
Singapore 169039
Tel: +65 6325 7060
Fax: +65 6324 2700
Email: saybeng@cteru.com.sg

Dr. Anthony Goh Soon Whatt
Department of Nuclear Medicine
Singapore General Hospital
Outram Road
Singapore 169608

Tel: +65 6321 4649
Fax: + 65 6224 0938
Email: anthony.goh.s.w@sgh.com.sg

Dr. Tay Kiang Hiong
Department of Diagnostic Radiology
Singapore General Hospital
Outram Road
Singapore 169608
Tel: +65 6326 5029
Fax: + 65 6326 5161
Email: tay.kiang.hiong@sgh.com.sg

Dr. Richard Hoau Gong Lo
Department of Diagnostic Radiology
Singapore General Hospital
Outram Road
Singapore 169608
Tel: +65 6321 4409
Fax: + 65 6224 1407
Email: richard.lo.h.g@sgh.com.sg

Dr. Donald Poon Yew Hee
Department of Medical Oncology
National Cancer Centre Singapore
11 Hospital Drive
Singapore 169610
Tel: +65 6436 8000
Fax: +65 6324 0875
Email: dmopyh@nccs.com.sg

This study is supported by: Bayer Schering Pharma
Sirtex Medical

TABLE OF CONTENTS

1 GENERAL INFORMATION 7
1.1 Contact Details 7
2 OVERALL SYNOPSIS OF THE CLINICAL INVESTIGATION 9
3 APPROVAL AND AGREEMENT 10
4 THE PRODUCT 11
4.1 Product Description 11
4.1.1 Mode of Action 11
4.1.2 Form and Stability 11
4.2 Regulatory Status 12
4.2.1 Australia 12
4.2.2 USA 12
4.2.3 European Union 12
4.2.4 Singapore 12
4.3 Manufacture ...12
4.4 Radiation Safety 12
4.4.1 Radiation Exposure 13
5 PRELIMINARY INVESTIGATIONS, PREVIOUS CLINICAL EXPERIENCE AND JUSTIFICATION FOR THE STUDY 15
5.1 Epidemiology and Prognosis of Hepatocellular Carcinoma ...15
5.2 Current Treatment Options for Hepatocellular Carcinoma ...15
5.2.1 Surgical Resection 15
5.2.2 Liver Transplantation 15
5.2.3 Trans-catheter Arterial Chemoembolisation (TACE) 15
5.2.4 Sorafenib 16
5.2.5 Selective Internal Radiation Therapy (SIRT) 18
5.3 Clinical Experience with SIR-Spheres ...19
5.4 Rationale for SIRT Plus Sorafenib Therapy ...20
6 PRECLINICAL TESTING OF SIR-SPHERE 22
6.1 Biocompatibility Safety Data .. 22
6.2 Toxicology .. 22
6.2.1 Localised Toxicity 22
6.2.2 Systemic Toxicity 23
6.3 Mutagenicity .. 23
6.4 Cytotoxicity .. 24
6.5 Haemocompatibility .. 24
6.6 Sensitising Ability .. 24
6.7 Summary .. 25
7 CLINICAL RISK ANALYSIS 26
7.1 Clinical Rationale .. 26
7.1.1 Complications and Toxic Effects of SIR-Spheres 26
7.1.2 Identifying the Specific Risks of SIR-Spheres 28
7.1.3 Identifying the Risks of Combining SIR-Spheres With Sorafenib 29
8 OBJECTIVES 31
9 DESIGN OF THE CLINICAL STUDY 32
9.1 Patient Eligibility 32

9.2 Inclusion Criteria 33
9.3 Exclusion Criteria 35
9.3.1 Contraindications to SIR-Spheres 35
9.3.2 Exclusion Criteria Specific to This Investigational Study 35
10 STUDY ENTRY 37
10.1 Patient Screening 37
10.2 Clinical Assessment 37
10.3 Haematological and Serological Assessment 37
10.4 Radiological Assessment 38
10.4.1 CT Scan of the Liver and Abdomen/Pelvis 38
10.4.2 CT Scan of the Chest 38
10.5 Assessment of Patient Suitability for Selective Internal Radiation Therapy 38
10.5.1 Hepatic Angiogram 38
10.5.2 Tc-99 MAA Lung Shunt Study 39
10.6 Commencement of Treatment 40
11 TREATMENT 41
11.1 Protocol Treatment: SIR-Spheres Therapy 41
11.1.1 Calculation of SIR-Spheres Activity to Deliver Using the Partition Model 41
11.1.2 Calculation of SIR-Spheres Activity to Deliver Using the Body Surface Area 43
11.1.3 Administration of SIR-Spheres 44
11.1.3.1 Measurement of Residual SIR-Spheres Activity Post-Treatment 45
11.2 Protocol Treatment: Sorafenib 45
11.2.1 Precautions 45
11.2.2 Duration of Treatment 45
11.2.3 Sorafenib Dose and Schedule Modifications 46
11.2.3.1 Dose Modification of Sorafenib for Skin Toxicity 47
11.2.4 Cessation of Protocol Treatment 48
11.2.5 Non-Protocol Chemotherapy 49
11.2.6 Supportive (Non-Protocol) Treatment 49
11.3 Concomitant Medications 49
12 INVESTIGATIONS 50
12.1 Eligibility Assessment 50
12.2 Serial Study Measurements 50
13 FOLLOW-UP 53
14 CRITERIA FOR ASSESSING OUTCOME 54
14.1 Toxicity and Safety (Primary Endpoint) 54
14.2 Tumour Response Rate (Secondary Endpoint) 54
14.2.1 RECIST Guidelines 54
14.2.2 Response Criteria 54
14.3 Progression-Free Survival (Secondary Endpoint) 55
14.4 Survival (Secondary Endpoint) 55
14.5 Quality of Life (Secondary Endpoint) 55
15 STATISTICAL CONSIDERATIONS & METHODOLOGY 56
15.1 Study Design and Sample Size 56
16 DEVIATIONS AND AMENDMENTS 58
16.1 Deviations From the Study Protocol 58
16.2 Amendments to the Study Protocol 58

16.3 Early Stopping Due to Toxicity 58
17 ADVERSE EVENTS 59
17.1 Definitions 59
17.1.1 Adverse event (AE) (ISO 14155-1:2003).......... 59
17.1.2 Serious adverse device effect (ISO 14155-1:2003).......... 59
17.1.3 Serious adverse event (SAE) (ISO 14155-1:2003).......... 59
17.2 Reporting 59
17.3 Pregnancy During the Study 60
18 ETHICAL CONSIDERATIONS 61
18.1 Informed Consent 61
18.2 Confidentiality 61
18.3 Changes to Final Study Plan 61
19 PUBLICATION POLICY 62
20 ADMINISTRATIVE PROCEDURE 63
20.1 Investigator File 63
20.2 Quality Assurance 63
20.3 Study Completion 63
21 BIBLIOGRAPHY 64
APPENDIX 1 ECOG Performance Status (Definitions) 68
APPENDIX 2 Consumer Medicine Information For Sorafenib 69
APPENDIX 3 Technique for Administration of SIR-Spheres 71
APPENDIX 4 Nuclear Medicine Tc-99 MAA Lung Shunt Study 74
APPENDIX 5 SIR-Spheres Radiation Absorbed Dose/ Activity Calculation Procedure 75
APPENDIX 6 Example SIR-Spheres Radiation Absorbed Dose/Activity Calculation ... 77
APPENDIX 7 SIR-Spheres Administered Activity Calculation Tables 78
APPENDIX 8 NCI CTC Version 3 Recommendation for Grading of Acute and Subacute Toxicity 82
APPENDIX 9 EQ-5D Quality of Life Question 83
APPENDIX 10 World Medical Association Declaration of Helsinki 88
APPENDIX 11 Informed Consent Form 92
APPENDIX 12 Patient Information Sheet 93
Introduction 93
Purpose of Study 93
Who can be in the study? 94
Patient's Responsibility 94
Possible Side Effects 95
Anticipated expense 96
Confidentiality 96
Contact person 96
Voluntary participation 97
APPENDIX 13 Abbreviations 98

1 GENERAL INFORMATION

1.1 Contact Details

Principal Investigator:

Assoc. Prof. Pierce KH Chow
Duke-NUS Graduate Medical School Singapore
2 Jalan Bukit Merah
Singapore 169547
Tel: +65 6516 7666
Fax: +65 6224 6242
Email: pierce.chow@gms.edu.sg

Study Statistician:

(To be determined)
Biostatistics Unit
Division of Clinical Trials and Epidemiological Sciences
National Cancer Centre Singapore
11 Hospital Drive
Singapore 169610

Assoc. Prof. Tan Say Beng
Clinical Trials and Epidemiology Research Unit
226 Outram Road
Blk A #03-02
Singapore 169039
Tel: +65 6325 7060
Fax: +65 6324 2700
Email: saybeng@cteru.com.sg

**Study Coordinator:**	***(To be determined)***<br>Clinical Trials Office<br>Division of Clinical Trials and Epidemiological Sciences<br>National Cancer Centre Singapore<br>11 Hospital Drive<br>Singapore 169610
**Study Monitor:**	***(To be determined)***
**Data Manager**	Ms. Li Choi Nar Priscilla<br>Senior Clinical Project Coordinator<br>Clinical Trials and Epidemiology Research Unit<br>226 Outram Road<br>Blk A #02-02<br>Singapore 169039<br>Tel: +65 6325 7081<br>Fax: +65 6324 2700<br>Email: priscilla@cteru.com.sg

2 OVERALL SYNOPSIS OF THE CLINICAL INVESTIGATION

This Phase I/II trial will evaluate the safety and activity of chemo-radiotherapy comprising a regimen of Sorafenib chemotherapy plus SIR-Spheres yttrium-90 microspheres (chemo-radiotherapy, also known as "chemo-SIRT"), for first-line treatment of patients with primary hepatocellular carcinoma (HCC) in whom surgical resection is not feasible.

This study is designed as a prelude to a planned future randomised comparative study that will compare the efficacy of Sorafenib plus SIR-Spheres versus Sorafenib alone, in this patient population.

3 APPROVAL AND AGREEMENT

I have read and understand the requirements of this study protocol, "Phase I/II study of SIR-Spheres plus Sorafenib (chemo-radiotherapy) as first line treatment in patients with non-resectable primary hepatocellular carcinoma". I agree to treat all patients entered in this study as per the protocol and as per applicable regulations and guidelines, and maintain the appropriate records and documentation required. I will ensure that all staff participating in the study will be appropriately trained and informed of their responsibilities and obligations on the study.

________________________________   ____________________

Investigator Signature   Date (dd, mm, yyyy)

____

Investigator Name

____

Institution

4 THE PRODUCT

4.1 Product Description

SIR-Spheres consist of biocompatible microspheres containing yttrium-90 with a size between 20 and 30 microns in diameter. Yttrium-90 is a high-energy pure beta-emitting isotope with no primary gamma emission. The maximum energy of the beta particles is 2.27MeV with a mean of 0.93MeV. The maximum range of these emissions in tissue is 11mm with a mean of 2.5mm. The half-life of yttrium-90 is 64.1 hours. In use requiring the isotope to decay to infinity, 94% of the radiation is delivered in 11 days, leaving only background level radiation, which has no therapeutic value. SIR-Spheres themselves are a permanent implant. Each device is for single patient use.

4.1.1 Mode of Action

Intrinsic to the concept of Selective Internal Radiation Therapy (SIRT) is the preferential placement of the radioactive microspheres selectively into tumours rather than healthy liver tissue. This concept exploits the blood supply to hepatic tumours, which is derived predominantly from the hepatic artery. By delivering the microspheres into the hepatic artery, they are selectively delivered to tumours whereupon they lodge in the microvasculature of the tumour. This technique allows numerous and/or small tumours to be treated and can be used in patients who are not candidates for surgical resection. Placement of the microspheres into tumours via the hepatic artery is therefore fundamental to the use of this device.

Each device consists of sufficient microspheres to provide 3GBq (+/-10%) at the time and date of calibration (as shown on the label). The microspheres are suspended in sterile water for injection. Each vial of 3GBq (+/-10% at calibration) is dispatched in a volume of ≅5ml (microspheres and water together). This allows the required tumour activity to be manipulated as a volume.

4.1.2 Form and Stability

SIR-Spheres do not exhibit pharmacodynamics in the classic sense, but induce cell damage by emitting beta radiation. Once implanted, this device remains within the vasculature of hepatic tumours, with small amounts within the vasculature of normal liver parenchyma. The device is not phagocytised, nor does it dissolve or degrade after implantation. High dose radiation emitted from the device is cytocidal to cells within the range of the radiation. After the yttrium-90 has decayed, the non-radioactive microspheres remain intact and are not removed from the body.

The device has the potential to interact with other cytotoxic agents and is typically administered concomitantly with chemotherapeutic agents. This applies to chemotherapeutic agents applied for the purpose of managing either the same tumours targeted by the microspheres, or distant metastases. This interaction may be exploited to the benefit of the patient, in that there can be an additive toxicity on tumour cells, which can enhance the tumour cell kill rate. This interaction can also lead to additive toxicity on non-tumourous cells.

4.2 Regulatory Status

SIR-Spheres are regulated as a medical device product, based on international and U.S. Food and Drug Administration definitions of devices, and are classified as a sealed source brachytherapy device.

4.2.1 Australia

SIR-Spheres were listed on the Therapeutic Goods Administration (TGA) Australian Register of Therapeutic Goods in February 1998 as a medical device in accordance with the Therapeutic Goods Act 1989, under AUSTL No. 63369, with the following functional description:

"Yttrium 90 microspheres as an implantable source of beta radiation for the management of hepatic tumours".

4.2.2 USA

SIR-Spheres were approved by the **United States FDA** as a Class III medical device product via PMA P990065 in March 2002 for:

"the treatment of unresectable metastatic liver tumours from primary colorectal cancer together with adjuvant intra-hepatic artery chemotherapy (IHAC) of FUDR (Floxuridine)."

4.2.3 European Union

SIR-Spheres were approved in the European Union in October 2002 as an active implantable medical device under the Active Implantable Medical Device (AIMD) Directive (90/385/EEC), indicated for:

"the treatment of primary and secondary (metastatic) liver cancer."

4.2.4 Singapore

SIR-Spheres has been regulated as a Radioactive Material by the Centre of Radiation Protection Nuclear Science of the National Environment Agency since July 2005, used for:

"the treatment of patients with advance non-operable liver cancer."

4.3 Manufacture

The SIR-Spheres being supplied for this pilot study will be the same product approved for supply as above in Sections 4.1 and 4.2 which is manufactured under the approved Quality Management System processes by Sirtex Medical.

4.4 Radiation Safety

Personnel involved in any aspect of handling SIR-Spheres must be suitably qualified and be appropriately trained to deal specifically with this device. This includes nuclear medicine staff, staff involved in the implantation procedure and in post-implant care of the patient. Such staff require the support of a radiation safety officer or expert in radiation physics, and licenses for the facility will

normally require that such expertise is available to ensure safe use of isotopes within the facility.

Complete information may be found in the Radiation section in the Sirtex Training Manual supplied to all sites upon training. Additional copies may be obtained from Sirtex upon request.

4.4.1 Radiation Exposure

The following information on radiation exposure to the implantation team and to either nursing staff or visitors following the implantation is listed below in summary format. This data is presented in depth in the Sirtex Medical Training Manual, which is provided by Sirtex to all SIR-Spheres user sites as part of site training. Additional copies may be obtained from Sirtex upon request.

By way of comparison with the figures listed here, the radiation dose from normal background environmental radiation is 2 mSv per year.

Radiation dose levels expected for implantation staff:

The following exposure levels are representative for the technician or pharmacist preparing a typical patient dose, and for the physician implanting that prepared dose of SIR-Spheres.

		Trunk mSv	Lens of Eye mSv	Hands mSv
Technician	Shallow Dose (0.07 mm)<br>Deep dose (10 mm)	0.027<br>0.003	0.026<br>0.004	0.35
Physician	Shallow Dose (0.07 mm)<br>Deep dose (10 mm)	0.038<br>0.004	0.12<br>0.054	0.32
Radiation Safety Officer	Shallow Dose (0.07 mm)<br>Deep dose (10 mm)	< 0.02<br>0.01	0.04<br>0.017	0.2

International Commission on Radiological Protection (IRCP) Occupational Radiation Dose Limits are as follows:

Whole Body Effective Dose Limit:	20 mSv per year (averaged over 5 years) and no more than 50 mSv in any one year
Lens Equivalent Dose Limit:	150 mSv per year
Extremity (e.g. finger) Equivalent Dose Limit:	500 mSv per year over any 1 cm^2

These representative exposure levels are additive to other sources of exposure for workers.

Radiation doses to nursing staff or visitors:

The following dose rates may be expected at various distances from a patient with an implant of approximately 2 GBq when taken approximately 5 – 6 hours after implantation of SIR-Spheres.

Distance from patient, meters	Radiation dose, µSv/hr
0.25	18.8
0.5	9.2
1	1.5
2	0.4
4	<0.1

In the adjoining room at the wall immediately behind patient's bed-head the measurement was < 0.1 µSv/hr. Typical measurements within limits are 20 □µSv in any hour and 250 µSv in any seven days.

5 PRELIMINARY INVESTIGATIONS, PREVIOUS CLINICAL EXPERIENCE AND JUSTIFICATION FOR THE STUDY

5.1 Epidemiology and Prognosis of Hepatocellular Carcinoma

Primary hepatocellular carcinoma (HCC) is the fifth most common cancer in the world, and the third most common cause of cancer related death (Llovet, 2003). Primary HCC is estimated to occur at a global rate of more than 1 million new cases annually, with an increasing incidence rate (El Serag, 1999) and a predominance in developing countries. Only about 30% of patients are diagnosed early enough to benefit from potentially curative therapies, such as surgical resection, allogeneic liver transplantation or percutaneous ablation, which afford 5-year survival rates of 50 – 75 % (Bruix, 2002).

Most patients with primary HCC are diagnosed at intermediate to advanced stages of their disease, for which no generally accepted standard therapy exists (Llovet, 2003). In the absence of active therapy, median survival in unresectable HCC is 3 – 6 months, actuarial survival is 31% at one year, 8% at two years, and <3% at three years in the USA (SEER). Primary HCC generally leads to death as a consequence of local tumour growth, tissue destruction and liver dysfunction, rather than as a result of widespread extrahepatic disease.

5.2 Current Treatment Options for Hepatocellular Carcinoma

5.2.1 Surgical Resection

Surgical resection of the affected portion of the liver offers the best chance for disease-free survival. Unfortunately, most patients with HCC have tumour(s) that is(are) not amenable to surgical resection (multi-focal disease) or have other medical contraindications to surgery (limited hepatic reserve related to cirrhosis or chronic hepatitis) (Kassianides, 1987). Overall, fewer than 15% of patients with HCC are suitable surgical candidates.

5.2.2 Liver Transplantation

Orthotopic liver transplantation is also an option for some patients who meet the criteria; however, access to this alternative is limited by donor availability. Furthermore, patients with advanced HCC receiving liver transplants experience a significantly high recurrence rate (80% at 3 years for patients with advanced stage disease) (Marco-Alvarez, 1996; Mazzafero, 1996; Selby, 1995; Gores, 1993).

5.2.3 Trans-catheter Arterial Chemoembolisation (TACE)

Trans-catheter hepatic arterial chemoembolisation (TACE) combines the intra-arterial delivery of chemotherapy together with embolisation via a hepatic artery branch. Due to the importance of achieving local tumour control, TACE has become a widely used therapy when surgical resection is not an option. Chemotherapy inhibits HCC tumour cells, while embolisation deprives the tumour cells of blood-derived oxygen and nutrients.

Attempts to quantify and compare the therapeutic benefits of TACE have been hampered by the heterogeneity of institution-specific procedures, combining different chemotherapeutic drugs, embolising agents and administration schedules (Venook, 1994; Nerenstone, 1987; Ramming, 1983). Most early published studies were non-randomised comparisons and found survival in the order of 50% at one-year and 20% at two years (Carr, 2002). More recently, randomised have trials demonstrated

encouraging rates of tumour shrinkage (Llovet, 2002; Lo, 2002). TACE is usually well tolerated as long as cirrhosis is not severe. In patients with cirrhosis, TACE can cause severe liver damage; approximately 80% of patients with HCC have some form of cirrhosis.

Typically, TACE is delivered as a series of sequential treatments – similar to other forms of chemotherapy for solid tumours – with the number of treatments being determined by tumour burden and tumour distribution, and by the patient's liver tolerance to the procedure. TACE is performed as an outpatient procedure that typically requires 24 – 48 hours of hospitalisation for each treatment.

TACE is typically associated with significant local side effects including a characteristic post-embolisation syndrome comprising fatigue, abdominal pain, nausea and fever in 80% of patients. Serious complications occur in around 5% of cases, and 30-day post-treatment mortality is approximately 2%.

TACE is usually indicated for only approximately 10 – 15% of patients with unresectable HCC fulfilling the following criteria:

- A single large tumour (although complications are more frequent when largest diameter > 10 cm), or multiple tumours (but patients with very extensive disease are likely to have a poor response).
- Preserved liver function (serum bilirubin < 3.0 mg/dL, albumin > 2.8 g/dL).
- No portal vein (or branch) infiltration or thrombosis.

For those patients with unresectable HCC who do not fulfill these criteria, no generally accepted treatment option of proven efficacy exists at present.

5.2.4 Sorafenib

Conventional chemotherapy has no demonstrable efficacy in any randomised controlled HCC (Novak, Chow, Findlay 2005). Sorafenib (BAY 43-9006) is an oral multi-kinase inhibitor targeting several serine/threonine and receptor tyrosine kinases. An inhibitor of signal transduction, sorafenib prevents tumour cell proliferation and angiogenesis via its effects on the RAF/MEK/ERK pathway at the level of Raf kinase and tyrosine kinases VEGFR-2 and PDGFR-β.(11, 12) Subsequent biochemical and cellular mechanistic assays demonstrated activity against B-Raf and additional receptor tyrosine kinases, including vascular endothelial growth factor receptor-2, platelet derived growth factor receptor, Flt-3 and c-KIT.(13).

Sorafenib as a single agent has been evaluated globally in six Phase I trials, five Phase II trials and the ongoing Phase III trial in renal cell carcinoma (RCC) conducted by Bayer.

In addition, multiple Phase I studies of sorafenib in combination with other chemotherapeutic agents have been initiated. To date, over 1000 cancer patients have been exposed to single agent sorafenib. In addition, over 380 cancer patients have been exposed to sorafenib in combination with other chemotherapeutic agents in Phase I/Ib studies. Ongoing Phase II trials include a randomized

discontinuation trial in a number of different solid tumour patients, and uncontrolled studies in HCC, non-small cell lung cancer, metastatic breast cancer, head and neck cancer, and refractory CML.

Sorafenib has been generally well tolerated at a dose of 400 mg po twice daily (bid). The most common drug related adverse events have included hand-foot syndrome, diarrhea, fatigue, hypertension, pain and rash. Grade 3 and 4 drug-related adverse events were uncommon. There was no evidence of cumulative toxicity and the majority of the adverse events were reversible.

The five most common drug-related adverse events were hand-foot skin reaction, dermatology/skin-other, fatigue, anorexia and diarrhea. There was an increase in the number of serious adverse events, discontinuations due to adverse events, especially skin toxicities, as well as Grades 3 and 4, toxicities at the higher dose levels (greater than or equal to 600 mg bid). Hence, 400 mg bid was selected as the recommended dose for Phase II.

Preliminary antitumour activity has been reported in a variety of tumour types including renal cell cancer, hepatocellular cancer, melanoma, thyroid, acute myelogenous leukemia (AML), ovarian cancer, sarcoma, pancreatic cancer and colorectal cancer.

Six patients with HCC were included in Phase I trials, and remained on sorafenib for a median of 42 weeks (range 5-61 weeks). One partial response (PR) was observed and 4 patients had stable disease. These promising data led to the development of a phase II trial of sorafenib in HCC (Bayer study 10874).

Study 10874 was a multi-center, uncontrolled, phase II study evaluating the efficacy, safety, pharmacokinetics (PK), pharmacodynamics and tolerability of sorafenib in patients with advanced inoperable HCC. The study was conducted in the United States, Israel, Italy, France, and Belgium. Enrollment has been closed and fourteen patients are still receiving treatment as of 29 May 2004. Results are therefore preliminary and subject to change. Patients with measurable, histologically proven, inoperable HCC were eligible for this study. Patients with fibrolamellar variant or mixed histology were excluded. Sorafenib 400 mg was administered twice daily, and two dose reductions were permitted (to 200 mg bid and 200 mg once daily [qd]) for drug related toxicities. Treatment continued until evidence of tumour progression (radiological or clinical), or until appearance of unacceptable toxicities related to study drug. Tumour measurements (bidimensional) and assessment were done at baseline and every 8 weeks.

Study 10874 included 97 males and 40 females with a median age of 69 years. Eighty-four patients were over the age of 65. Sixty-eight patients had Eastern Cooperative Oncology Group (ECOG) Performance Status (PS) 0 and 69 had ECOG PS 1. Five patients received prior systemic treatment (one patient received neo-adjuvant adriamycin, two were treated with tamoxifen, and two with octreotide), and 56 patients received prior local therapy. Ninety-eight patients were Child-Pugh status A and 38 were Child-Pugh B. As of 7 April 2004, a total of more than 700 cycles were administered to 137 patients. A cycle was defined as 28 days. The median number of cycles delivered was 5 (range 1-17), and 31% of patients received 6 or more cycles. World Health Organization (WHO) criteria were used for evaluating objective tumour response. Five patients (4%) demonstrated a PR, defined as a reduction in tumour size of greater than 50%. There were 7 patients (5%) who did not meet the criteria for PR, but had a reduction in tumour size of greater than 25%, defined as a minor response (MR). All

responses were confirmed at 4-6 weeks. The median time to MR was 53 days (range 48-162) and the median time to PR was 109 days (range 49-296). A total of 75 patients (55%) had stable disease, documented at least six weeks after baseline. Twenty-four patients had progressive disease (PD). Median time to progression (TTP) was 168 days (range 1-577 days) and the estimated median overall survival (OS) was 284 days (range 0-475 days). The median duration of PR was 191 days (range 86-258 days), and the median duration of stable disease was 195 days (range 28-435 days). A multivariate analysis of prognostic factors was performed. Of the 14 disease characteristics included in the cox regression model, only alpha-fetoprotein (AFP) > 400, Child-Pugh status, and sodium > 140 mmol/l were statistically significant with p value < 0.05. Child-Pugh status was an independent predictor of progression free survival (PFS); PFS was 148 days (95% CI 114-195 days) in Child-Pugh A patients (n=96) and 78 days (95% CI 56-118) in Child-Pugh B patients (n=37); p=0.0172, hazard ratio 1.714. Child-Pugh A patients had a better outcome than those with Child-Pugh status B; in Child-Pugh A patients, median OS was 331 days and PFS at 6 and 12 months was 44% and 28% respectively. Patients with more favorable prognostic scores received more cycles of sorafenib. Sorafenib was generally well tolerated. The most frequent drug related toxicities were gastrointestinal (63%), dermatological (47%) and constitutional (34%). Treatment-emergent hepatic toxicities occurred in 20 (15%) patients of which 10 (7%) were grade 3 events and one (<1%) (increased bilirubin) was a grade 4 event. Doses were reduced, delayed and/or permanently discontinued due to toxicities that were considered related to the study drug in 81 (59%) patients. Thirty-four patients (25%) had the dose of study drug reduced due to adverse events, and nine patients had the dose level reduced twice. The most frequent reason for dose delay or discontinuation was hand-foot skin reaction in 16 (12%) patients. Other toxicities included diarrhea in 14 (10%) patients, fatigue in 11 (8%), increased bilirubin in 9 (6%) patients, increased alkaline phosphatase in 5 (4%) patients, and transaminase and amylase increases in 4 (3%) each. There were 67 deaths in the study. Most of the deaths were due to progressive cancer or hepatocellular disease and none of the deaths were related to study drug. In study 10874 grade 3 increase in aspartate transaminase (AST) occurred in 23 patients (17%) and grade 3 bilirubin elevation occurred in 24 patients (18%). No significant difference in safety was observed between patients with Child-Pugh A and Child-Pugh B cirrhosis. The frequency of serious adverse events was similar in Child-Pugh A and Child-Pugh B patients, and frequency of adverse events was equivalent among the two groups except for hyperbilirubinemia and elevated alkaline phosphatase, which were more common in Child-Pugh B patients, and dermatologic events, which were more common in Child-Pugh A patients. Although there was a greater proportion of deaths overall in the Child-Pugh B subpopulation, no deaths in the study were related to sorafenib, and most deaths in both groups were attributed to progression of HCC. The phase II program also includes study 100391, a randomized discontinuation (RD) study of sorafenib in advanced solid tumours. The study has enrolled 503 patients, and is currently closed to enrollment. Preliminary safety information from 100391 has revealed toxicities that are similar those seen in Phase I. Again, the 5 most frequent drug-related toxicities observed include hand-foot skin reaction, dermatology/skin-other, anorexia, diarrhea, and fatigue.

5.2.5 Selective Internal Radiation Therapy (SIRT)

Brachytherapy refers to the procedure of physically implanting permanent radiation sources into, next to, or through malignant tumours. Its history dates back to the discovery of radiation and since then has become established as an effective treatment modality in many organs. The most common applications for brachytherapy today involve prostate, uterine cervix, and head and neck malignancies. The key principle of brachytherapy involves the delivery of tumourcidal doses of radiation to the malignant tumour, but as a result of rapid radiation dose fall-off, minimal damage to adjacent normal tissues.

Currently, only a few specialized centers are able to place radiation sources manually into the liver, either percutaneously or via open laparotomy. A more convenient and broadly applicable technique utilizes yttrium-90 microspheres, which takes advantage of the unique vascular anatomy of the liver to preferentially implant hepatic tumours via selective hepatic arterial embolisation. This process is referred to as "Selective Internal Radiation Therapy" or SIRT.

It has long been established that the liver's *arterial* system supplies 80 - 100% of the blood to liver tumours (primary and metastatic); however, the normal liver derives nearly all of its blood flow from the parallel *portal venous* system. In addition, metastatic tumours in particular, form a dense arterial network with up to 200 times more vessels in plexi around tumours, compared to the normal liver tissues immediately nearby. This combination of vascular structure and local tumour feeding vessel concentration has led to the discovery that SIR-Spheres released into the hepatic artery will preferentially accumulate in the periphery of tumours in at least a 3:1, up to 20:1 ratio compared to normal liver (Kennedy, 2004; Campbell, 2000; Fox, 1991). Thus, the therapeutic index is favourable, in a manner similar to other brachytherapy approaches, e.g. prostate seed brachytherapy. The diameter of SIR-Spheres enables them to become implanted in the tumour microvasculature, but they are too large to pass through the end arterioles into the hepatic sinusoids, which have a restrictive diameter of 8-10 microns. Only if arterial-venous fistulae in the tumour are present with diameters of >30 microns, could SIR-Spheres pass into the next capillary bed, in the lung. The active moiety, yttrium-90 is a pure beta emitter which has an energy deposition and dose rate close to that of external beam radiotherapy, yet the effective range is under 3mm from the microsphere itself.

5.3 Clinical Experience with SIR-Spheres

Of all the implantations performed with SIR-Spheres globally (approximately 6,500 treatments at November 2007), approximately 50 – 60% of these patients have been treated for metastatic colorectal cancer.

In Australia, where SIR-Spheres were originally developed for the treatment of inoperable liver metastases from primary colorectal cancer, they were originally administered by injection through a surgically implanted hepatic artery port, immediately followed by hepatic artery chemotherapy (HAC) with either Floxuridine or 5-FU. Phase 1 and 2 clinical trials of SIRT in patients with colorectal liver metastases commenced at Royal Perth Hospital in Australia back 1987.

The original technique of delivering the SIR-Spheres intra-operatively was abandoned, as this procedure was poorly tolerated. Patients were subsequently treated by surgically implanting a hepatic artery port that was followed by administration of the SIR-Spheres approximately 10 days later. The use of surgically implanted ports has now also been abandoned in favor of administration of the SIR-Spheres via a temporary trans-femoral hepatic artery catheter, which is now the administration method of choice.

Over the past 5 years, SIR-Spheres have been increasingly been used as a treatment option in patients with inoperable HCC (Sangro, 2007). While SIR-Spheres are FDA approved with a Pre-Market Approval (PMA) specifically for the treatment of unresectable metastatic colorectal adenocarcinoma that has metastasized to the liver in combination with adjuvant intra-arterial FUDR, the device has a broader indication in the European Union, for the treatment of primary and secondary liver cancer. Consequently, SIR-Spheres have been used extensively in countries outside the US for the treatment of

patients with HCC, including Germany, Spain, Italy, Belgium, UK, Australia, Hong Kong and Singapore.

A typical dose of SIR-Spheres contains 30 – 50 million microspheres and delivers a radiation dose of approximately 100 – 150 Gray to the tumour tissue. Comparable tumour doses cannot normally be achieved safely via conventional external beam radiation therapy (EBRT).

In the management of HCC, SIR-Spheres are most commonly administered to the whole liver, resulting in high radiation doses being delivered to the predominantly arterial-fed tumours, while sparing the normal liver parenchyma, which receives most of its blood supply form the portal vein. Alternatively, SIR-Spheres may be administered selectively into the tumour-feeding arteries, which normally results in higher tumour radiation doses, compared to the whole liver approach.

SIR-Spheres are well tolerated by the non-cirrhotic liver and in those cirrhotic patients without ascites and in whom serum bilirubin is < 2.0 mg/dL. As such, SIRT has developed – predominantly in the European Union – as an alternative to TACE and TAE. These therapies are delivered as a series of sequential treatments and may produce significant side effects, including postembolisation syndrome, cholecystitis (Tarazov, 2000), acute renal failure (Huo, 2004), and a decline in liver function (Grieco, 2003). In comparison, SIRT requires a 24 hour hospital admission and most patients experience no side effects after being discharged. SIRT can be a valuable alternative for patients with tumours invading the portal vein and preserved liver function, a subset of patients for which no effective therapy can currently be offered (Sangro, 2007). SIRT is increasingly considered as an adjunct to liver transplantation since the prolonged period of disease control may result in patients not being dropped from the waiting list, and some patients may also benefit from down-staging following SIRT into liver transplant criteria (Kulik, 2005).

5.4 Rationale for SIRT Plus Sorafenib Therapy

Sorafenib is a prime candidate for investigation as part of a SIRT plus systemic chemotherapy combination for the treatment of patients with primary HCC.

Over the past decade, many chemotherapeutic and biologic agents have been developed for clinical use in oncology. Surgeons, radiation oncologists and medical oncologists have been investigating with much effort and enthusiasm the translation of these agents from the preclinical setting into treatment strategies for patients. Recently, for the first time ever a systemically administered agent has demonstrated statistically significant survival benefit in the management of patients with advanced HCC. The multi-targeted kinase inhibitor Sorafenib (Nexavar) demonstrated a survival benefit of 2.9 months in the SHARP phase 3 randomised trial in patients with advanced hepatocellular carcinoma when compared against placebo control (Llovet, 2007). These results were presented at the annual meeting of the American Association of Clinical Oncology in July 2007.

In November 2007, the US FDA approved Sorafenib for the treatment of advanced primary HCC.

The survival benefit observed for Sorafenib in the SHARP study suggests that it is logical treatment strategy to incorporate both SIRT and Sorafenib – two agents with demonstrated efficacy in the treatment of advanced HCC, but with separate mechanisms of action – into the earlier stages of

treatment of patients with HCC (Senan, 2007). Combining the newest and most effective targeted therapy for HCC with radiotherapy, in the form of SIR-Spheres, is the logical next step in the management of this disease.

Thus, this pilot study has as its primary goal the development of a safe treatment schema for the combination of these two modalities for the first-line treatment of patients with inoperable primary HCC. To that end we seek to gain understanding of the potential advantages and limiting toxicity of combined biologic therapy and liver radiotherapy with SIR-Spheres.

6 PRECLINICAL TESTING OF SIR-SPHERES

6.1 Biocompatibility Safety Data

The following summarises the biocompatibility data for SIR-Spheres on file at Sirtex and with regulators:

(a) FDA in PMA 990065 Vols. 5 and 6

(b) European Union held by BSI in Design Dossier relating to certificate CE 70318

(c) TGA in File DV-2006-3529

As a medical device the toxicity profile of SIR-Spheres is relatively benign. The following tests have been carried out to ensure the safety of the device. These tests, where relevant, have been performed on both labelled and non-radioactive microspheres. Non-irradiated SIR-Spheres have been assessed both in animals and in vitro for the following:

- Haemocompatibility (in vitro) (tested to ISO 10993)
- Mammalian Cell Cytogenicity (in vitro) (Chinese Hamster Ovary Cells)
- Cytotoxicity (in vitro) (tested to ISO 10993 (part 5))
- Bacterial Reverse Mutation Test (in vitro) (OECD 471 & 472)
- Maximum Sensitization (Guinea Pig) (tested to ISO 10993-10)
- Intracutaneous Toxicity (Reactivity) (Rabbit) (tested to ISO 10993-10)
- Systemic Toxicity of Potential Leach Products (Mouse)

In summary, SIR-Spheres are haemocompatible, non-cytotoxic, non-mutagenic, non-toxic locally or systemically and are a mild sensitizer in the guinea pig under the conditions of the test. The details for the tests are presented in greater detail below. All tests were carried out on microspheres labelled with inert yttrium. Radioactive microspheres are cytocidal, hence any potential toxicity of the polymer or yttrium itself is masked.

6.2 Toxicology

All testing was conducted in compliance with GLP in compliance with the OECD Principles of GLP (ISBN 9264-12367-9-1982).

6.2.1 Localised Toxicity

Localized toxicity was assessed with the Intracutaneous Injection Test in the Rabbit (ISO 10993-10, March 1995). This test was conducted with a 50% v/v dilution of the microspheres in water, as the standard presentation will not traverse an intradermal needle. Three female New Zealand white rabbits were used and each rabbit had 5 x 0.2 ml of the test device injected intradermally on one side of the midline of the back and 5 x 0.2 ml of water for injection as the controls on the other side. At the completion of the observation period (72 hours) the primary irritation scores and the primary irritation index were calculated as per ISO 10993-10. There was negligible response to the device indicating that

it is not locally irritant or toxic.

6.2.2 Systemic Toxicity

Systemic toxicity was assessed with the Systemic Injection Test in the Mouse. The methodology was from ISO 10993-11 biological evaluation of medical devices part 11: Tests for systemic toxicity, and also the United States Pharmacopoeia 23 1995 for assessment of biological reactivity, in-vivo, section 88, page 1699. This test was conducted to evaluate systemic responses to extracts of the microspheres following intravenous and intraperitoneal injection.

Polar (water for injection) and non-polar (cottonseed oil) extracts were prepared. Blanks of both extracts were also prepared. A fifth solution (Solution A), being neat supernatant from centrifuged microspheres was also used. The four extract preparations were each tested in five mice, all of which received only a single systemic injection. Solution A was tested in four mice. Doses were all 50 ml/kg.

Animals were observed over a 72 hour period for signs of toxicity. There were no differences between blanks and extracts and all animals in all groups maintained weight and a healthy appearance throughout. Intravenous administration of the water for injection in which the microspheres are supplied failed to produce apparent toxic effects. Under the conditions of this study, SIR-Spheres do not leach or produce any toxic substances that are released systemically.

6.3 Mutagenicity

Mutagenicity was assessed using the Bacterial Reverse Mutation Test utilizing the strains Salmonella typhimurium TA 1535, TA1537, TA 98 and TA 100, and Escherichia coli WP2 uvrA. This test assesses the mutagenicity of a substance by its ability to revert specified bacterial strains from auxotrophic growth to prototrophy. It was conducted according to the requirements of the OECD regulatory guideline for testing chemicals, OECD 471 and 472, adopted May 26th 1983.

Positive controls consisted of direct acting mutagens and those that require metabolic activation. Direct mutagens were sodium azide, 9-aminoacrindine, 2-nitrofluorene and cumene hydroperoxide for S. typhimurium and 4-nitroquinoline-N-oxide for E. coli. The metabolically activated mutagen was 2-aminoanthracene for both bacterial strains. Rat cytochrome P450 mitochondrial fraction was the metabolic activation system used. The methodology involved initially using a plate. If this was positive, the second experiment was also with a plate, but if negative, then pre-incubation would be used. The mean and the standard deviation of the plate counts for each experiment were calculated and statistically assessed using a Dunnett's test. A positive result is a statistically significant increase in the numbers of revertants scored in two separate experiments. A negative result is no greater increases in revertants than may be expected from normal variation for any strain in either experiment.

All positive controls gave results in the expected ranges indicating the strains used were sensitive to mutagens. There were no statistically significant increases in revertants from SIR-Spheres, thus this device is not mutagenic under the conditions of this test.

Mutagenicity was also assessed using an in vitro cytogenetic test, which determines if mutagenicity (if present) is due to structural chromosomal damage. This was performed in mammalian cells (Chinese Hamster Ovary Cells). Mutagenicity after metabolic activation of the test substance was also assessed

by using the rat cytochrome P450 mitochondrial fraction. Positive controls were mitomycin C (direct mutagen) and benzo(a)pyrene and cyclophosphamide were the metabolically activated mutagens.

Scoring of chromosomal damage was by the ISCN classification. Any increase in number of aberrations was compared to negative control using a Fisher's Exact test. The positive controls caused statistically significant increases in aberrations scored, indicating sensitivity of the test system. Under the conditions of this test SIR-Spheres are not clastogenic.

6.4 Cytotoxicity

Cytotoxicity was assessed by an in vitro cytotoxicity test, which assessed the potential cytotoxicity of leachable endogenous or extraneous substances on the microspheres. The cell lines used were mouse fibroblast L929 (ATCC, CCL1, NCTC clone 929). Phenol was the positive control and neat minimum essential medium (MEM) was the negative. Cells were examined microscopically after incubation with dilutions of the supernatant (water for injection) from the microspheres. The dilutions of supernatant used were 0.5%-2% v/v. Under the conditions of this test, the microspheres leached no substance that altered cell morphology or caused any cytotoxic effects at concentrations of 0.5, 1 and 5 mg/ml.

6.5 Haemocompatibility

Haemocompatibility was assessed according to ISO 10993-4 'Selection of Tests for Interactions with Blood'. The positive control was de-ionized water and the negative was normal saline. These results were in the expected ranges. The cell line was human erythrocytes. Solutions of whole blood with supernatant from the microspheres, as well as solutions of microspheres from 0.5 mg/mL to 5 mg/mL were tested. After incubation, the test tubes were centrifuged and assessed spectrophotometrically at 545 nm. Under the conditions of this test, less than 5% haemolysis was considered non-hemolytic. Neither the supernatant from the microspheres or solutions of microspheres were haemolytic. A 5 mg/ml solution of microspheres is approximately iso-osmolar with normal saline.
Under the conditions of this test, any potentially leachable substances in or on the microspheres have no haemolytic activity against human erythrocytes.

6.6 Sensitising Ability

Sensitizing ability was assessed with the maximum sensitization test in the guinea pig. This test evaluates the potential of the device to cause a delayed dermal hypersensitivity/ Type 1V immune response. This test was conducted using the methodology of ISO 10993-10 Biological Evaluation of Medical Devices: Test for Irritation and Sensitization of March 1995.

The test was conducted on 20 test female albino guinea pigs and 10 controls. The topical range finding study in four animals indicated that the microspheres were non-irritant. The lack of primary irritancy allowed assessment for delayed sensitivity. Of those tested, three of the 20 animals gave a positive skin response (grade 1) at 24 or 48 hours after challenge. No animals in the test or control group exhibited a positive reaction to water. The weak positive responses in the test group indicate a delayed dermal hypersensitivity according to criteria in ISO 10993. The device is therefore considered a mild sensitizer under the condition of this test.

6.7 Summary

These test results are evidence of the inert nature of the microspheres. The therapeutic activity of the device is due to the emission of beta radiation. The polymer or the yttrium itself does not contribute to the cell death expected from implantation of the microspheres. The device, once decayed, causes no toxicity when left in situ within treated liver tumours. The implications of the mild sensitizing ability of the device to humans are difficult to determine. Relatively few patients receive subsequent exposure to the microspheres and extrapolation of dermal sensitivity to liver vasculature is problematic. Clinical experience to date has not demonstrated a sensitivity reaction to SIR-Spheres.

As SIR-Spheres are a device that is implanted once in most patients, dose interval does not apply. In some patients, the activity may be implanted into each half of the liver separately with an interval of several weeks. In some cases this may reduce the potential toxicity. This is still considered a single treatment. It is conceivable that the implant of the microspheres may be repeated in some patients, if there is reason to believe that repeated radiation therapy would be beneficial. To date, relatively few patients (less than 5%) have received repeated implants of SIR-Spheres. Multiple treatments are generally injected in the order of 6 - 9 months apart. The primary risk with repeated implants is potential toxicity from cumulative radiation exposure, rather than potential toxicity from the microsphere components.

7 CLINICAL RISK ANALYSIS

7.1 Clinical Rationale

7.1.1 Complications and Toxic Effects of SIR-Spheres

Overall, the incidence of complications after SIR-Spheres therapy in broader clinical use, if patients are selected appropriately and target (i.e. liver) delivery is performed meticulously, is low.

Gastrointestinal complications occur in less than 10% of those treated (Kennedy, 2006; Stubbs, 2004) and are largely preventable. Gastric and duodenal ulceration have been reported after SIRT and are related to the inadvertent intestinal deposition of microspheres via extra-hepatic visceral arterial branches. Even in the absence of extra-hepatic activity on Tc-99 labelled MAA and Bremsstrahlung emission images, gastrointestinal symptoms have been reported to develop. The risk of gastrointestinal ulceration can be minimized via the routine coil embolisation of the extra-hepatic visceral arteries (e.g. gastro-duodenal, right gastric, supraduodenal arteries) before infusion of SIR-Spheres (Liu, 2005). Similar gastrointestinal complications have been observed after treatment with transcatheter arterial chemoembolisation (TACE) (Hirakawa, 1988) and hepatic artery pump infusion (Kemeny, 1984).

The gallbladder also may receive SIR-Spheres through a patent cystic artery, leading to radiation cholecystitis. In order to avoid this potential complication infusion distal to the cystic artery may be possible. However, even with infusion of SIR-Spheres proximal to the cystic artery, the risk of radiation cholecystitis requiring cholecystectomy is low (Liu, 2005). This issue is addressed at the time of administration by the treating Interventional Radiologist, via catheter placement and/or selective embolisation/optimization of the hepatic arterial vasculature.

A life-threatening complication, progressive pulmonary insufficiency secondary to radiation-induced lung fibrosis can be avoided by excluding from treatment with SIRT any patient with significant liver-to-lung shunting (Leung, 1995). There have been no reported occurrences of radiation induced lung disease since routine pre-treatment lung shunt quantification using Tc-99 labelled MAA has been standard practice.

Radiation induced liver disease (RILD) is a rare complication of SIRT treatment. It results in various degrees of hepatic decompensation and is clinically indistinguishable from hepatic veno-occlusive disease. RILD is manifested clinically by the development of anicteric ascites. High doses of corticosteroids typically are administered in an attempt to decrease intra-hepatic inflammation. Treatment results are variable and mostly of minimal benefit, as the condition will progress in some patients to hepatic insufficiency of various degrees.

Pancytopaenia as a result of bone marrow suppression from leaching of yttrium-90 was reported after the use of the earliest microsphere device (Mantravadi, 1982). The yttrium-90 microsphere device has subsequently undergone multiple revisions and this complication has not been reported since that time.

From the total experience with SIR-Spheres, major complications[1] have included:

- In approximately one-third of patients, administration of SIRT causes immediate short term abdominal pain requiring narcotic analgesia and is typically self-limiting.
- Post-SIRT lethargy and nausea are common symptoms and can last up to two weeks and may require medication.
- Most patients develop a mild-moderate fever that may last for several days following SIRT administration. This fever does not usually require treatment.
- The most common potential serious complications result from either (i) inadvertent administration of SIR-Spheres into the gastrointestinal tract resulting in gastritis/duodenitis or (ii) radiation induced liver disease resulting from a radiation overdose to the normal liver parenchyma. The incidence of gastritis/duodenitis can be reduced by meticulous attention to the administration procedure so as to ensure that there is a minimal chance of SIR-Spheres entering the numerous small arteries supplying the gastrointestinal tract (Salem 2006; Liu 2005). Radiation induced liver disease is largely, but not totally, preventable by using appropriate SIRT doses and making allowances for dose reduction when there is increased risk of causing radiation damage such as in pre-existing liver damage, poor liver reserve or small volume tumour mass in the liver. The reported incidence of gastritis/duodenitis is <10%, while the reported rate of radiation induced liver disease is < 1%.
- Rare complications that have been reported include acute pancreatitis resulting from SIR-Spheres refluxing in the hepatic artery and lodging in the pancreas, and liver abscess from infection of necrotic tumour.
- Previously reported radiation pneumonitis has not been observed where appropriate pre-treatment workup and dose reductions are followed.
- There is some evidence that there is a decrease in leukocyte levels, with a nadir 8 weeks after implantation. This has been evident in both first line and refractory studies with studies reporting a median number of leukocytes of 3.55×10^9/L (NCIC CTC3 grade 1). Leukocyte levels usually recover from this point, with the median value rising to normal levels 4 - 8 weeks after the decline.

The rate of treatment related complications has been shown to run at 2 – 10%, with outcomes related to the skill and experience of the Interventional Radiologist and Authorized User.

---

1 Unpublished data, Sirtex Technology Pty Ltd

7.1.2 Identifying the Specific Risks of SIR-Spheres

It is established that SIR-Spheres are contraindicated (Sirtex Training Manual, Sirtex Medical Inc.) in patients who have:

- Had previous external beam radiation therapy to the liver.
- Ascites or other clinical signs of liver failure.
- Abnormal synthetic and excretory liver function tests as determined by serum albumin (must be > 3.0 g/dL) and total bilirubin (must < 2.0 mg/dL), respectively.
- Complete main portal vein thrombosis without cavernous transformation.
- Disseminated extra-hepatic disease.
- Tumours amenable to surgical resection or ablation with intent to cure.
- Greater than 20% lung shunting (as determined by pre-treatment Tc^{99} labelled MAA nuclear medicine lung shunt study).
- Pre-assessment angiogram and MAA nuclear medicine scan demonstrating significant and uncorrectable activity in the stomach, pancreas or bowel.
- Been treated with Capecitabine within the previous 8 weeks, or who will be treated with Capecitabine within 8 weeks of treatment with SIR-Spheres.

The risks that could result from treating patients possessing the aforementioned contraindications will be mitigated fully by excluding these patients from treatment with SIR-Spheres.

Whether a patient is amenable to surgical resection or has disseminated or extra-hepatic disease is typically determined using established cancer staging investigations (triple phase contrast angio-portal CT scan, MRI, PET imaging) and comprehensive case review within an experienced multi-disciplinary team setting.

The treatment of unresectable HCC with SIR-Spheres must be based on specific findings on cross-sectional images and angiograms in each patient. The initial work-up of patients being considered for SIRT treatment must include CT or MRI imaging of the liver for assessment of tumour and normal liver volumes, portal vein patency and the presence of extra-hepatic disease, together with detailed hepatic and visceral angiography.

The aim of pre-treatment assessment of the hepatic arterial vasculature is to ensure delivery of SIR-Spheres to the tumour target. The superior mesenteric, celiac and hepatic arterial branches are evaluated. Evaluation includes a determination of the arterial location and any consequent necessity for embolisation of the gastroduodenal artery, right gastric artery and any other arteries to prevent non-target administration of SIR-Spheres into the gastrointestinal tract, pancreas or other abdominal organs.

The presence of variant hepatic arterial anatomy may alter the treatment plan. Treatment with SIRT is precluded by stenosis or slow antegrade flow within the hepatic arteries that results in embolic occlusion of the vessel and therefore, reflux into extra-hepatic territories. Complete portal vein thrombosis with absence of hepatopedal flow may indicate that the patient has a high risk for ischemic complications.

Hepatopulmonary shunting secondary to tumour-related pathologic arteriovenous pathways, as well as reflux toward the gastrointestinal region, may be detected as scintigraphy with the injection of 180 – 220 MBq of Tc-99 labelled MAA as a SIRT surrogate into the hepatic arterial territory. The hepatopulmonary shunt fraction is then calculated as the ratio of the gamma emission count in the lung to that in the liver in regions of interest in planar scintigrams. The ratio is calculated as a percentage that is rounded to the nearest whole percentage point.

Patients in whom the hepatopulmonary shunt fraction is greater than 20% of the injected dose or in whom the shunt fraction indicates potential exposure to the lung to an absorbed radiation dose of more than 30 Gray should be excluded from treatment with SIRT.

Study subjects will be selected from the population of patients with unresectable HCC referred for treatment at the participating institutions in Singapore. The racial, gender and ethnic characteristics of the proposed patient population will reflect the demographics of the respective referral areas of each of the participating institution(s).

7.1.3 Identifying the Risks of Combining SIR-Spheres With Sorafenib

Previous studies documenting the combined use of SIR-Spheres and contemporary systemic chemotherapy have indicated that when used in combination with either 5-FU/LV, or with Irinotecan that there has been no detectable increase in adverse events in comparison with SIRT alone (van Hazel, 2007; van Hazel, 2005).

Concomitant use of SIR-Spheres and Oxaliplatin (as part of a FOLFOX regimen), as demonstrated in a 20 patient phase 1 dose-escalation study, has resulted in a slight increase in neutropaenia in the chemotherapy cycles immediately following SIRT treatment (Sharma, 2007). This decrease was transient, resolving with a 1-week delay in the administration of further chemotherapy. Besides the early neutropaenia, the rate of adverse events reported in this study was similar to those normally reported in studies of FOLFOX chemotherapy alone.

While preclinical studies have demonstrated enhanced radiation-induced cell kill when anti-angiogenesis therapy is combined with radiotherapy (Li, 2005; Hess, 2001; Lee, 2000), the optimal biological doses of anti-angiogenic agents, including Sorafenib, to be used in combination with radiotherapy are unknown (Senan, 2007).

Although Sorafenib is generally well tolerated, some serious adverse events have occurred in some patients treated with Sorafenib in clinical trials, including

Hypersensitivity and known adverse drug reactions to sorafenib may include:

- Diarrhea
- Hand-foot syndrome
- Fatigue
- Rashes
- Anorexia

• Hypertension

The hand and foot symptoms may be resolved with discontinuation of sorafenib. Symptoms can sometimes be successfully treated with non-steroidal anti-inflammatory agents and urea-containing cream. This is not a complete list of adverse events. The known side effect profile of sorafenib can be found in the current version of the IB. The IB is updated on a regular basis.

Both men and women enrolled in the study must use adequate barrier birth control measures during the course of the trial. As with any new chemical entity, there is always potential for unexpected adverse events including hypersensitivity reactions. Sorafenib has the ability to inhibit a variety of liver metabolic enzymes in vitro. The clinical impact of this inhibition in humans taking drugs metabolized by these enzymes is unknown.

Therefore, all patients enrolled onto this trial who are taking concomitant medications that are known to be metabolized by the liver should be closely observed for side effects of these concomitant medications. Furthermore, patients taking narrow therapeutic index medications including: warfarin, phenytoin, quinidine, carbamazepine, phenobarbital and cyclosporine and digoxin should be monitored proactively. The use of ritonavir and St. John's Wort is not allowed during the study treatment period due to possible drug interaction. Therapeutic monitoring should be performed consistent with the local clinical standard of care following dose selection or modification of the agents. In general, patients should be closely monitored for side effects of all concomitant medications regardless of path of elimination.

Sorafenib is at least partially metabolized by the CYP 3A4 enzyme in the liver. The possible effect that inhibitors of CYP 3A4 may have on sorafenib is still being explored. Preliminary data from a clinical study indicated that there is no impact of ketoconazole co-administration on sorafenib PK. Drugs including ketoconazole, itraconazole, fluconazole and ritonavir are therefore not likely to affect sorafenib PK or safety.

This is the first clinical trial designed to assess the safety of SIR-Spheres when used in combination with Sorafenib.

In order to mitigate risks, only Interventional Radiologists with extensive expertise (defined as a minimum of 20 uncomplicated patient treatments using SIR-Spheres) in the administration of SIR-Spheres will participate on this study. Extensive expertise is required in order to 1) meticulously perform the visceral angiograms and reliably identify any aberrant hepatic vessels which may be present and which supply the GI tract; and 2) possess the necessary technical expertise to prevent microsphere delivery to these aberrant vessels, viz. embolisation or distal microsphere injection.

To further mitigate the possible risks, each patient enrolled to this pilot study will undergo routine closure of the hepatic arterial branches which may allow inadvertent delivery of microspheres to the GI tract (gastro-duodenal artery, right gastric artery, supra-duodenal artery, retro-duodenal artery etc.). These techniques are well described in the Interventional Radiology literature (Liu, 2005).

With respect to the adverse reactions reported from Sorafenib treatment it is not expected that any of these risks are potentiated by treatment with SIR-Spheres.

8 OBJECTIVES

This study will evaluate the safety and activity of a regimen of SIR-Spheres in combination with Sorafenib for the treatment of patients with inoperable primary HCC.

The primary objective of this study is:

- Toxicity and safety.
- Tumour response rate (liver ± any site).

The secondary objectives of this study are:

- Progression free survival at any site.
- Progression free survival in the liver.
- Survival.
- Hepatic and extra-hepatic recurrence rate.
- Quality of life.
- Rate of downstaging to surgical resection or ablative therapy.

Patients will be followed until death wherever possible in the evaluation of the primary and secondary objectives of this study.

9 DESIGN OF THE CLINICAL STUDY

This study is an Investigator-Initiated multi-centre study conducted by the Asia Pacific Hepatocellular Carcinoma Trials Group that will assess the safety and effectiveness of a regimen of SIR-Spheres in combination with Sorafenib for the treatment of patients with inoperable primary HCC. The study will recruit a maximum of 31 patients.

The study is structured so that patients with unresectable primary HCC who satisfy the study eligibility criteria, will receive:

- A single injection of SIR-Spheres into the liver delivered together with Sorafenib therapy, commencing 14 days after (for the first patient cohort) or 11 days after (for the second patient cohort) the delivery of SIR-Spheres.

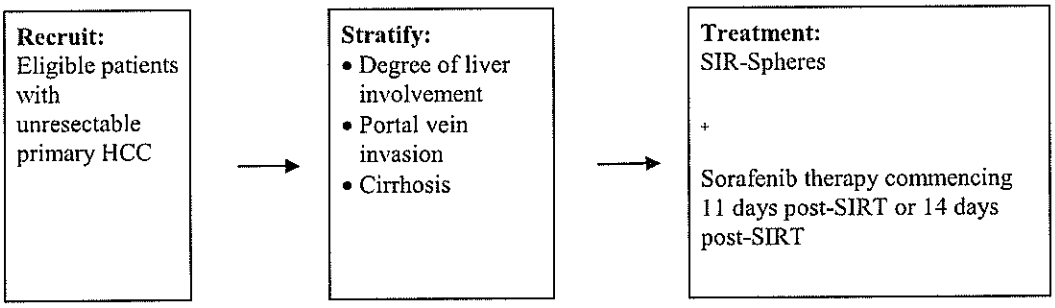

Protocol Treatment: SIR-Spheres
SIR-Spheres will be administered at the calculated (patient-specific) activity, described in Section 12: Treatment.

Protocol Treatment: Sorafenib
Sorafenib therapy will commence 14 days after (for the first patient cohort) or 11 days after (for the second patient cohort) the delivery of SIR-Spheres:

Sorafenib 400mg orally, twice daily.

Continued until: evidence of treatment failure (lack of efficacy resulting in disease progression); or unacceptable toxicity occurs.

9.1 Patient Eligibility

Patients must have an unequivocal diagnosis of primary HCC that cannot be managed via potentially curative treatment modalities, such as surgical resection, liver transplantation or percutaneous ablation. Diagnosis may be based on:

a) Histology consistent with HCC and its histological variants such as poorly differentiated HCC and sacomatoid HCC **or**

b) Space occupying lesion of the liver demonstrated by ultrasound, CT scan (non-dynamic) or MRI (non-dynamic) **and either:**
   - Serum alpha-feto protein level of at least 400 mcg/L **or**

c) Radiological evidence of HCC by dynamic contrast-enhanced CT scan or dynamic contrast-enhanced MRI*
   and serology positive for Hepatitis B or C
   and alpha-feto protein above normal range

   **Radiological criteria on dynamic scan*
   Enhancement in the hepatic arterial phase
   Hypodensity on portal venous phase

Study entry is defined as the date that the informed consent document is signed by the patient. No patient may enter the study without signing the informed consent document.

The screening period is defined as the time period between study entry and the commencement of protocol treatment and is not to exceed 28 days. All screening tests required to confirm that a patient meets the eligibility criteria must be completed during the allowed 28 day screening period.

When a patient is entered into the study, a Subject Identification Number (SIN) will be assigned to the patient. The first 3 digits of the SIN denote the investigational site and the second 3 digits denote the subject at that investigational site. The SIN will be used on all of that patient's Case Report Forms (CRFs) and will be unique to that patient.

In order to be considered eligible for the study and to receive study treatment, patients must fulfil the inclusion and exclusion criteria specified in 10.2 and 10.3 below.

9.2 Inclusion Criteria

Patients must fulfil all of the following criteria in order to be eligible for this study:

(a) Unresectable HCC with or without systemic metastases.

(b) Willing, able and mentally competent to provide written informed consent prior to any testing under this study protocol, including screening tests and evaluations that are not considered to be part of the subject's routine care.

(c) Aged 18 years or older of either gender and any race, religion or socioeconomic group.

(d) Unequivocal diagnosis of primary HCC (as defined above)

(e) HCC that is not amenable to surgical resection or immediate liver transplantation, or that is not optimally treatable with local ablative techniques such as radio-frequency ablation, consistent with the practice of the clinical trial centre.

(f) Measurable disease, defined as at least one lesion that can be accurately measured in at least one dimension (longest diameter to be recorded) as ≥10 mm with spiral CT scan.

(g) ECOG performance status 0 – 1.

(h) Adequate haematological, renal and hepatic function as follows:

Leukocytes	≥ 2,500/µL
Absolute Neutrophil Count	≥ 1,500/µL
Platelets	≥ 50,000/µL
Haemoglobin	> 9.5 g/dL
Total bilirubin	< 2.0 mg/dL (SIR-Spheres should not be administered as a whole liver treatment if the total bilirubin is ≥ 2 x the institutional upper limit of normal).
INR	≤ 2.0
ALP	≤ 5 x institutional upper limit of normal
AST / ALT	≤ 5 x institutional upper limit of normal
Albumin	≥ 2.5 g/dL
Creatinine	≤ 2.0 mg/dL

The blood results must be less than 29 days old at the time of confirming patient eligibility to receive protocol treatment.

(i) Life expectancy of at least 3 months without any active treatment. This is defined as a patient who has OKUDA I and II inoperable HCC.

(j) Suitable for protocol treatment as determined by clinical assessment undertaken by the Investigator.

(k) Female patients must be either postmenopausal or, if premenopausal, must have a negative pregnancy test and agree to use two forms of contraception if sexually active during their study participation.

(l) Male patients must be surgically sterile, or if sexually active and having a pre-menopausal female partner then must be using an acceptable form of contraception.

(m) Hepatic arterial anatomy suitable for implantation of SIR-Spheres, as assessed by hepatic angiogram.

(n) Lung shunt fraction less than or equal to 20% as assessed by a Tc-99m macroaggregated albumin liver to lung breakthrough scan.

9.3 Exclusion Criteria

The exclusion criteria include both the listed contraindications to SIR-Spheres and the exclusion criteria specific to the setting of this investigational study.

9.3.1 Contraindications to SIR-Spheres

SIR-Spheres have no therapeutic effect on extrahepatic disease and this must be taken into account when considering treatment. There is no data on the safety of SIR-Spheres in pregnant women or children. Although less demanding on the patient's physical resources than some other treatments for liver cancer, it should be recognised that implantation of SIR-Spheres is a significant intervention and this must be taken into account when the decision to treat is made. SIR-Spheres are contraindicated in patients who have (SIR-Spheres Package Insert, Appendix A):

(a) Had previous external beam radiation therapy to the liver.

(b) Any ascites or other clinical signs of liver failure, on physical examination.

(c) Abnormal synthetic and excretory liver function tests (LFTs) as determined by serum albumin (must be ≤ 2.5 g/dL) and total bilirubin (must be > 2.0 mg/dL), respectively.

(d) Tumours amenable to surgical resection for cure at presentation.

(e) Greater than 20% lung shunting of the hepatic artery blood flow determined by Tc-99 MAA scan.

(f) Pre-assessment angiogram and Tc-99 MAA scan that demonstrates significant and uncorrectable activity in the stomach, pancreas or bowel.

(g) Been treated with Capecitabine within the previous 8 weeks, or who will be treated with Capecitabine within 8 weeks of treatment with SIR-Spheres, due to the possible risk of potentiating or causing liver dysfunction.

(h) Complete main portal vein thrombosis.

9.3.2 Exclusion Criteria Specific to This Investigational Study

(a) Subjects who have had hepatic artery directed therapy within the previous 3 months.

(b) Subjects who have had intravenous chemotherapy within the previous 4 weeks or those who have not recovered from adverse events due to agents administered more than 6 weeks previously.

(c) Prior external hepatic radiation therapy for HCC, more than two prior systemic chemotherapy regimes for HCC or any other concomitant therapy for HCC or any investigational agent planned while on this protocol.

(d) Currently receiving any other investigational agents for the treatment of their cancer.

(e) Any other concurrent malignancy, except for adequately treated basal cell or squamous cell skin cancer, *in situ* cervical cancer, or other cancer for which the patient has been disease-free for at least five years.

(f) Presence of clinical signs of CNS metastases due to their poor prognosis and because progressive neurologic dysfunction would confound the evaluation of neurologic and other adverse events.

(g) Uncontrolled intercurrent illness including, but not limited to, ongoing or active infection (except viral hepatitis), symptomatic congestive heart failure, unstable angina pectoris, cardiac arrhythmia, or psychiatric illness/social situations that would limit compliance with study requirements.

(h) Any of the following contraindications to angiography and selective visceral catheterization:
- Bleeding diathesis, not correctable by the standard forms of therapy.
- Severe peripheral vascular disease that would preclude arterial catheterization.
- Portal hypertension with hepatofugal flow as documented on baseline spiral CT scan.

(i) History of allergic reactions attributed to compounds of similar chemical or biologic composition to SIR-Spheres.

(j) Inability or unwillingness to understand or sign a written informed consent document (non English-speaking patients may use an interpreter).

(k) Female subjects who are pregnant or currently breastfeeding.

(l) For female subjects, unless postmenopausal or surgically sterile, unwillingness to practice effective contraception, as defined by the Investigator, during the study. The rhythm method is not to be used as the sole method of contraception.

(m) For male subjects, unwillingness to practice effective contraception (as defined by the Investigator) while taking part in this study, because the effect of the SIR-Spheres treatment on sperm or upon the development of an unborn child are unknown.

(n) Current enrolment in any other investigational drug or device study.

10 STUDY ENTRY

Study entry is defined as the date that the **informed consent document** is signed by the patient. No patient may undergo screening to assess their eligibility to receive protocol treatment, or commence protocol treatment, prior to signing the informed consent document.

10.1 Patient Screening

All patients referred for possible participation in this study must be **screened** by the Investigator to confirm the patient's **eligibility** to receive protocol treatment (see Section 11).

The **screening period**, during which the patient's eligibility to receive protocol treatment as part of this study will be confirmed, is defined as the time period between **study entry** and the commencement of protocol treatment, and is **not to exceed 28 days**. Patients may only commence protocol treatment after all eligibility criteria have been confirmed.

All documentation supporting the inclusion and exclusion criteria and screening investigation results are to be retained by the Investigator.

All patients deemed ineligible to receive protocol treatment will have their initials recorded on the **Patient Screening Log** and no further data will be collected for these patients. The Patient Screening Log will include the reason(s) for the patient's exclusion from receiving protocol treatment and will be maintained by the site.

10.2 Clinical Assessment

All patients must be assessed clinically by the Investigator to determine the patient's eligibility to receive protocol treatment. Clinical assessment includes a comprehensive medical history (including quality of life assessment; see Appendix 9) and physical examination and must be completed within 28 days of study entry.

10.3 Haematological and Serological Assessment

All patients are required to undergo the following haematological and serological investigations in order to determine their eligibility to receive study treatment. These investigations must be completed within 28 days of study entry:

Haematological	Complete blood count
Renal	Electrolytes, urea, creatinine
Hepatic	Liver function tests (AST/ALT, ALP, bilirubin, albumin)
Tumour marker	Serum AFP
Pregnancy test	Serum or urine pregnancy test

10.4 Radiological Assessment

All patients are required to undergo the following radiological investigations in order to determine their eligibility to receive protocol treatment, and to demonstrate the extent of hepatic and extra-hepatic metastases.

10.4.1 CT Scan of the Liver and Abdomen/Pelvis

A multi-phase (at least three-phase) contrast-enhanced spiral CT scan of the abdomen/pelvis to determine the extent of liver disease and to determine the presence and extent of intra-peritoneal extra-hepatic metastases. MRI is acceptable in lieu of CT especially for patients with contrast allergy that prevents CT imaging.

10.4.2 CT Scan of the Chest

A thoracic CT scan to determine the presence or absence of lung metastases.

Each of these CT series must be completed within 28 days of study entry.

10.5 Assessment of Patient Suitability for Selective Internal Radiation Therapy

All patients must be assessed in order to determine their eligibility to receive protocol SIRT therapy. This assessment must be completed within 28 days of study entry.

10.5.1 Hepatic Angiogram

All patients must undergo an outpatient diagnostic hepatic angiogram to determine the vascular anatomy of the liver and to perform a nuclear medicine liver to lung shunt study.

The hepatic angiogram will provide a road map of the arterial supply of the liver in order to plan the optimal delivery of the SIR-Spheres (see Appendix 5). The hepatic angiogram should be performed together with the lung shunt study and the results of these two assessments must be available prior to implanting the SIR-Spheres.

The diagnostic hepatic angiogram must be performed in order to:

- Fully identify and define all relevant hepatic arterial vasculature:
    - Aortogram
    - Superior mesenteric artery (SMA)
    - Celiac axis
    - Left gastric artery (LGA)
    - Common hepatic artery (CHA)
    - Gastroduodenal artery (GDA)
    - Proper hepatic artery (PHA)
    - Right gastric artery (RGA)
    - Left hepatic artery (LHA)
    - Right hepatic artery (RHA)
    - Supraduodenal artery, retroduodenal artery, inferior phrenic arteries

- Cystic artery
- Replaced, accessory and aberrant arteries.

- Confirm the ability to selectively catheterize the hepatic arterial vasculature.

- Assess the flow characteristics in the hepatic arteries.

- Determine the hepatic arterial supply to the tumour(s) i.e. right, left, middle hepatic arteries; replaced, accessory hepatic arteries; extra-hepatic arteries; other aberrant arteries.

- Determine the influence of hepatic arterial anatomy relative to the tumour(s)' distribution on the ability to treat the entire diseased portion of the liver as a whole liver treatment at one setting.

- Confirm the absence of uncorrectable blood shunting from the liver to the gastrointestinal tract or other abdominal organs (e.g. pancreas). If the hepatic angiogram indicates an uncorrectable risk of flow to any of the gastrointestinal organs, then SIRT treatment will not be administered.

- Perform a technetium-99 macro-aggregated albumin (Tc-99 MAA) lung shunt study to assess the presence and degree of lung shunting from the liver.

10.5.2 Tc-99 MAA Lung Shunt Study

In approximately 10-15% of patients with primary HCC there will be sufficient arterio-venous shunts present in the liver to allow more than 20% of the SIR-Spheres injected into the liver to pass through the liver and lodge in the lungs. As excessive liver to lung shunting may result in radiation damage to the lungs, a nuclear medicine 'break-through' scan must be performed in all patients to quantify the extent of liver to lung shunting. See Appendix 6 for details of the technique for performing the Tc-99 MAA lung shunt study.

The percentage of Tc-99 MAA that has escaped through the liver and lodged in the lungs may be expressed as a 'percentage lung shunt'. Normally this is less than 10%. The total lung radiation dose delivered by SIR-Spheres must be kept below 30 Gray in order to ensure that the patient does not develop radiation induced lung disease.

Table 10.5 shows the approximate lung radiation dose delivered for different combinations of 1) SIR-Spheres implanted activity and 2) percentage lung shunting. The table assumes that the mass of both lungs plus blood is 1000 grams.

Table 10.5 Lung radiation dose calculation (Gray delivered to the lungs)

SIR-Spheres activity implanted (GBq)	Lung shunting		
	10 %	15 %	20 %
1.0	5	7.5	10
1.5	7.5	11.25	15
2.0	10	15	20
2.5	12.5	18.75	25
3.0	15	22.5	30

10.6 Commencement of Treatment

Once patients have been screened and deemed eligible to participate in the study, protocol treatment may commence.

11 TREATMENT

Patients should begin study treatment as soon as possible, and not later than 28 days after study entry. All patients will have follow up every 12 weeks after the End of Study. Patients who had gone for Surgical Resection/ RFA (as a result of disease down-sizing) are to be followed for 5 years, otherwise, all other patients are to be followed for 18 months or until death, whichever occurs earlier.

11.1 Protocol Treatment: SIR-Spheres Therapy

SIR-Spheres will be administered at the calculated (patient-specific) activity, described in Section 11.1.3: Administration of SIR-Spheres

Summary: First patient cohort:

Day:	1	2	3	4	5	6	7	8	9	10	11	12	13	14	15	16	17	18	19	20	21	22	23	24	25	26	27	28	29	30	31
	SI													S	S	S	S	S	S	S	S	S	S	S	S	S	S	S	S	S	S

Summary: Second patient cohort:

Day:	1	2	3	4	5	6	7	8	9	10	11	12	13	14	15	16	17	18	19	20	21	22	23	24	25	26	27	28	29	30	31
	SI										S	S	S	S	S	S	S	S	S	S	S	S	S	S	S	S	S	S	S	S	S

Note: SI = SIRT; S = Sorafenib.

11.1.1 Calculation of SIR-Spheres Activity to Deliver Using the Partition Model

The therapeutic activity of SIR-Spheres to deliver into the hepatic arterial circulation will be calculated using the **Partition Model** of Ho *et al*, and upper limits of radiation absorbed dose to the normal liver and lungs. The therapeutic activity of SIR-Spheres to administer for any one treatment will be capped at a maximum of 3.0 GBq.

The general equation for estimating the radiation absorbed dose, D, in tissue from ^{90}Y is given by:

$$D = S \times A / M \qquad (1)$$

where

S = 49670 Gy-g/GBq (mean energy deposited per unit of ^{90}Y activity)
A = activity administered to the tissue (GBq)
M = tissue mass (g)

Conversely, the activity to administer given a target radiation absorbed dose is given by:

$$A = D \times M / S \qquad (2)$$

Total Administered Activity

The total therapeutic activity administered is the sum of that in the tumour, normal liver and lung (due

to shunting) masses:

$$A_{total} = A_{tumour} + A_{liver} + A_{lung} \qquad (3)$$

Lung Shunt Calculation
The lung activity can be expressed in terms of the total activity as:

$$A_{lung} = A_{total} \times (L/100) \qquad (4)$$

where L is the percent lung shunting. Percent lung shunting will be estimated from the pre-therapy anterior and posterior planar technetium-99m MAA images of the liver and lung fields (see Appendix 5). Regions of interest will be defined over the total lungs and total liver (including tumours), and the percent lung shunting calculated as the ratio of total lung geometric mean counts to the sum of total lung plus total liver counts:

$$L = 100 \times [N_{lungA} \times N_{lungP}]^{1/2} / ([N_{lungA} \times N_{lungP}]^{1/2} + [N_{liverA} \times N_{liverP}]^{1/2}) \quad (5)$$

where:

N_{lungA} and N_{lungP} are the anterior and posterior total lung region counts
N_{liverA} and N_{liverP} are the anterior and posterior total liver region counts

Lung Dose-Limited Administered Activity
The maximum tolerated activity based on the upper limit of radiation absorbed dose in the lungs, D_{lung}, will be calculated using Eq. 4 above:

$$A_{totalLung} = (D_{lung} \times M_{lung} / 49670) \times (100 / L) \qquad (6)$$

where:

$(D_{lung} \times M_{lung} / 49670) = A_{lung}$ using Eq. 2 above
D_{lung} = maximum tolerated lung dose (25 Gy)
M_{lung} = mass of lung tissue (estimated to be 1000 g)

Normal Liver Dose-Limited Administered Activity
The maximum tolerated activity, $A_{total\ Liver}$, based on an upper limit of radiation absorbed dose in normal liver, D_{liver}, will be calculated using Eq. 3 and substitution for A_{tumour}, A_{liver} and A_{lung}.

An average tumour-to-normal uptake ratio, R_{avg}, will be calculated, with individual values calculated from tumour and adjacent normal liver region average counts per pixel, C, in a central transaxial slice through up to five (5) tumours, using an attenuation corrected ^{99m}Tc MAA SPECT scan (See Appendix 5). Counts per pixel is assumed to be directly proportional to activity per unit mass (a reasonable assumption for an attenuation corrected SPECT exam):

$$R = C_{tumour} / C_{liver} = [A_{tumour} / M_{tumour}] / [A_{liver} / M_{liver}] \qquad (7)$$

Normal liver mass will be calculated from a CT estimate of total (normal + tumour) liver mass, M_{total}, and tumour mass, M_{tumour} (see Appendix 5):

$$M_{liver} = M_{total} - M_{tumour}$$

Substituting for A_{tumour} (= $R_{avg} \times A_{liver} \times [M_{tumour} / M_{liver}]$) and A_{lung} (Eq. 4), Eq. 3 becomes:

$$A_{total\ Liver} = A_{liver} \times (R_{avg} \times [M_{tumour} / M_{liver}] + 1) + A_{total\ Liver} \times (L/100)$$
or
$$A_{total\ Liver} = A_{liver} \times (R_{avg} \times [M_{tumour} / M_{liver}] + 1) / (1 - [L/100])$$

and substituting for A_{liver} using Eq. 4, Eq. 3 becomes:

$$A_{total\ Liver} = D_{liver} \times ([R_{avg} \times M_{tumour}] + M_{liver}) / [\ 49670 \times (1 - [L/100])\ ] \quad (8)$$

where:

D_{liver} = 80 Gy maximum tolerated normal liver dose (70 Gy in patients with cirrhosis)

Final Dose-Limited Administered Activity

The lower of the calculated lung dose-limited and normal liver dose-limited maximum tolerated activities, MIN($A_{total\ Lung}$, $A_{total\ Liver}$), will be used as the administered therapeutic activity. If $A_{total\ Lung} < A_{total\ Liver}$, then:

$$D_{liver} = A_{totalLung} \times [\ 49670 \times (1 - [L/100])\ ] / ([R_{avg} \times M_{tumour}] + M_{liver}) \quad (9)$$

Note, however, that as stated in Section 11.1.1 "Calculation of SIR-Spheres Activity of Deliver", the therapeutic activity of SIR-Spheres to administer for any one treatment will be capped at a maximum of 3.0 GBq.

Estimated Tumour Radiation Absorbed Dose

The expected dose (in Gy) in the tumour mass may be estimated from the total activity to be administered, A_{total} (equal to the smaller of $A_{total\ Lung}$ or $A_{total\ Liver}$), and Eqs. 3, 4, 7 and 2:

$$A_{tumour} = A_{total} - A_{lung} - A_{liver}$$
$$A_{tumour} = A_{total} \times (1 - [L/100]) - A_{liver}$$
$$A_{tumour} = A_{total} \times (1 - [L/100]) - A_{tumour} \times (M_{liver} / [R_{avg} \times M_{tumour}])$$
$$A_{tumour} = A_{total} \times (1 - [L/100]) / [1 + (M_{liver} / [R_{avg} \times M_{tumour}])] \quad (10)$$

$$D_{tumour} = 49670 \times A_{tumour} / M_{tumour} \quad (11)$$

11.1.2 Calculation of SIR-Spheres Activity to Deliver Using the Body Surface Area

In practice the **Partition Model** can only be applied when the tumour mass is localised in a discrete area within the liver and the tumour can be drawn as an "area-of-interest" on a SPECT camera image. This is usually possible for patients with primary HCC where there is commonly a large single tumour mass.

However, for patients enrolled in this study who do not have a single tumour mass but instead have multiple areas of tumour involvement that preclude drawing "areas-of-interest" that define the tumour and normal parenchyma compartments, the **Partition Model** cannot be used. In this case, the **Body Surface Area** (BSA) method for calculating the therapeutic radioactivity to administer will be used.

The activity of SIR-Spheres to deliver into the hepatic arterial circulation using the BSA method is calculated using the tables in Appendix 7.

The percentage tumour involvement of the liver is determined from the screening (baseline) triple phase contrast-enhanced CT scan of the liver. The percentage tumour involvement must be recorded in the patient file and is to be used as the basis for calculating the activity of SIR-Spheres to implant.

11.1.3 Administration of SIR-Spheres

SIR-Spheres will be implanted via a transfemoral hepatic arterial catheter. The activity of SIR-Spheres to deliver into the hepatic arterial circulation is calculated using the tables in Appendix 7. The details of the SIR-Spheres implanted activity will be recorded in the CRF.

The pre-determined end-points for the administration of SIR-Spheres into the hepatic arterial circulation are:

1) Administration of the prescribed activity of SIR-Spheres (as calculated from using the **Partition Model** or the **BSA** method above, or

2) Infusion of SIR-Spheres to the point of sluggish antegrade hepatic arterial flow, at which point further infusion of SIR-Spheres could result in non-targeted embolisation. The stopping point for the infusion of SIR-Spheres is at the discretion of the treating Interventional Radiologist.

The technique for delivery of SIR-Spheres is provided in the Sirtex Medical Training Manual and in Appendix 3.

Due to the relative increased risk of non-targeted delivery when a base catheter and proper hepatic arterial infusion is used to administer SIR-Spheres, the treating Interventional Radiologist is encouraged to administer SIR-Spheres using a microcatheter and superselective technique.

In instances where multiple vascular origins arising from the proper hepatic arteries are recognised, optimisation of flow and superselective administration of SIR-Spheres may include the following techniques:

1) Individual superselection with separate infusion of SIR-Spheres into each involved segment.

2) Optimisation of segments through selected branch vessel embolisation at the time of diagnostic hepatic angiogram, allowing for cross-perfusional filling.

   (Note: this technique cannot be performed if embolisation may result in complete exclusion of an entire vascular territory secondary to the crossing of the falciform ligament e.g. left hepatic artery cannot be embolised to optimise therapy from right hepatic artery, however middle hepatic artery may be embolised if anticipated right/left two point administration).

3) Free float technique: selection and partial embolisation with resultant free manipulation of a 'hot' microcatheter into a secondary vascular territory without removal of the catheter from the hepatic arterial bed.

At the discretion of the treating Interventional Radiologist, selective embolisation of the cystic artery may be performed.

11.1.3.1 Measurement of Residual SIR-Spheres Activity Post-Treatment

Once the pre-determined end-point for the administration of SIR-Spheres into the hepatic arterial circulation has been reached, the microcatheter will be removed from the patient and the amount of activity remaining in the SIR-Spheres v-vial, delivery tubing and microcatheter should be assayed, in order to determine the amount of activity that was actually administered to the patient. This is done by subtracting the residual SIR-Spheres activity from the pre-treatment SIR-Spheres activity, to arrive at an "administered activity". The method for measuring the residual activity of SIR-Spheres is at the discretion of the treating site.

Most centers measure residual SIR-Spheres activity using equidistant measurements with a G-M probe taken at four positions around the v-vial at 0°, 90°, 180°, 270° prior to, and immediately after treatment. Some centers place the v-vial, delivery tubing and microcatheter back into the dose calibrator which was used to assay the amount of SIR-Spheres activity during dose preparation. Either method is acceptable in this study protocol.

11.2 Protocol Treatment: Sorafenib

Sorafenib therapy will commence 14 days after (for the first patient cohort) or 11 days after (for the second patient cohort) the delivery of SIR-Spheres:

Sorafenib 400mg orally, twice daily.

Continued until: evidence of treatment failure (lack of efficacy resulting in disease progression); or unacceptable toxicity occurs.

11.2.1 Precautions

Every attempt should be made to continue Sorafenib therapy for a minimum of three months, at which time a clinical decision will be made to continue or not continue protocol therapy, based on the results of clinical assessment, serial CT scans and serological tests.

11.2.2 Duration of Treatment

Protocol therapy should be administered indefinitely and only modified or discontinued for one of the following reasons:

- Objective evidence of tumour progression at any site as determined by CT/MRI scan and/or ultrasound and/or clinical examination.

- No further response as determined by 2 consecutive scans
- Patient request.
- Patient cure or complete response.
- Patient responds to treatment and eligible for Surgery/ RFA
- Unacceptable toxicity as determined by objective evidence, clinical judgement or patient request (See section 11.2.4).

All Sorafenib therapy will be recorded on the patient CRF.

11.2.3 Sorafenib Dose and Schedule Modifications

The following tables (Table A, Table B, Table C, Table D and Table E) illustrate dose and schedule modifications for Sorafenib. The justification for the dose or schedule modification should be noted in the patient record and recorded on the CRF.

Table A: Hematologic Criteria for Dose Delay and Dose Modification of Sorafenib

Grade	Dose Delay	Dose Modification
Grade 0-2	Treat on time	No change
Grade 3	Treat on time	Decrease one dose level to 200 mg BD every day[b]
Grade 4	Delay [a] until ≤ Grade 2	Decrease one dose level to 200 mg BD every 2 days[b]

[a] If no recovery after 30 days delay, treatment will be discontinued unless patient is deriving clinical benefit
[b] If more than 2 dose reductions are required, treatment will be discontinued

Table B: Non-Hematologic Criteria for Dose Delay and Dose Modification of Sorafenib (except skin toxicity) [a]

Grade	Dose Delay	Dose Modification
Grade 0-2	Treat on time	No change
Grade 3	Delay [b] until ≤ Grade 2	Decrease one dose level to 200 mg BD every day [c]
Grade 4	Off protocol therapy	Off protocol therapy

[a] Also excludes nausea/vomiting that has not been premedicated, and diarrhea
[b] If no recovery after 30 days delay, treatment will be discontinued unless patient is deriving clinical benefit
[c] If more than 2 dose reductions are required, treatment will be discontinued

Table C: Management of Treatment-emergent Hypertension

Grade of Event (CTCAE v.3)	Management/Next Dose
Grade 1	Consider increased BP monitoring
Grade 2 asymptomatic and diastolic BP < 110 mmHg	Begin anti-hypertensive therapy and continue sorafenib
Grade 2 symptomatic/persistent<br>OR<br>diastolic BP ≥ 110 mmHg<br>OR	1. Sorafenib should be held [a] until symptoms resolve and diastolic BP ≤ 110 mmHg; also treat patient with anti-hypertensives and when sorafenib is restarted, reduce by 1 dose level to 200 mg BD every day.[a]
Grade 3	2. If diastolic BP not controlled (≤ 100) on therapy, reduce another dose level to 200 mg BD every 2 days [b]
Grade 4	Discontinue protocol therapy

[a] May be able to resume full dose later
[b] Patients requiring > 2 dose reductions should go off protocol therapy

11.2.3.1 Dose Modification of Sorafenib for Skin Toxicity

Patients experiencing Hand-Foot syndrome should have their signs and symptoms graded according to the following system (see Table D). Other skin toxicities will be graded according to CTCAE Version 3.0.

Table D: Grading for Hand-Foot Syndrome

Grade 1	Numbness, dysesthesia/paresthesia, tingling, painless swelling or erythema of the hands and/or feet and/or discomfort, which does not disrupt normal activities.
Grade 2	Painful erythema and swelling of the hands and/or feet and/or discomfort affecting the patient's activities.
Grade 3	Moist desquamation, ulceration, blistering or severe pain of the hands and/or feet and/or severe discomfort that causes the patient to be unable to work or perform activities of daily living.

According to the grade and incidence of skin toxicity (including rash and hand-foot syndrome) for a given patient, the following dose modification schedule will be followed (see Table E).

Table E: Skin Toxicity Criteria for Dose Delay and Dose Modification of Sorafenib

Toxicity Grade		During a Course of Therapy	Dose for Next Cycle
Grade 1		Maintain dose level	Maintain dose level
Grade 2	1st appearance	Interrupt until resolved to grade 0-1	Maintain dose level
	2nd appearance	Interrupt until resolved to grade 0-1	200 mg BD every day
	3rd appearance	Interrupt until resolved to grade 0-1	200 mg BD every 2 days
	4th appearance	Discontinue treatment permanently	
Grade 3	1st appearance	Interrupt until resolved to grade 0-1	200 mg BD every day[a]
	2nd appearance	Interrupt until resolved to grade 0-1	200 mg BD every 2 days
	3rd appearance	Discontinue treatment permanently	

[a] For patients who require a dose reduction for grade 3 rash or hand-foot syndrome, the dose of study drug may be increased to the starting dose after one full cycle of therapy has been administered at the reduced dose without the appearance of rash or hand for syndrome ≥ grade 1.

Patients who develop rash/desquamation or hand-foot skin reaction during treatment with sorafenib should have the involved area photographed if possible.

Patients with discomfort due to hand-foot syndrome may be treated with topical emollients, low potency topical steroids, or urea-containing cream.

For patients who require a dose reduction for grade 3 rash or hand-foot syndrome, the dose of study drug may be increased to the starting dose after one full cycle of therapy has been administered with the reduce dose, without the appearance of rash or hand-foot syndrome ≥ grade 1.

All other grade 3 toxicities related to study drug result in a permanent dose reduction.

11.2.4 Cessation of Protocol Treatment

Protocol treatment will be ceased if any of the following events occur:

- Life threatening grade 4 toxicity.
- Repetition of non-life threatening grade 4 toxicity in spite of adequate dose reduction.
- Anaphylactic reaction.
- Cardiac event ≥ grade 2.

- If Sorafenib therapy has to be delayed due to adverse events for more than 3 consecutive weeks

11.2.5 Non-Protocol Chemotherapy

Once protocol therapy has been discontinued, the patient should receive the best available care as determined by the treating Investigator. Patients are not permitted to receive second line therapy until they have documented progression of disease. The treating Investigator may continue to administer the same therapy regimen as was administered during protocol treatment and there are no restrictions on further treatment.

11.2.6 Supportive (Non-Protocol) Treatment

Supportive treatment should be given when required, according to the patient's condition. Such supporting treatment may include, but is not limited to, anti-emetics, pain relief etc. All supportive treatment should be recorded on the CRF, including any supportive treatment provided for the implantation of SIR-Spheres.

Prophylactic anti-emetic therapy (e.g. Ondansetron, Granisetron) may be administered at the discretion of the treating Investigator or as per usual institutional policy.

Prophylactic proton pump inhibitor therapy (e.g. Omeprazole or Pantoprazole) commencing 1 week prior to treatment with SIRT and continuing for 4 weeks post treatment is recommended, unless otherwise contra-indicated.

Prophylaxis of post-embolisation syndrome using a tapering dose of oral corticosteroids (e.g. Methyl-prednisolone or Dexamethasone) commencing on the first day post-SIRT is recommended, unless otherwise contra-indicated.

Oral analgesia (e.g. Ketorolac) may be required for 1 week following SIRT treatment to relieve pain from radiation injury and the embolic effect of SIR-Spheres, and liver capsular pain from tumour edema.

11.3 Concomitant Medications

All medications taken by patients, including medications that are unrelated to their cancer management should be recorded in the CRF. These include long-term as well as short-term or acute medications ongoing at the time of signature of the informed consent document or started any time after signature of the informed consent document until 28 days after the last dose of protocol therapy is administered.

Routine medications should be listed in the appropriate section and need only be recorded on the CRF at the commencement of protocol therapy, unless they are changed. Additional routine medications should be recorded on the CRF when they commence. Commencement and cessation dates for concomitant medications are required.

12 INVESTIGATIONS

12.1 Eligibility Assessment

Most of the screening tests required to confirm a patient's eligibility to receive protocol treatment on this study are performed routinely as part of standard care in the management of this disease. These results are acceptable for baseline measurements if they were taken within the 28 days screening period. All patients will be assessed to determine their eligibility and to document baseline tumour involvement of the liver. Screening tests include:

- Contrast enhanced triple phase CT scan of the liver, and chest, abdomen, pelvis
- Liver function tests (AST, ALT, ALP, bilirubin, albumin)
- Complete blood count (FBC)
- Electrolytes, urea, creatinine
- Serum AFP
- Pregnancy test if the patient is female and of reproductive age
- Consenting patients will undergo hepatic angiogram to determine the arterial blood supply to the liver and a break-through nuclear scan in order to assess the lung shunting (see Section 12.2 and Appendices 5 and 6).

12.2 Serial Study Measurements

All patients will be periodically assessed with the following minimum serial study measurements. Additional non-study assessments may occur at the discretion of the treating Investigator.

Physical examination	Baseline, 2 weeks after SIR-Spheres Treatment, 4 weekly thereafter and End of Study
CBC and platelets	Baseline, 2 weeks after SIR-Spheres Treatment, 4 weekly thereafter and End of Study
Liver function tests	Baseline, 2 weeks after SIR-Spheres Treatment, 4 weekly thereafter and End of Study
AFP	Baseline, 2 weeks after SIR-Spheres Treatment, 4 weekly thereafter and End of Study
CT scan of liver	12 weekly. If a complete or partial response is detected on CT scan, then an additional confirmatory CT scan will be performed as soon after 28 days as practicable and not more than 35 days in order to confirm the response.
Assessment for resection	Responding patients will be assessed by the internal review process for eligibility for surgical resection every 3 months until progression. Patients eligible for resection will be asked for permission to use any resected sample for research purposes.
Quality of life assessment	12 weekly until progression

The acceptable tolerances in the above time frames are:

12 weekly assessments may be +/- 2 weeks
4 weekly assessments may be +/- 1 week
2 weekly assessments may be +/- 2 days

Table 12.2 Study Treatment Plan

	Screening	During protocol therapy			End of Study	Post commencement of SIR-Spheres follow-up
		SIR-Sphers Treatment		Sorafenib Treatment		
Schedule	≤ 28 days prior to commencing protocol therapy	Day 1	Day 14	Every 4th week	As appropriate	12 weekly
Informed consent	✓					
Demographics	✓					
Medical history	✓					
Concurrent illness	✓					
Concurrent medications	✓	✓[j]	✓[j]	✓[j]	✓[j]	
Clinical assessment & physical exam	✓	✓[a]		✓[a]	✓	
Performance status	✓	✓[a]		✓[a]	✓	
Haematology	✓	✓[a]		✓[a]	✓	
Biochemistry	✓	✓[a]		✓[a]	✓	
Pregnancy test, if appropriate	✓[b]					
Serum AFP	✓	✓[i]	✓[i]	✓[i]	✓[i]	
CT chest / abdomen/ pelvis	✓			Every 12 weeks[e]		✓[f]
Assessment for resection	✓			Every 12 weeks [k]		
Hepatic angiogram	✓ [h]					
Tc-99MAA lung shunt study	✓ [h]					
Adverse events	From consent until 28 days post last dose of Sorafenib[c]					
SIRT		✓				
Sorafenib[d]			✓			
EQ-5D QoL	✓[g]			✓[g]	✓[g]	
Survival						✓

Notes to Study Plan:

(a) Tests must be performed on Day 1 before SIR-Spheres Treatment, 2 weeks after SIR-Spheres Treatment, Every 4th week during protocol therapy of Sorafenib Treatment and End of Study'

(b) Women of reproductive potential must have a negative urine or serum pregnancy test before commencing protocol treatment. This test should be repeated if pregnancy is suspected during the study.

(c) Follow any ongoing SIRT-related toxicity or SAE until resolution.

(d) Sorafenib 400mg given orally, twice daily commencing either 14 days post-SIRT or 11-days post-SIRT.

(e) Every 12 weeks from the beginning of Day 1. If a complete or partial response is detected, then perform a further CT scan 4 weeks later.

(f) 12 week post-commencement of SIR-Spheres follow-up scans only until disease progression has been confirmed in hepatic and extra-hepatic locations.

(g) EQ-5D Quality of life questionnaires filled out at baseline, 3, 6, 12, 24, 36 months then yearly thereafter plus at first progression of disease.

(h) Hepatic angiogram and Tc-99 MAA lung shunt study to be performed during 28 day screening period.

(i) Serum AFP to be performed on Day 1 before SIR-Spheres Treatment, 2 weeks after SIR-Spheres Treatment, Every 4th week during protocol therapy of Sorafenib Treatment and End of Study'

(j) New concurrent medication to be recorded Day 1 before SIR-Spheres Treatment, 2 weeks after SIR-Spheres Treatment, Every 4th week during protocol therapy of Sorafenib Treatment and End of Study up to 28 days post last dose of chemotherapy.

(k) Assessment for resection every 12 weeks until resection or disease progression.

13 FOLLOW-UP

Patients should remain on protocol treatment while it provides appropriate patient care. Data capture during this period is via the CRF.

Once patients have had progression of disease detected, data capture is via the follow-up CRF. During the follow-up period, the 12-weekly assessments will continue to be taken and recorded for 18 months after the end of study or until death, whichever occurs first.

For patients who had gone for Surgical Resection/ RFA (as a result of disease down-sizing) are to be followed for 5 years or until death, whichever occurs earlier.

14 CRITERIA FOR ASSESSING OUTCOME

The following criteria will be used to assess response to treatment and for the evaluation of study end points.

14.1 Toxicity and Safety (Primary Endpoint)

Toxicity will be assessed using the National Cancer Institute Common Terminology Criteria (NCI-CTC) version 3.0 (see Appendix 8). Patients are to be followed for safety during the study treatment period and until day 28 after the last cycle of chemotherapy.
Definitions and requirements for reporting adverse events (AEs) and serious adverse events (SAEs) are detailed in section 18.

14.2 Tumour Response Rate (Secondary Endpoint)

Response will be calculated using RECIST (Response Evaluation Criteria In Solid Tumours) criteria (Therasse, 2000).

14.2.1 RECIST Guidelines

All measurable lesions (lesions that can be accurately measured in at least one dimension with longest diameter > 20 mm with conventional techniques and > 10 mm with spiral CT scan) up to a maximum of five lesions per organ with a maximum of 10 lesions in total, representative of all involved organs, should be identified as target lesions and will be recorded and measured at baseline.

A sum of the longest diameter for all target lesions will be calculated and reported as a baseline sum longest diameter (LD). The baseline sum LD will be used as the reference with which to characterize the objective tumour response.

All other lesions (or sites of disease) should be identified as non-target lesions and should also be recorded at baseline. Measurements of these lesions are not required, but the presence or absence of each should be noted throughout follow-up.

14.2.2 Response Criteria

Complete Response (CR): Disappearance of all target lesions associated with the disappearance of all non-target lesions and normalization of the tumour marker. CR is confirmed if determined by two observations not less than 4 weeks apart.

Partial Response (PR): At least a 30% decrease in the sum of the longest diameter of the target lesions, taking as a reference the baseline sum longest diameter, or a CR associated with an abnormal tumour marker and/or persistence of non-target lesions.

Progressive Disease (PD): At least a 20% increase in the sum of the longest diameter of the target lesions, taking as a reference the smallest sum longest diameter recorded since treatment started or the appearance of new lesions.

Stable Disease (SD): Neither sufficient shrinkage to qualify for a partial response nor sufficient increase to qualify for progressive disease, taking as a reference the smallest sum longest diameter since the start of treatment.

Notes:

(1) Elevated serum AFP alone, is not sufficient evidence of progression and requires imaging examinations to assess disease progression.

(2) As patients may experience downsizing of their liver metastases and become candidates for surgical resection either during or after protocol treatment, it is possible that undiagnosed disease may be found at the time of attempted resection. For the purpose of defining Progressive Disease, any disease that is found solely as a result of the patient undergoing laparotomy for resection will not be considered as Progressive Disease.

14.3 Progression-Free Survival (Secondary Endpoint)

Progression-free survival (PFS) is defined as the time interval between study entry and the date of tumour progression or death, whichever is earlier. Tumour progression in the liver is determined from serial CT scans. Tumour progression at other sites is measured by any definitive imaging technique including CT scan, MRI study, or PET or other nuclear medicine scan.

Diagnosis of tumour recurrence (progression of disease) should be made using RECIST Criteria.

The documented date of recurrence will be the date of confirmation of the recurrence. At the time of recurrence, the Investigator should clearly indicate the site of tumour recurrence (hepatic or extra-hepatic).

14.4 Survival (Secondary Endpoint)

All patients will be followed until death.

14.5 Quality of Life (Secondary Endpoint)

Quality of life (QoL) will be measured by using the EQ-5D questionnaire. The EQ-5D (see Appendix 9) will be collected at baseline, prior to commencing protocol treatment and then at 3, 6, 12, 24 and 36 month intervals, then yearly thereafter. It will also be collected at the time of first disease progression.

The EQ-5D questionnaire will only be taken until first disease progression.

15 STATISTICAL CONSIDERATIONS & METHODOLOGY

15.1 Study Design and Sample Size

This Phase I/II study will evaluate the safety and initial effectiveness of combining Sorafenib therapy with SIR-Spheres (chemo-radiotherapy) for the treatment of patients with primary HCC in whom surgical resection is not feasible.

This study is designed as a prelude to a future randomised comparative study that will compare the efficacy of Sorafenib therapy with SIR-Spheres against Sorafenib therapy alone, in this patient population.

The primary aim of this study is to determine the safety profile of the combination of Sorafenib when combined with SIRT. The following grade 3 toxicities are known events that are commonly associated with Sorafenib and/or SIRT therapy and thus should not constitute due cause for this study to be stopped should they occur:

- Abdominal pain (associated with the implantation of SIR-Spheres).
- Nausea (associated with the implantation of SIR-Spheres).
- Neutropaenia (associated with Sorafenib).
- Leucopaenia (associated with Sorafenib).
- Diarrhoea (associated with Sorafenib).

The primary toxicities of interest in this study are those that are known to be associated with Sorafenib therapy, which could be expected to be more frequent or of greater severity when Sorafenib therapy is administered in combination with SIRT therapy. This includes all grade 3 toxicity aside from those listed above, and all grade 4 toxicities. The following safety monitoring scheme will be used:

<u>First patient cohort</u> of 3 to 6 patients: Sorafenib commencing <u>14 days post-SIRT</u>
For the first 3 patients enrolled into this cohort, if the number of grade 3/4 toxicities is:
> 1 then stop the study
= 1 then enrol 3 more patients. If the total number of toxicities for 6 patients is greater than or equal to 2 then stop the study. Otherwise proceed to the next cohort
= 0 then proceed to the next cohort

<u>Second patient cohort</u> of 3 to 6 patients: Sorafenib commencing <u>11 days post-SIRT</u>
For the first 3 patients enrolled into this cohort, if the number of grade 3/4 toxicities is:
> 1 then proceed to the Phase II part of the trial using Sorafenib commencing <u>14 days post-SIRT</u>
= 1 then enrol 3 more patients. If the total number of toxicities for 6 patients is greater than or equal to 2 proceed to the Phase II part of the trial using Sorafenib commencing <u>14 days post-SIRT</u>. Otherwise proceed to Phase II using Sorafenib commencing <u>11 days post-SIRT</u>
= 0 then proceed to Phase II using Sorafenib commencing <u>11 days post-SIRT</u>

For the Phase II part of the trial, we assume a target tumour response rate of 30% and a no further

interest response rate of 10%. Then using A'Hern's single stage design (A'Hern, 2001), with a type 1 error of 0.05 and power of 80%, a sample size of 25 patients will be required. These 25 will include the 3 to 6 already recruited under the appropriate phase I cohort. If at least 6 responses are observed (6/25), we would conclude potential efficacy.

16 DEVIATIONS AND AMENDMENTS

16.1 Deviations From the Study Protocol

The Investigator will record any deviation from the study protocol and provide an explanation for such a deviation. Deviations shall be reviewed to determine if the study protocol requires amendment or if the study requires termination.

Reasons for withdrawal and/or discontinuation of any patient from the study will be recorded. If the discontinuation is related to toxicity or lack of effectiveness, the patient will still be followed in the study, wherever possible.

Where relevant, the Ethics Committee and/or regulatory bodies will be notified of any deviations.

16.2 Amendments to the Study Protocol

All amendments to the study protocol will be approved by the Study Management Committee and modifications will be recorded with a justification for the amendments.

16.3 Early Stopping Due to Toxicity

Notwithstanding the potential benefit of treatment, if excess toxicity is observed, then this may require any of the following:

- Treatment modification
- Dose reduction
- Temporary suspension of therapy
- Discontinuation of therapy
- Stopping the study earlier than planned.

These toxicities will be reviewed by the IDSMC both at the formal interim analysis after the first 6 patients have been followed for 3 months, and continuously as grade 3 or 4 adverse events are reported.

17 ADVERSE EVENTS

17.1 Definitions

17.1.1 Adverse event (AE) (ISO 14155-1:2003)

any untoward medical occurrence experienced by a subject and which does not necessarily have a causal relationship with any component of the study treatment.

An Adverse Event can be any sign, abnormal laboratory value, symptom or diagnosis/disease that is unfavorable or unintended, that is new, or if pre-existing, worsens in a patient, and that may or may not be related to the investigational treatment.

Adverse Events will be recorded from the date of signature of the informed consent up to 28 days after the last dose of chemotherapy is administered. If the AE is a SIRT-related toxicity, follow-up will continue until resolution.

17.1.2 Serious adverse device effect (ISO 14155-1:2003)

An adverse device effect that has resulted in any of the consequences characteristic of a serious adverse event or that might have led to any of these consequences if suitable action had not been taken or intervention had not been made or if circumstances had been less opportune.

17.1.3 Serious adverse event (SAE) (ISO 14155-1:2003)

Adverse event that

a) led to a death,
b) led to a serious deterioration in the health of the subject that
    1) resulted in a life-threatening illness or injury,
    2) resulted in a permanent impairment of a body structure or a body function,
    3) required in-patient hospitalization or prolongation of existing hospitalization,
    4) resulted in medical or surgical intervention to prevent permanent impairment to body structure or a body function.
c) led to fetal distress, fetal death or a congenital abnormality or birth defect.

17.2 Reporting

In order to adhere to all applicable laws and regulations for evaluating and reporting adverse events, the Investigational site must formally notify relevant IRB as well as Bayer and Sirtex of any **Serious adverse device effect, Serious adverse event (SAE)** or **Unanticipated adverse device effects** within 24 hours of becoming aware of it.

If an SAE is unresolved when a patient permanently discontinues the study, the patient must be followed until the SAE resolves or the clinical course is stabilized.

17.3 Pregnancy During the Study

Protocol therapy must be discontinued immediately in the event of pregnancy in a female patient enrolled in this study.

18 ETHICAL CONSIDERATIONS

This study will be performed in accordance with ISO 14155-1 2003 which includes a requirement to operate in accordance with the World Medical Association Declaration of Helsinki (see Appendix 11). The Investigator must comply with all instructions, regulations and agreements in this clinical investigation plan, using applicable GCP guidelines. The Investigator must additionally ensure that the study is conducted in accordance with all applicable local regulations.

All participating institutions must obtain approval from their institution's Human Research Ethics Committee.

18.1 Informed Consent

Written informed consent is required prior to commencing protocol treatment. The consent form is filed in the patient record.

Patients will be given a full explanation, in lay terms, of the aims of the study and the potential benefits as well as the possible side effects and risks involved. It will be explained that they may refuse to take part in, or withdraw from the study without prejudice to their future care and treatment.

Written informed consent will be obtained from all patients prior to study entry. Consent to participate in this study will be obtained from the patient both verbally and in writing. In the case where the patient is not fluent in English an interpreter will be present during the consenting process. Patients will be issued with a copy of the information provided and their consent to participate in the study. All Informed Consent Documents used in this study must be approved by the relevant Human Research Ethics Committee.

A copy of the Informed Consent Document can be found in Appendix 13.

18.2 Confidentiality

All patient data collected as a part of this study will be treated according to ISO 14155-1:2003 Part 6.5 Confidentiality concerning the protection of patient information at all times. All data generated from this study will remain confidential and no published report will contain any reference to patient identifiable data.

18.3 Changes to Final Study Plan

All study amendments must be submitted to the relevant Ethics Committee. Study modifications that impact subject safety, the scope of the investigation, or affect the scientific quality of the study must be approved by the Ethics Committee. In the event of a study protocol modification, the Informed Consent Document may require similar modifications.

19 PUBLICATION POLICY

The results of this Phase I/II study will be submitted for publication upon completion by the investigators. In advance of any publication, we have agreed to notify the manufacturer of the report and provide them a copy. As an investigator-initiated study, the manufacturer (Sirtex Medical) does not have ownership of the original data or influence over what data can be submitted for publication and/or presentation at meetings.

20 ADMINISTRATIVE PROCEDURES

20.1 Investigator File

The Investigator will be provided with an Investigator File. This file should be used for filing all study-related documents. The Investigator will be responsible for keeping the Investigator File updated and for ensuring that all required documents are filed during and after the study. The Investigator File will be inspected during monitoring visits and will remain with the Investigator for 15 years after study closure.

20.2 Quality Assurance

During and/or after completion of the study, regulatory authorities may wish to perform on-site audits. The Investigator and site personnel will be expected to cooperate with any audit and to provide assistance and documentation (including source data) as requested.

20.3 Study Completion

The Ethics Committee must be notified of completion or termination of this study in a timely manner. The Investigator must provide a final clinical summary report to the Ethics Committee and maintain in the Investigator File an accurate and complete record of all submissions made to the Ethics Committee.

21 BIBLIOGRAPHY

The following references were used during the preparation of this document.

Adam R, Avisar E, Ariche A et al. Five-year survival following hepatic resection after neoadjuvant therapy for nonresectable colorectal liver metastases. Ann Surg Oncol 2001; 8(4): 347-353.

Advanced Colorectal Cancer Meta-analysis Project. Modulation of Fluorouracil in patients with advanced colorectal cancer: evidence in terms of response rate. J Clin Oncol 1992; 10: 896-903.

Aschele C, Friso ML, Pucciarelli S et al. A phase I-II study of weekly oxaliplatin, 5-fluorouracil continuous infusion and preoperative radiotherapy in locally advanced rectal cancer. Ann Oncol 2005; 16: 1140-1146.

Bismuth H, Adam R. Reduction of nonresectable liver metastases from colorectal cancer after oxaliplatin chemotherapy. Semin Oncol 1998; 25(2): 40-46.

Blackstock A, Tepper J, Hess S. Oxaliplatin: in vitro and in vivo evidence of its radiation sensitizing activity. Int J Rad Oncol Biol Phys 2000; 46: 92-94.

Blackstock A, Tepper J, Case L et al. In vitro and in vivo radiosensitization of colon cancer cells by Oxaliplatin. Int J Mol Med, 2002, 10: S68, Abstr 533.

Boyle P, Langman JS. ABC of colorectal cancer: Epidemiol. BMJ 2000; 321: 805-808.

Campbell A, Bailey I, Burton M et al. Analysis of the distribution of intra-arterial microspheres in human liver following hepatic yttrium-90 microsphere therapy. Physics in Medicine and Biology 2000; 45: 1023-1033.

Carrato A, Gallego J, Diaz-Rubio E. Oxaliplatin: results in colorectal carcinoma. Crit Rev Oncol Hematol 2002; 44(1): 29-44.

Chan R, Au G, Ho J, et al. Radiation recall with oxaliplatin: report of a case and a review of the literature. Clin Oncol (R Coll Radiol). 2001; 13:55-57.

Chau I, Cunningham D, et al. Adjuvant therapy in colon cancer - what, when and how? Ann Oncol 2006; 17:1347-1359.

Chollet P, Bensmaine MA, Brienza S et al. Single agent activity of oxaliplatin in heavily pretreated advanced epithelial ovarian cancer. Ann Oncol 1996; 7(10):1065-1070.
Cohen M, Gootenberg J, Keegan P, et al. FDA Drug approval summary: Sorafenib plus FOLFOX4 as second-line treatment of colorectal cancer. Oncologist 2007; 12:356-361.

Freyer G, Bossard N, Romestaing P et al. Addition of oxaliplatin to continuous fluorouracil, l-folinic acid, and concomitant radiotherapy in rectal cancer: the Lyon R 97-03 phase I trial. J Clin Oncol 2001;

19(9):2433-2438.

Gilbert HA, Kagan AR. Metastases: Incidence, detection and evaluation without histologic confirmation. In Weiss L (ed): Fundamental aspects of metastases. Elsevier, Amsterdam, The Netherlands. 1976:385-405.

Goldberg R, Sargent D, Morton R et al. A randomised controlled Study of Fluorouracil plus Leucovorin, Irinotecan, and Oxaliplatin combinations in patients with previously untreated metastatic colorectal cancer. J Clin Oncol 2004; 22: 23-30.

Gray B, Anderson J, Burton M et al. Regression of liver metastases following treatment with tumour microspheres. Aust NZ J Surgery. 1992; 62: 105-110.

Gray B, Van Hazel G, Hope M, et al. Randomised trial of SIR-Spheres plus chemotherapy vs. chemotherapy alone for treating patients with liver metastases from primary large bowel cancer. Annals of Oncology 2001; 12: 1711-1720.

Hess C, Vuong V, Hegyi I, et al. Effect of VEGF receptor inhibitor PTK787/ZK222548 combined with ionizing radiation on endothelial cells and tumour growth. Br J Cancer 2001; 85: 2010-2016.

Hirakawa M, Iida M, Aoyagi K et al. Gastroduodenal lesions after transcatheter arterial chemo-embolisation in patients with hepatocellular carcinoma. Am J Gastroenterol. 1988; 83(8): 837-840.

Hurwitz H, Fehrenbacher L, Novotny W, et al. Sorafenib plus Irinotecan, Fluorouracil, and Leucovorin for Metastatic Colorectal Cancer. New England Journal of Medicine 2004; 350: 2335-2342.

Kemeny N, Daly J, Oderman P et al. Hepatic artery pump infusion: toxicity and results in patients with metastatic colorectal carcinoma. J Clin Oncol. 1984; 2(6): 595-600.

Kennedy A, Coldwell D, Nutting C et al. Resin 90Y-microsphere brachytherapy for unresectable colorectal liver metastases: Modern USA experience. Int J Radiation Oncology Biol Phys. 2006; 65: 412-425.

Kennedy A, Nutting C, Coldwell D, et al. Pathologic response and microdosimetry of 90Y microspheres in man: review of four explanted whole livers. Int J Radiation Oncology Biol Phys 2004; 60:1552-1563.

Kjellstrom J, Kjellen E, Johnsson A. In vitro radiosensitization by oxaliplatin and 5-fluorouracil in a human colon cancer cell line. Acta Oncol. 2005; 44:687-693.

Leung T, Lau W, Ho S et al. Radiation pneumonitis after selective internal radiation treatment with intra-arterial tumour-microspheres for inoperable hepatic tumours. Int J Radiation Oncology Biol Phys 1995; 33;919-924.

Liu D, Salem R, Bui J et al. Angiographic considerations in patients undergoing liver-directed therapy. J Vasc Inter Radiol. 2005; 16:911-935.

Louvet C, de Gramont A. Colorectal Cancer: Integrating Oxaliplatin. Current Treatment options in Oncology 2003; 4:405 - 411.

Machover D. A comprehensive review of 5-fluorouracil and leucovorin in patients with metastatic colorectal carcinoma. Cancer 1997; 80:1179-1187.

Machover D, Diaz-Rubio E, de Gramont et al. Two consecutive phase II studies of oxaliplatin for treatment of patients with advanced colorectal carcinoma who were resistant to previous treatment with fluoropyrimidines. Ann Oncol, 1996; 7:95-98.

Meta-analysis group in cancer. Reappraisal of hepatic arterial infusion in the treatment of non-resectable liver metastases from colorectal cancer. J. Natl. Cancer Inst. 1996; 88: 252-282.

Meade V, Burton M, Gray B et al. Distribution of different sized microspheres in experimental hepatic tumours. Eur J Clin Oncol. 1987; 23:37-41.

Metha C and Cain K. Charts for the Early Stopping of Pilot Studies. Am J Clin Oncol, 1984; 6:676-682,

Poon MA, O' Connell MJ, Moertel C et al. Biochemical modulation of Fluorouracil; evidence of significant improvement of survival and quality of life in patients with advanced colorectal carcinoma. J Clin Oncol 1989; 7:1407-18).

Raymond E, Buquet-Fagot C, Djelloul S et al. Antitumour activity of oxaliplatin in combination with 5-fluorouracil and the thymidylate synthase inhibitor AG337 in human colon, breast and ovarian cancers. Anticancer Drugs 1997; 8:876-885.

Ryan D. Rectal cancer: integrating oxaliplatin into chemoradiation studies. Oncology (Huntingt). 2000; 14:38–41.

Scheele J, Stangl R, Altendorf-Hofmann A. Hepatic metastases from colorectal carcinoma: Impact of surgical resection on the natural history. Br J Surg 1990; 77:1241-1246.

Sharma R, van Hazel G, Blanshard K et al. Internal Radiation Treatment of Liver Metastases From Colorectal Cancer With Concomitant Systemic Radiosensitizing Chemotherapy, WCGI. Barcelona, 2006.

Silverberg E. Cancer Statistics 1977. CA Cancer J Clin. 1977; 27(1):26-41.

Stangl R, Altendorf-Hofmann A, Charnley RM et al. Factors influencing the natural history of colorectal liver metastases. Lancet, 1994; 343:1405-1410.

Stubbs R, O'Brien I, Correia M. Selective Internal Radiation Therapy with 90Y Microspheres for colorectal liver metastases: single-centre experience with 100 patients. ANZ J Surg. 2006; 76:696 – 703.

Stubbs R, Wickremesekera S. Selective internal radiation therapy (SIRT): a new modality for treating

patients with colorectal liver metastases. HPB 2004; 6:133 - 139.

Therasse P, Arbuck S, Eisenhauer E et al. New guidelines to evaluate the response to treatment in solid tumours. Journal of the National Cancer Institute, 2000; 92:205-216.

Turitto G, Panelli G, Frattolillo A et al. Phase II study of neoadjuvant concurrent chemoradiotherapy with oxaliplatin-containing regimen in locally advanced rectal cancer. Front Biosci. 2006; 11:1275-1279.

Van Hazel G, Blackwell A, Anderson J, et al. Randomised phase 2 trial of SIR-Spheres plus Fluorouracil/Leucovorin chemotherapy versus Fluorouracil/Leucovorin chemotherapy alone in advanced colorectal cancer. J Surg Oncol. 2004; 88:78-85.

Van Hazel G, Blanshard K, Steward W, et al. Selective Internal Radiation Therapy (SIRT) Plus Systemic Chemotherapy with Oxaliplatin, 5-Fluorouracil and Leucovorin: A Phase I Dose Escalation Study. ASCO GI Symposium, 2005.

Van Hazel G, Pavlakis N, Goldstein D, et al. Selective Internal Radiation Therapy (SIRT) plus systemic chemotherapy with Irinotecan. A phase I dose escalation study. ASCO GI Symposium 2005.

Van Hazel G, Pavlakis N, Goldstein D, et al. SIR-Spheres plus systemic chemotherapy with irinotecan-based treatment arms. Interim report on a phase I dose escalation study. Clinical Oncology Society of Australia. Canberra; 2004.

Vietti J, Eggerding F, Valeriote E. Combined effect of radiation and 5FU on survival of transplanted leukemia cells. J Nat Cancer Inst. 1971, 47:865-870.

Walsh L, Poston G. The potential to increase curative liver resection rates in metastatic colorectal cancer. Eur J Surg Oncol 2002; 28(8): 812-814.

Welch J, Donaldson G. The clinical correlation of an autopsy study of recurrent colorectal cancer. Annals of Surgery. 1979; 189:496 - 502.

WHO handbook for reporting results in cancer treatment. Geneva (Switzerland): World Health Organisation Offset Publication No.48; 1979.

Wong C, Savin M, Sherpa K, et al. Regional Tumour Microsphere Treatment of Surgically Unresectable and chemotherapy-refractory Metastatic Liver Carcinoma. Cancer Biotherapy & Radiopharmaceuticals. 2006; 21:305-313.

Woynarowski J et al. Mechanisms of action of Oxaliplatin: oxaliplatin-induced lesions in cellular DNA; San Antonio (TX): Institute for Drug Development Report; 1997 Jan. Research Report.

APPENDIX 1 ECOG Performance Status (Definitions)

ECOG Performance Status	Patient Description
0	Fully active, able to carry on all pre-disease performance without restriction.
1	Restricted in physically strenuous activity but ambulatory and able to carry out work of a light or sedentary nature, e.g., light house work, office work.
2	Ambulatory and capable of all self-care but unable to carry out any work activities. Up and about more than 50% of waking hours.
3	Capable of only limited self-care, confined to bed or chair more than 50% of waking hours.
4	Completely disabled. Cannot carry on any self-care. Totally confined to bed or chair.
5	Dead.

APPENDIX 2 Consumer Medicine Information For Sorafenib

CONSUMER MEDICINE INFORMATION

Generic name: Sorafenib

Brand name: Nexavar

This is a summary of the most important information about Nexavar. For details, talk to your healthcare professional.

What Is Nexavar?

Nexavar is an anticancer medicine to treat adults with kidney cancer called advanced renal cell carcinoma or with liver cancer called hepatocellular carcinoma.

Nexavar has not been studied in children.

Who Should Not Take Nexavar?

You should not take Nexavar if you are allergic to anything in it.

What Are The Risks?

The following are the major potential risks and side effects of Nexavar therapy. However, this list is not complete.

The following are the major potential risks and side effects of Nexavar therapy:

- **Birth defects or death of an unborn baby. Nexavar may cause birth defects or death of an unborn baby.** Women should not get pregnant during treatment with Nexavar and for at least 2 weeks after stopping treatment. Men and women should use effective birth control during treatment with Nexavar and for at least 2 weeks after stopping treatment. Call your doctor right away if you become pregnant during treatment with Nexavar.
- **A skin problem called hand-foot skin reaction.** This causes redness, pain, swelling, or blisters on the palms of your hands or soles of your feet. If you get this side effect, your doctor may adjust your dose or stop treatment for some time.
- **High blood pressure.** Your blood pressure should be checked weekly during the first 6 weeks of starting Nexavar. High blood pressure should be monitored and treated during treatment with Nexavar.
- **Heart problems.** Talk to your doctor about these potential problems.
- **Bleeding problems.** Nexavar may increase your chance of bleeding.
- **Some common side effects** that may occur with Nexavar include:
  - rash, redness or itching of your skin

- hair thinning or patchy hair loss
- diarrhea (frequent and/or loose bowel movements)
- nausea and/or vomiting
- mouth sores
- weakness
- loss of appetite
- numbness, tingling or pain in your hands and feet

What Should I Tell My Healthcare Professional?

Before you start taking Nexavar, tell your healthcare professional if you:

- Have kidney problems in addition to kidney cancer
- have liver problems
- have high blood pressure
- have bleeding problems
- have heart problems or chest pain
- are trying to become pregnant, are already pregnant, or are breast-feeding

Can Other Medicines Or Food Affect Nexavar?

Nexavar and certain other medicines can interact with each other. Tell your healthcare professional about all the medicines you take including prescription and non-prescription medicines, vitamins, and herbal supplements. Know the medicines you take. Keep a list of them with you to show your healthcare professional.

Especially tell your healthcare professional if you take:

- Warfarin (Coumadin)

How Should I Take Nexavar?

- Take Nexavar exactly as prescribed. You will stay on Nexavar as long as your doctor thinks it is helping you.
- Swallow Nexavar tablets whole with water.
- Take Nexavar on an empty stomach (at least 1 hour before or 2 hours after a meal).

Date Created: February 2, 2006

APPENDIX 3 Technique for Administration of SIR-Spheres

SIR-Spheres are administered by injection through a trans-femoral catheter into the hepatic artery. As there are frequent arterial anomalies in the blood supply to the liver, the radiologist must be familiar with those anomalies. Only radiologists that have received formal training and have been approved by Sirtex may participate in this study and administer SIRT. All areas of tumour within the liver are to be targeted with SIR-Spheres and this usually involves treating both lobes of the liver. However, it is essential that the SIR-Spheres are not delivered to other organs such as the duodenum, stomach, pancreas etc.

If the metastases are limited to only one lobe, the radiologist can insert the catheter selectively into the lobar artery supplying only that lobe that contains the metastases. The SIR-Spheres will then be delivered only to the lobe containing the metastases with sparing of the other normal lobe. This is an excellent way of delivering high doses of radiation to the tumour without any chance of damaging the normal liver.

It is most important to inject the SIR-Spheres slowly into the hepatic artery. If they are injected too quickly they may reflux back down the hepatic artery and lodge in the pancreas, stomach and other organs.

The following principles dictate where the catheter is placed when delivering SIRT:

- Treat only those parts of the liver that contain tumour.
- If possible then try and not treat some normal liver parenchyma from SIRT. Even a small amount of normal liver that remains untreated provides extra protection against the possibility of radiation hepatitis.
- Review with extreme vigilance the pre-treatment angiogram to look for anatomical abnormalities.
- Never allow SIR-Spheres to enter any vessel supplying the gut.
- Always inject SIR-Spheres slowly with repeated fluoroscopy to check the position of the catheter and to look for slowing of blood flow and possible reflux of SIR-Spheres.

Dealing with Anatomical Anomalies

Note: Inadvertent injection of SIR-Spheres into small arteries passing from major arteries vessels in the hilum of the liver to the stomach and duodenum is by far the commonest cause of serious adverse events. These small aberrant vessels are NOT described in the standard anatomy texts and the cause of these SAEs is because the radiologists do not recognise these small vessels and allow SIR-Spheres to flow to the gut.

Radiologists are referred to the review article of Liu et al, 2005 (below) attached to this protocol for an additional comprehensive description of how to administer SIRT.

If there is a dual blood supply to the liver, then the radiologist will have to catheterise each artery

separately to inject the SIR-Spheres if there is tumour in both lobes. If there is only tumour in one lobe, then the radiologist only needs to inject the SIR-Spheres into that side of the liver.

For instance, if all the metastases were in the right lobe of the liver and there was an accessory right hepatic artery arising from the superior mesenteric artery, then injecting all the SIR-Spheres into this accessory right hepatic artery would deliver all the radiation to metastases in the right lobe where it is wanted.

If there are separate right and left arteries and there are metastases in both right and left lobes, then it is necessary to inject some of the SIR-Spheres separately into both arteries in order to deliver radiation to the metastases in both lobes.

The following anomalies in vascular supply must be noted:

1. In 20% of patients there will be an accessory right hepatic artery arising from the superior mesenteric artery (see diagram b below). This accessory right hepatic artery will supply most of the right lobe of liver and is easily demonstrated on an angiogram. If present, it must be accessed to deliver SIR-Spheres to the right lobe of the liver as well as the main hepatic artery, otherwise the radiation will not be delivered to metastases in the right lobe of the liver.
2. In 17% of patients an accessory left hepatic artery will arise from the left gastric artery (see diagram c below). This accessory left artery is usually difficult to demonstrate on an angiogram, and is often not recognised at the time of angiography. It is usually possible to get a co-axial catheter into this artery if it is necessary to deliver SIR-Spheres to the left lobe of the liver. If there is no tumour in the left lobe then it can be ignored.
3. In a minority of patients the gastro-duodenal artery arises from the main hepatic artery distal to the origin of the left hepatic artery. It is imperative that the SIR-Spheres not be delivered into the gastro-duodenal artery, as this will result in the SIR-Spheres lodging in the duodenum and pancreas with severe side effects. In this situation the gastro-duodenal artery should be embolised to occlude it before administering the SIR-Spheres into the hepatic artery.

MAJOR VARIATIONS IN ARTERIAL BLOOD SUPPLY TO THE LIVER

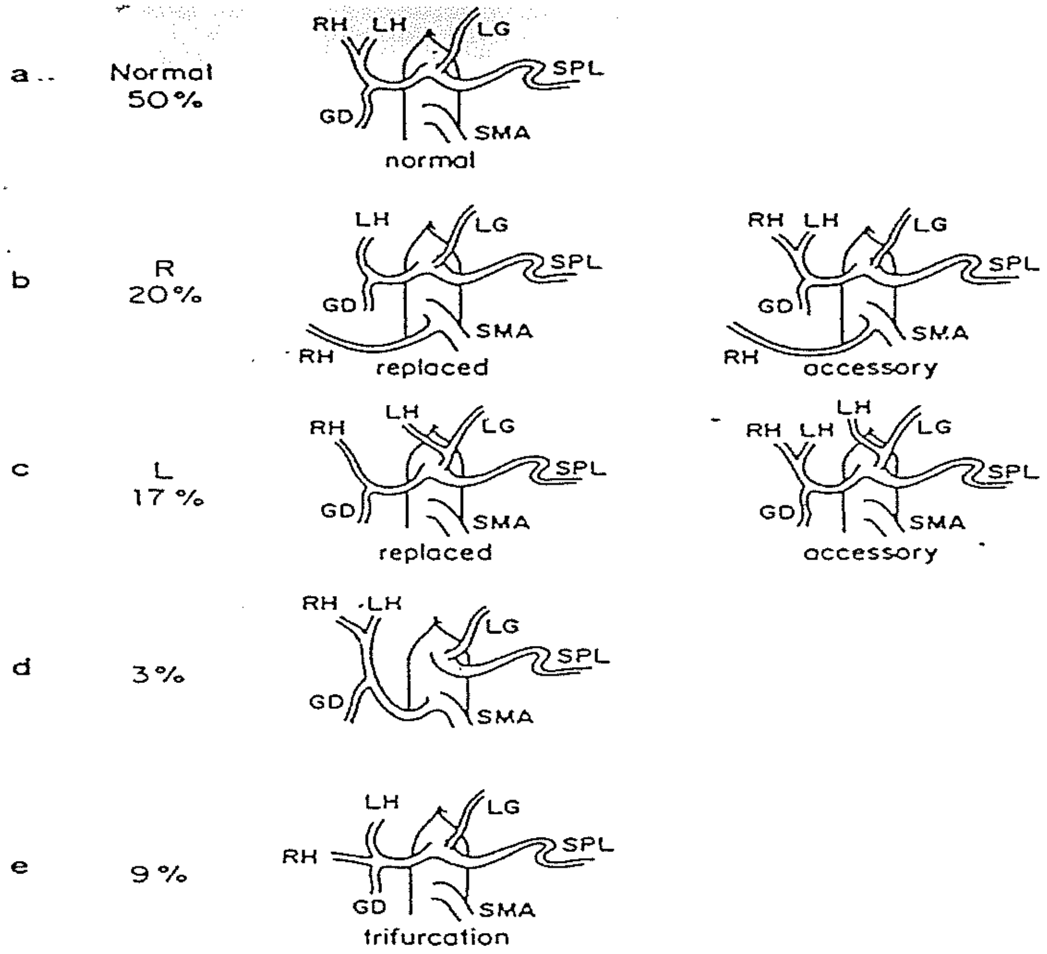

a (50%) In the normal setting the gastro-duodenal (GD) artery comes off the common hepatic artery proximal to the bifurcation into the right hepatic (RH) and left hepatic (LH) arteries. The left gastric (LG) and splenic (SPL) arteries come off the celiac axis separately.
b: (20%) When the right hepatic artery is replaced the whole blood supply to the right lobe comes off the superior mesenteric artery (SMA). In the case of an accessory right hepatic artery, the vasculature off the celiac axis is normal but there is an additional right hepatic artery off the superior mesenteric artery.
c: (17%)When the left hepatic (LH) artery is replaced, the whole blood supply to the left lobe comes off the left gastric (LG) artery. In the case of an accessory left hepatic artery the vasculature of the common hepatic artery is normal but there is an additional left hepatic artery off the left gastric artery.
d: (3%) In this situation the entire common hepatic artery arises from the superior mesenteric artery
e: (9%) A trifurcation occurs when the bifurcation of the left hepatic and right hepatic arteries occurs at the same spot as the take off of the gastro-duodenal (GD) artery.

APPENDIX 4 Nuclear Medicine Tc-99 MAA Lung Shunt Study

**Purpose:**	To assess arterial perfusion of the liver and the fraction of radiopharmaceutical tracer that will pass through the liver and lodge in the lungs.
**Agent:**	Technetium-99 labelled MAA (Macro-aggregated Albumin)
**Dose:**	150MBq
**Equipment:**	Any large FOV gamma camera
**Administration:**	The patient needs to have a trans-femoral catheter placed in the hepatic artery. The Technetium 99 labelled MAA is injected through the catheter into the hepatic artery by a qualified physician.
**Imaging:**	The patient is positioned supine under the gamma camera and the images recorded.
**Analogue:**	Anterior and posterior images of abdomen and thorax. Measure 700 - 1000 counts for abdomen and equivalent time for thorax.<br>Right lateral abdomen - same time acquisition as for Anterior.
**Digital:**	4 frames; 300"/ frame. 64 x 64 matrix Word mode. Image anterior and posterior abdomen<br>Image anterior and posterior thorax
**Analysis:**	Draw ROI around whole of liver and whole of lung fields. Calculate G mean for liver region and lung region.<br>Calculate Lung/liver ratio
**Interpretation:**	If lung/liver ratio is >10% then there is need for dose reduction of SIR-Spheres.

APPENDIX 5 SIR-Spheres Radiation Absorbed Dose/Activity Calculation Procedure

The step-by-step procedures required for calculation of the various dosimetric parameters using the partition model are as follows (see section 11.1.1 above for details on the derivation of the various partition model formulas):

1. Perform a contrast-enhanced CT scan of the liver no more than one month before scheduled date of treatment with SIR-Spheres.

2. From the CT scan of the abdomen, obtain volume estimates of the entire liver, V_{total} (right and left hepatic lobes, including tumour) and tumour, V_{tumour}, and normal liver, $V_{liver} = V_{total} - V_{tumour}$. Calculate masses from volumes:

$$M_{total}, M_{tumour} \text{ or } M_{liver} = V_{total}, V_{tumour} \text{ or } V_{liver} \text{ in cc} \times 1.03 \text{ g/cc}$$

3. Following hepatic arterial infusion of 4 mCi ^{99m}Tc MAA, estimate percent lung shunting (L) from a nuclear medicine break-through scan, consisting of planar imaging of the liver and lung fields (in the same image) in anterior (A) and posterior (P) projections (128 × 128 matrix, 700 – 1000 k counts). Regions of interest (ROIs) are drawn around the total liver (including tumour) and both lungs on the A image, and mirrored onto the P image. Percent lung shunting, L, is calculated as 100 times the ratio of geometric mean total counts in the lung ROIs ($[N_{lungA} \times N_{lungP}]^{1/2}$) to that of liver plus lungs ($[N_{liverA} \times N_{liverP}]^{1/2} + [N_{lungA} \times N_{lungP}]^{1/2}$).

4. Immediately following the break-through scan, perform ^{99m}Tc MAA SPECT imaging of the liver (128 × 128 matrix, 120 views over 360°, 20 sec/view, with attenuation correction), to estimate the SIR-Spheres microspheres tumour-to-normal activity ratio (activity per unit mass of the organ or tissue, R). The value R represents the ratio of concentrations of SIR-Spheres microspheres in the tumour and normal liver compartments after delivery into the hepatic artery, corrected for any SIR-Spheres microspheres that are shunted to the lungs calculated from the nuclear medicine break-through scan. For up to five (5) of the largest tumours visible in the SPECT volume, in the transaxial SPECT slice demonstrating the largest tumour size, regions of interest (ROI) are drawn around the tumour and adjacent normal liver. For each tumour-normal liver pair, R is calculated as the ratio of the ROI average counts per pixel (C_{tumour} / C_{liver}), and an average of the one to five R values calculated (R_{avg}). The counts per pixel in a SPECT image corrected for attenuation (ignoring the partial volume effect) is directly proportional to activity per unit mass, and thus can be used as a surrogate.

5. Determine the total activity to be administered to the patient, considering that the maximum tolerated radiation absorbed dose in normal liver (D_{liver}) would be 80 Gy (70 Gy in the case of cirrhosis):

$$A_{totalLiver} = D_{liver} \times ([R_{avg} \times M_{tumour}] + M_{liver}) / [\, 49670 \times (1 - [L/100]) \,]$$

6. Determine the total activity to be administered to the patient, considering that the maximum tolerated dose in the lungs (D_{lung}) would be 25 Gy (assuming $M_{lung} = 1000$ g):

$$A_{total\ Lung} = (25 \text{ Gy} \times 1000 \text{ g} / 49670) \times (100 / L)$$

If $A_{total\ Lung}$ is less than $A_{total\ Liver}$, then the total activity to be administered, A_{total}, will be reduced from $A_{total\ Liver}$ to $A_{total\ Lung}$, in order not to exceed 25 Gy in the lungs. In that case, the estimated dose in normal liver is calculated as:

$$D_{liver} = A_{total\ Lung} \times [\ 49670 \times (1 - [L/100])\ ] / ([R_{avg} \times M_{tumour}] + M_{liver})$$

7. The expected dose (in Gy) in the tumour mass may be estimated from the total activity to be administered:

$$D_{tumour} = 49670 \times A_{tumour} / M_{tumour}$$

where $A_{tumour} = A_{total} \times (1 - [L/100]) / (1 + [M_{liver} / (R_{avg} \times M_{tumour})])$

APPENDIX 6 Example SIR-Spheres Radiation Absorbed Dose/Activity Calculation

Suppose a patient has a single (to make it simple) tumour mass in the liver. The total mass of the liver is calculated from contrast enhanced CT scan to be 2100g and of the tumour mass 300g (obtained from estimated volumes using the formula: mass = volume in cc × 1.03 g/cc). The normal liver mass would then be = 2100g – 300g = 1800g.

After drawing ROIs around normal liver and the tumour mass from the transaxial SPECT image that best demonstrates the tumour mass (slice with the largest diameter of tumour mass), suppose the average counts per pixel in the tumour ROI is 3500, and for the liver it is 500.

The tumour-to-normal activity ratio R would be = 3500 / 500 = 7
Also, suppose that lung shunting is 5%.

$$\text{Total activity from Eq. 6} = \frac{25\ \text{Gy} \times 1000 \times (100/5)}{49670} = 10.1\ \text{GBq}$$

$$\text{Total activity from Eq. 8} = \frac{80\ \text{Gy} \times [(7 \times 300) + 1800]}{49670 \times (1 - [5/100])} = \frac{80 \times 3900}{49670 \times 0.95} = 6.6\ \text{GBq}$$

The total activity based on lung dose is greater than that based on normal liver, so 6.6 GBq of activity would deliver an estimated 80 Gy to normal liver. However, since the maximum allowable activity to be administered at any one treatment is capped at 3.0 GBq, this is the final amount of activity that would be administered.

The estimated dose delivered to the lungs from 3.0 GBq using Eq. 6 would be:

$$\text{Lung dose} = \frac{49670 \times 3.0}{1000\text{g} \times (100/5)} = \frac{149010}{20000} = 7.5\ \text{Gy}$$

The total activity in the tumour can be calculated from Eq. 10:

$$\text{Activity in tumour} = \frac{3.0\ \text{GBq} \times (1 - [5/100])}{(1 + [1800\text{g} / (7 \times 300\text{g})])} = \frac{2.85}{1.86} = 1.5\ \text{GBq}$$

The estimated dose delivered to the tumour from Eq. 11 would be:

$$\text{Dose to tumour} = \frac{49670 \times 1.5\ \text{GBq}}{300\text{g}} = 254\ \text{Gy}$$

APPENDIX 7 SIR-Spheres Administered Activity Calculation Tables

The following document provides patient dosing information and is for the Interventional Radiologist and Authorised User performing the SIRT procedure.

Note: These tables replace all previous formulae that have been used in previous trials. Note that the dose to be administered is the maximum total dose for treatment of the whole liver. If treatment is restricted to one lobe of the liver then the dose should be decreased to account for the size of the lobe as a fraction of the total liver size. The exception to this rule is that if the SIR-Spheres are to be delivered by super-selective catheterisation ONLY to the tumour and not to the normal liver parenchyma, then there is no upper limit of dose that can be delivered, provided the radiation dose to the lungs is kept to a tolerable level.

To determine the amount of activity of SIR-Spheres to be implanted the Interventional Radiologist will need to know the following information about the patient to be treated:

1. Lung break-through (%)
2. Body Surface Area (BSA)
3. Tumour Involvement (%)

The appropriate chart, as determined by the lung breakthrough, can then be cross-indexed in order to calculate the activity of SIR-Spheres (GBq) to be implanted.

The 3 charts that follow are all based on different lung breakthrough percentages. These are:

Chart 1: 0 – 10% lung breakthrough
Chart 2: 11 – 15% lung breakthrough
Chart 3: 16 – 20% lung breakthrough

If the lung breakthrough is higher than 20% then the patient is ineligible for SIR-Spheres treatment.

Administered Dose Calculator (GBq)

0-10% Lung Break-Through

Percentage Tumour Involvement

BSA	0-5	6-10	11-15	16-20	21-25	26-30	31-35	36-40	41-45	46-50	51-55	56-60	61-65	66-70	71-75	76-80
1.30-1.35	0.7	0.8	1.0	1.2	1.3	1.4	1.5	1.5	1.5	1.5	1.4	1.3	1.3	1.2	1.1	1.1
1.36-1.40	0.7	0.9	1.0	1.2	1.3	1.4	1.5	1.5	1.5	1.5	1.5	1.4	1.3	1.2	1.2	1.1
1.41-1.45	0.7	0.9	1.1	1.2	1.4	1.5	1.6	1.6	1.6	1.6	1.5	1.4	1.4	1.3	1.2	1.2
1.46-1.50	0.8	0.9	1.1	1.3	1.4	1.5	1.6	1.7	1.7	1.6	1.6	1.5	1.4	1.3	1.3	1.2
1.51-1.55	0.8	1.0	1.1	1.3	1.5	1.6	1.7	1.7	1.7	1.7	1.6	1.5	1.5	1.4	1.3	1.2
1.56-1.60	0.8	1.0	1.2	1.4	1.5	1.6	1.7	1.8	1.8	1.7	1.7	1.6	1.5	1.4	1.3	1.3
1.61-1.65	0.8	1.0	1.2	1.4	1.6	1.7	1.8	1.8	1.8	1.8	1.7	1.6	1.6	1.5	1.4	1.3
1.66-1.70	0.9	1.1	1.3	1.4	1.6	1.7	1.8	1.9	1.9	1.8	1.8	1.7	1.6	1.5	1.4	1.4
1.71-1.75	0.9	1.1	1.3	1.5	1.7	1.8	1.9	1.9	1.9	1.9	1.8	1.7	1.6	1.5	1.5	1.4
1.76-1.80	0.9	1.1	1.3	1.5	1.7	1.8	1.9	2.0	2.0	1.9	1.9	1.8	1.7	1.6	1.5	1.4
1.81-1.85	0.9	1.1	1.4	1.6	1.8	1.9	2.0	2.0	2.0	2.0	1.9	1.8	1.7	1.6	1.5	1.5
1.86-1.90	1.0	1.2	1.4	1.6	1.8	1.9	2.0	2.1	2.1	2.1	2.0	1.9	1.8	1.7	1.6	1.5
1.91-1.95	1.0	1.2	1.4	1.7	1.9	2.0	2.1	2.1	2.1	2.1	2.0	1.9	1.8	1.7	1.6	1.6
1.96-2.00	1.0	1.2	1.5	1.7	1.9	2.1	2.2	2.2	2.2	2.2	2.1	2.0	1.9	1.8	1.7	1.6
2.01-2.05	1.0	1.3	1.5	1.7	1.9	2.1	2.2	2.3	2.3	2.2	2.1	2.0	1.9	1.8	1.7	1.6
2.06-2.10	1.1	1.3	1.6	1.8	2.0	2.2	2.3	2.3	2.3	2.3	2.2	2.1	2.0	1.9	1.8	1.7
2.11-2.15	1.1	1.3	1.6	1.8	2.0	2.2	2.3	2.4	2.4	2.3	2.2	2.1	2.0	1.9	1.8	1.7
2.16-2.20	1.1	1.4	1.6	1.9	2.1	2.3	2.4	2.4	2.4	2.4	2.3	2.2	2.1	1.9	1.8	1.8
2.21-2.25	1.1	1.4	1.7	1.9	2.1	2.3	2.4	2.5	2.5	2.4	2.3	2.2	2.1	2.0	1.9	1.8
2.26-2.30	1.2	1.4	1.7	2.0	2.2	2.4	2.5	2.5	2.5	2.5	2.4	2.3	2.2	2.0	1.9	1.8
2.31-2.35	1.2	1.5	1.7	2.0	2.2	2.4	2.5	2.6	2.6	2.5	2.5	2.3	2.2	2.1	2.0	1.9
2.36-2.40	1.2	1.5	1.8	2.0	2.3	2.5	2.6	2.6	2.6	2.6	2.5	2.4	2.3	2.1	2.0	1.9
2.41-2.45	1.2	1.5	1.8	2.1	2.3	2.5	2.6	2.7	2.7	2.6	2.6	2.4	2.3	2.2	2.0	1.9
2.46-2.50	1.3	1.5	1.8	2.1	2.4	2.6	2.7	2.8	2.8	2.7	2.6	2.5	2.4	2.2	2.1	2.0

Administered Dose Calculator (GBq)

11-15% Lung Break-Through

Percentage Tumour Involvement

BSA	0-5	6-10	11-15	16-20	21-25	26-30	31-35	36-40	41-45	46-50	51-55	56-60	61-65	66-70	71-75	76-80
1.30-1.35	0.7	0.8	1.0	1.2	1.3	1.4	1.5	1.5	1.5	1.5	1.4	1.3	1.3	1.2	1.1	1.1
1.36-1.40	0.7	0.9	1.0	1.2	1.3	1.4	1.5	1.5	1.5	1.5	1.5	1.4	1.3	1.2	1.2	1.1
1.41-1.45	0.7	0.9	1.1	1.2	1.4	1.5	1.6	1.6	1.6	1.6	1.5	1.4	1.4	1.3	1.2	1.2
1.46-1.50	0.8	0.9	1.1	1.3	1.4	1.5	1.6	1.7	1.7	1.6	1.6	1.5	1.4	1.3	1.3	1.2
1.51-1.55	0.8	1.0	1.1	1.3	1.5	1.6	1.7	1.7	1.7	1.7	1.6	1.5	1.5	1.4	1.3	1.2
1.56-1.60	0.8	1.0	1.2	1.4	1.5	1.6	1.7	1.8	1.8	1.7	1.7	1.6	1.5	1.4	1.3	1.3
1.61-1.65	0.8	1.0	1.2	1.4	1.6	1.7	1.8	1.8	1.8	1.8	1.7	1.6	1.6	1.5	1.4	1.3
1.66-1.70	0.9	1.1	1.3	1.4	1.6	1.7	1.8	1.9	1.9	1.8	1.8	1.7	1.6	1.5	1.4	1.4
1.71-1.75	0.9	1.1	1.3	1.5	1.7	1.8	1.9	1.9	1.9	1.9	1.8	1.7	1.6	1.5	1.5	1.4
1.76-1.80	0.9	1.1	1.3	1.5	1.7	1.8	1.9	2.0	2.0	1.9	1.9	1.8	1.7	1.6	1.5	1.4
1.81-1.85	0.9	1.1	1.4	1.6	1.8	1.9	2.0	2.0	2.0	2.0	1.9	1.8	1.7	1.6	1.5	1.5
1.86-1.90	1.0	1.2	1.4	1.6	1.8	1.9	2.0	2.0	2.0	2.0	2.0	1.9	1.8	1.7	1.6	1.5
1.91-1.95	1.0	1.2	1.4	1.7	1.9	2.0	2.0	2.0	2.0	2.0	2.0	1.9	1.8	1.7	1.6	1.6
1.96-2.00	1.0	1.2	1.5	1.7	1.9	2.0	2.0	2.0	2.0	2.0	2.0	2.0	1.9	1.8	1.7	1.6
2.01-2.05	1.0	1.3	1.5	1.7	1.9	2.0	2.0	2.0	2.0	2.0	2.0	2.0	1.9	1.8	1.7	1.6
2.06-2.10	1.1	1.3	1.6	1.8	2.0	2.0	2.0	2.0	2.0	2.0	2.0	2.0	2.0	1.9	1.8	1.7
2.11-2.15	1.1	1.3	1.6	1.8	2.0	2.0	2.0	2.0	2.0	2.0	2.0	2.0	2.0	1.9	1.8	1.7
2.16-2.20	1.1	1.4	1.6	1.9	2.0	2.0	2.0	2.0	2.0	2.0	2.0	2.0	2.0	1.9	1.8	1.8
2.21-2.25	1.1	1.4	1.7	1.9	2.0	2.0	2.0	2.0	2.0	2.0	2.0	2.0	2.0	2.0	1.9	1.8
2.26-2.30	1.2	1.4	1.7	2.0	2.0	2.0	2.0	2.0	2.0	2.0	2.0	2.0	2.0	2.0	1.9	1.8
2.31-2.35	1.2	1.5	1.7	2.0	2.0	2.0	2.0	2.0	2.0	2.0	2.0	2.0	2.0	2.0	2.0	1.9
2.36-2.40	1.2	1.5	1.8	2.0	2.0	2.0	2.0	2.0	2.0	2.0	2.0	2.0	2.0	2.0	2.0	1.9
2.41-2.45	1.2	1.5	1.8	2.0	2.0	2.0	2.0	2.0	2.0	2.0	2.0	2.0	2.0	2.0	2.0	1.9
2.46-2.50	1.3	1.5	1.8	2.0	2.0	2.0	2.0	2.0	2.0	2.0	2.0	2.0	2.0	2.0	2.0	2.0

Administered Dose Calculator (GBq)

16-20% Lung Break-Through

Percentage Tumour Involvement

BSA	0-5	6-10	11-15	16-20	21-25	26-30	31-35	36-40	41-45	46-50	51-55	56-60	61-65	66-70	71-75	76-80
1.30-1.35	0.7	0.8	1.0	1.2	1.3	1.4	1.5	1.5	1.5	1.5	1.4	1.3	1.3	1.2	1.1	1.1
1.36-1.40	0.7	0.9	1.0	1.2	1.3	1.4	1.5	1.5	1.5	1.5	1.5	1.4	1.3	1.2	1.2	1.1
1.41-1.45	0.7	0.9	1.1	1.2	1.4	1.5	1.5	1.5	1.5	1.5	1.5	1.4	1.4	1.3	1.2	1.2
1.46-1.50	0.8	0.9	1.1	1.3	1.4	1.5	1.5	1.5	1.5	1.5	1.5	1.5	1.4	1.3	1.3	1.2
1.51-1.55	0.8	1.0	1.1	1.3	1.5	1.5	1.5	1.5	1.5	1.5	1.5	1.5	1.5	1.4	1.3	1.2
1.56-1.60	0.8	1.0	1.2	1.4	1.5	1.5	1.5	1.5	1.5	1.5	1.5	1.5	1.5	1.4	1.3	1.3
1.61-1.65	0.8	1.0	1.2	1.4	1.5	1.5	1.5	1.5	1.5	1.5	1.5	1.5	1.5	1.5	1.4	1.3
1.66-1.70	0.9	1.1	1.3	1.4	1.5	1.5	1.5	1.5	1.5	1.5	1.5	1.5	1.5	1.5	1.4	1.4
1.71-1.75	0.9	1.1	1.3	1.5	1.5	1.5	1.5	1.5	1.5	1.5	1.5	1.5	1.5	1.5	1.5	1.4
1.76-1.80	0.9	1.1	1.3	1.5	1.5	1.5	1.5	1.5	1.5	1.5	1.5	1.5	1.5	1.5	1.5	1.4
1.81-1.85	0.9	1.1	1.4	1.5	1.5	1.5	1.5	1.5	1.5	1.5	1.5	1.5	1.5	1.5	1.5	1.5
1.86-1.90	1.0	1.2	1.4	1.5	1.5	1.5	1.5	1.5	1.5	1.5	1.5	1.5	1.5	1.5	1.5	1.5
1.91-1.95	1.0	1.2	1.4	1.5	1.5	1.5	1.5	1.5	1.5	1.5	1.5	1.5	1.5	1.5	1.5	1.5
1.96-2.00	1.0	1.2	1.5	1.5	1.5	1.5	1.5	1.5	1.5	1.5	1.5	1.5	1.5	1.5	1.5	1.5
2.01-2.05	1.0	1.3	1.5	1.5	1.5	1.5	1.5	1.5	1.5	1.5	1.5	1.5	1.5	1.5	1.5	1.5
2.06-2.10	1.1	1.3	1.5	1.5	1.5	1.5	1.5	1.5	1.5	1.5	1.5	1.5	1.5	1.5	1.5	1.5
2.11-2.15	1.1	1.3	1.5	1.5	1.5	1.5	1.5	1.5	1.5	1.5	1.5	1.5	1.5	1.5	1.5	1.5
2.16-2.20	1.1	1.4	1.5	1.5	1.5	1.5	1.5	1.5	1.5	1.5	1.5	1.5	1.5	1.5	1.5	1.5
2.21-2.25	1.1	1.4	1.5	1.5	1.5	1.5	1.5	1.5	1.5	1.5	1.5	1.5	1.5	1.5	1.5	1.5
2.26-2.30	1.2	1.4	1.5	1.5	1.5	1.5	1.5	1.5	1.5	1.5	1.5	1.5	1.5	1.5	1.5	1.5
2.31-2.35	1.2	1.5	1.5	1.5	1.5	1.5	1.5	1.5	1.5	1.5	1.5	1.5	1.5	1.5	1.5	1.5
2.36-2.40	1.2	1.5	1.5	1.5	1.5	1.5	1.5	1.5	1.5	1.5	1.5	1.5	1.5	1.5	1.5	1.5
2.41-2.45	1.2	1.5	1.5	1.5	1.5	1.5	1.5	1.5	1.5	1.5	1.5	1.5	1.5	1.5	1.5	1.5
2.46-2.50	1.3	1.5	1.5	1.5	1.5	1.5	1.5	1.5	1.5	1.5	1.5	1.5	1.5	1.5	1.5	1.5

APPENDIX 8 NCI CTC Version 3 Recommendation for Grading of Acute and Subacute Toxicity

The full 'Common Terminology Criteria for Adverse Events v 3.0' can be obtained at the following website: http://ctep.cancer.gov/forms/CTCAEv3.pdf

A brief summary of the key metabolic/laboratory values is listed for easy reference in the table below.

Adverse Event	Grade 1	Grade 2	Grade 3	Grade 4
Albumin	30 – LLN g/L	20 – 30 g/L	< 20 g/L	-
ALP	>ULN – 2.5 x ULN	2.5 – 5.0 x ULN	5.0 – 20.0 x ULN	> 20 x ULN
ALT	>ULN – 2.5 x ULN	2.5 – 5.0 x ULN	5.0 – 20.0 x ULN	> 20 x ULN
AST	>ULN – 2.5 x ULN	2.5 – 5.0 x ULN	5.0 – 20.0 x ULN	> 20 x ULN
Bilirubin	>ULN – 1.5 x ULN	1.5 – 3.0 x ULN	3.0 – 10.0 x ULN	> 10.0 x ULN
Creatinine	>ULN – 1.5 x ULN	1.5 – 3.0 x ULN	3.0 – 6.0 x ULN	> 6.0 x ULN
Haemoglobin	100 – LLN g/L	80 – 100 g/L	65 – 80 g/L	< 65 g/L
Leucocytes	3.0 – LLN x 10^9/L	2.0 – 3.0 x 10^9/L	1.0 – 2.0 x 10^9/L	< 1.0 x 10^9/L
Neutrophils	1.5 – LLN x 10^9/L	1.0 – 1.5 x 10^9/L	0.5 – 1.0 x 10^9/L	< 0.5 x 10^9/L
Platelets	75 – LLN x 10^9/L	50 – 75 x 10^9/L	25 – 50 x 10^9/L	< 25 x 10^9/L

APPENDIX 9 EQ-5D Quality of Life Questionnaire

The following four pages contain the Quality of Life questionnaire that is to be used by study participants during the course of the trial. The EQ-5D questionnaire is a tool that has been designed for rapid use in the clinical setting.

EQ-5D is designed for self-completion by respondents and is ideally suited for use in clinics and face-to-face interviews. It is cognitively simple, taking only a few minutes to complete. Instructions to respondents are included in the questionnaire. At the first visit the patient will be required to complete all three pages. At every visit beyond that the participants will only need to complete the first few pages.

The EQ-5D is used under license from the EuroQol group. The sample version shown here is the United States English version. Sirtex has purchased the rights to use this document in all participating countries.

EQ - 5D

Health Questionnaire

English version for the US

© EuroQoL Group 1997

By placing a checkmark in one box in each group below, please indicate which statements best describe your own health state today.

Mobility

I have no problems in walking about ❑
I have some problems in walking about ❑
I am confined to bed ❑

Self-Care

I have no problems with self-care ❑
I have some problems washing or dressing myself ( )
I am unable to wash or dress myself ( )

Usual Activities (e.g. work, study, housework, family or leisure activities)

I have no problems with performing my usual activities ( )
I have some problems with performing my usual activities ( )
I am unable to perform my usual activities ( )

Pain/Discomfort

I have no pain or discomfort ( )
I have moderate pain or discomfort ( )
I have extreme pain or discomfort ( )

Anxiety/Depression

I am not anxious or depressed ( )
I am moderately anxious or depressed ( )
I am extremely anxious or depressed ( )

To help people say how good or bad a health state is, we have drawn a scale (rather like a thermometer) on which the best state you can imagine is marked 100 and the worst state you can imagine is marked 0

We would like you to indicate on this scale how good or bad your own health is today, in your opinion. Please do this by drawing a line from the box below to whichever point on the scale indicates how good or bad your health state is today.

Your own health state today

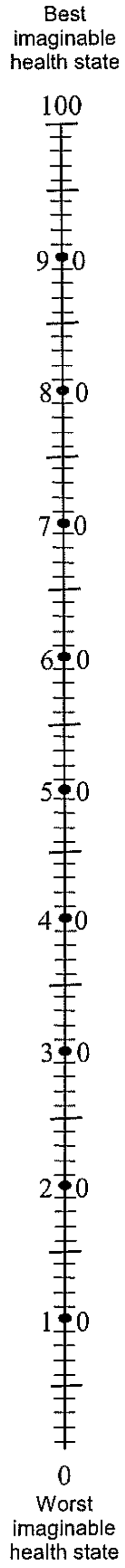

Because all replies are anonymous, it will help us to understand your answers better if we have a little background data from everyone, as covered in the following questions.

1. Have you experienced serious illness?

	Yes	No
in you yourself	☐	☐
in your family	☐	☐
in caring for others	( )	( )

2. What is your age in years?...

3. Are you:

Male	Female
☐	☐

4. Are you:

a current smoker	( )
an ex-smoker	( )
a never smoker	( )

5. Do you now, or did you ever, work in health or social services?

Yes	No
( )	( )

If so, in what capacity? ....

6. Which of the following best describes your main activity?

employed (including self employment)	☐
retired	☐
keeping house	☐
student	☐
seeking work	☐
other (please specify)	☐............................

7. What is the highest level of education you have completed?

some high school or less	☐
high school graduate or GED	☐
vocational college or some college	☐
college degree	☐
professional or graduate degree	☐

8. If you know your zip code, please write it here ....

APPENDIX 10 World Medical Association Declaration of Helsinki

Recommendations Guiding Physicians in Biomedical Research Involving Human Subjects Adopted by the 18th World Medical Assembly, Helsinki, Finland, 1964, and amended by the 29th World Medical Assembly, Tokyo, Japan, 1975; 35th World Medical Assembly, Venice, Italy, 1983; 41st World Medical Assembly, Hong Kong, 1989; and the 48th General Assembly, Somerset West, Republic of South Africa, 1996.

Introduction

It is the mission of the physician to safeguard the health of the people. His or her knowledge and conscience are dedicated to the fulfilment of this mission.

The Declaration of Geneva of the World Medical Association binds the physician with the words, "The health of my patient will be my first consideration," and the International Code of Medical Ethics declares that, "A physician shall act only in the patient's interest when providing medical care which might have the effect of weakening the physical and mental condition of the patient."

The purpose of biomedical research involving human subjects must be to improve diagnostic, therapeutic and prophylactic procedures and the understanding of the aetiology and pathogenesis of disease.
In current medical practice most diagnostic, therapeutic or prophylactic procedures involve hazards. This applies especially to biomedical research

Medical progress is based on research which ultimately must rest in part on experimentation involving human subjects.
In the field of biomedical research a fundamental distinction must be recognised between medical research in which the aim is essentially diagnostic or therapeutic for a patient, and medical research, the essential object of which is purely scientific and without implying direct diagnostic or therapeutic value to the person subjected to the research.
Special caution must be exercised in the conduct of research which may affect the environment, and the welfare of animals used for research must be respected.

Because it is essential that the results of laboratory experiments be applied to human beings to further scientific knowledge and to help suffering humanity, the World Medical Association has prepared the following recommendations as a guide to every physician in biomedical research involving human subjects. They should be kept under review in the future. It must be stressed that the standards as drafted are only a guide to physicians all over the world. Physicians are not relieved from criminal, civil and ethical responsibilities under the laws of their own countries.

I. BASIC PRINCIPLES

Biomedical research involving human subjects must conform to generally accepted scientific principles and should be based on adequately performed laboratory and animal experimentation and on a thorough knowledge of the scientific literature.

The design and performance of each experimental procedure involving human subjects should be clearly formulated in an experimental protocol which should be transmitted for consideration, comment and guidance to a specially appointed committee independent of the investigator provided that this independent committee is in conformity with the laws and regulations of the country in which the research experiment is performed.

Biomedical research involving human subjects should be conducted only by scientifically qualified persons and under the supervision of a clinically competent medical person. The responsibility for the human subject must always rest with a medically qualified person and never rest on the subject of the research, even though the subject has given his or her consent.

Biomedical research involving human subjects cannot legitimately be carried out unless the importance of the objective is in proportion to the inherent risk to the subject.

Every biomedical research project involving human subjects should be preceded by careful assessment of predictable risks in comparison with foreseeable benefits to the subject or to others. Concern for the interests of the subject must always prevail over the interests of science and society.

The right of the research subject to safeguard his or her integrity must always be respected. Every precaution should be taken to respect the privacy of the subject and to minimize the impact of the study on the subject's physical and mental integrity and on the personality of the subject.

Physicians should abstain from engaging in research projects involving human subjects unless they are satisfied that the hazards involved are believed to be predictable. Physicians should cease any investigation if the hazards are found to outweigh the potential benefits.

In publication of the results of his or her research, the physician is obliged to preserve the accuracy of the results. Reports of experimentation not in accordance with the principles laid down in this Declaration should not be accepted for publication.

In any research on human beings, each potential subject must be adequately informed of the aims, methods, anticipated benefits and potential hazards of the study and the discomfort it may entail. He or she should be informed that he or she is at liberty to abstain from participation in the study and that he or she is free to withdraw his or her consent to participation at any time. The physician should then obtain the subject's freely given informed consent, preferably in writing.

When obtaining informed consent for the research project the physician should be

particularly cautious if the subject is in a dependent relationship to him or her or may consent under duress. In that case the informed consent should be obtained by a physician who is not engaged in the investigation and who is completely independent of this official relationship.
In case of legal incompetence, informed consent should be obtained from the legal guardian in accordance with national legislation. Where physical or mental incapacity makes it impossible to obtain informed consent, or when the subject is a minor, permission from the responsible relative replaces that of the subject in accordance with national legislation. Whenever the minor child is in fact able to give a consent, the minor's consent must be obtained in addition to the consent of the minor's legal guardian.

The research protocol should always contain a statement of the ethical considerations involved and should indicate that the principles enunciated in the present Declaration are complied with.

II. MEDICAL RESEARCH COMBINED WITH PROFESSIONAL CARE (Clinical Research)

In the treatment of the sick person, the physician must be free to use a new diagnostic and therapeutic measure, if in his or her judgment it offers hope of saving life, re-establishing health or alleviating suffering.

The potential benefits, hazards and discomfort of a new method should be weighed against the advantages of the best current diagnostic and therapeutic methods.

In any medical study, every patient—including those of a control group, if any—should be assured of the best proven diagnostic and therapeutic method. This does not exclude the use of inert placebo in studies where no proven diagnostic or therapeutic method exists.

The refusal of the patient to participate in a study must never interfere with the physician-patient relationship.

If the physician considers it essential not to obtain informed consent, the specific reasons for this proposal should be stated in the experimental protocol for transmission to the independent committee (I,2).

The physician can combine medical research with professional care, the objective being the acquisition of new medical knowledge, only to the extent that medical research is justified by its potential diagnostic or therapeutic value for the patient.

III. NON-THERAPEUTIC BIOMEDICAL RESEARCH INVOLVING HUMAN SUBJECTS (Non-Clinical Biomedical Research)

In the purely scientific application of medical research carried out on a human being, it is the duty of the physician to remain the protector of the life and health of that person on whom biomedical research is being carried out.

The subjects should be volunteers—either healthy persons or patients for whom the experimental design is not related to the patient's illness.

The investigator or the investigating team should discontinue the research if in his/her or their judgment it may, if continued, be harmful to the individual.

In research on man, the interest of science and society should never take precedence over considerations related to the well-being of the subject.

Asia-Pacific
Hepatocellular Carcinoma Trials Group

PATIENT CONSENT FORM

PHASE I/II STUDY OF SIR-SPHERES® PLUS SORAFENIB (CHEMO-RADIOTHERAPY) AS FIRST LINE TREATMENT IN PATIENTS WITH NON-RESECTABLE PRIMARY HEPATOCELLULAR CARCINOMA

Patient Name and Identification Number: ______________________________

Hospital/Trial Centre: ______________________________

Clinician's name and Signature: ______________________________

I have received an explanation of the trial in ____________ (language / dialect) and have read the information sheet / have had the contents of the information sheet explained to me (delete accordingly) and understand:

Interpreter's name and signature (if relevant): ______________________________

- the nature of the study and what it involves as outlined in the attached patient information sheet
- that if I decide not to participate my future treatment will not be affected in any way
- that I may withdraw from the study at any time.
- that consenting to participate in this trial does not involve any monetary payment made to me for that consent

Sections of my medical records relating to my participation in the study may on occasion, also be viewed by staff of the Clinical Trials and Epidemiology Research Unit who are directly involved with the study or the National Regulatory bodies. All personal details will be treated as strictly confidential. I give my permission for these individuals to have access to my records.

I agree to take part in the study.

Patient Signature ______________________________ Date ____________

I have been present while the study has been explained to the patient and have witnessed his consent to take part.
Name & Signature ______________________________ Date ____________

____

Asia-Pacific
Hepatocellular Carcinoma Trials Group

PATIENT INFORMATION SHEET

PHASE I/II STUDY OF SIR-SPHERES® PLUS SORAFENIB (CHEMO-RADIOTHERAPY) AS FIRST LINE TREATMENT IN PATIENTS WITH NON-RESECTABLE PRIMARY HEPATOCELLULAR CARCINOMA

Introduction

You are invited to take part in a research study on the treatment of advanced liver cancer. This is a multi-national co-operative research effort carried out in countries in the Asia-Pacific region under the auspices of the Asia-Pacific Hepatocellular Carcinoma Trials Group and and co-ordinated by Clinical Trials & Epidemiology Research Unit and National Cancer Centre, Singapore.

The following information describes the study and your role as a participant. Please read carefully, ask the doctor any question that you may have and take your time to decide.

This trial has been reviewed and approved by the Ethics Committee of the medical institution where you are being treated.

Purpose of Study

Your doctor has recently diagnosed that you have primary liver cancer (hepatocellular carcinoma or HCC). This is a very common cancer in this part of the world and many patients such as yourself are suffering from. The best form of treatment for primary liver cancer is surgery as this offers the best chance of cure and prolonged survival. Most patients with this cancer (almost 85%) are however unfortunate in that the disease is already at an advanced stage at the time of diagnosis and surgery is no longer possible. Your disease belongs to this category.

For patients with liver cancer that is too advanced to be operated on, various other types of treatment have been evaluated with a view to improving patient survival and quality of life. These types of treatment include the following: -

- Radiofrequency ablation (RFA)
- Selective Internal Radiation Therapy(SIRT)
- Transarterial Chemo-embolisation (TACE)
- Conventional Chemo-therapy

- Molecular pathway targeted therapy

Of the above treatment, only molecular pathway targeted therapy with the drug Sorafenib has been clearly shown to benefit patients in terms of improved survival. Sorafenib is an oral therapy that treats HCC by attacking the molecular pathway involved in the growth and survival of the tumour. In a large clinical trial the results of which were announced in July 2007, treatment with Sorafenib increases survival in patients with inoperable HCC by an average of 3 months. Sorafenib however has not been shown to significantly reduce the size of HCC but slows down the growth of the cancer. There is less conclusive data regarding improvement in survival with the other treatment.

Selective Internal Radiation Therapy (SIRT) with SIR-sphere which carries a form of short range radiation known as beta radiation has been reported to significantly decrease the size of HCC. SIRT introduces the radiation through an interventional radiology procedure via an artery in the groin known as the femoral artery. There has however insufficient data for doctors to know if it significantly improves survival inpatients in inoperable HCC in most patients.

Presently, inoperable liver cancer affects a large number of patients in the Asia-Pacific region. Hence, your doctors involved in this clinical trial are studying if a combination of Sorafenib therapy and SIRT with SIR-sphere will give patients better survival than that reported with either therapy alone. In the fist phases of this research (phase I and II), your doctors will study if this combination therapy is safe for patients and will result in good response in the cancer, before conducting a larger (phase III) trial. You are thus invited to take part in this trial.

Who can be in the study?

You can participate in the study if you fall under the following criteria:

- Have a confirmed diagnosis of hepatocellular carcinoma by criteria set out in the trial.
- ECOG performance status 0-2
- OKUDA stage I and II for hepatocellular carcinoma
- Good renal function
- Could not be treated by surgery

Patient's Responsibility

The study intends to recruit about 30 patients. To be in the study, you must fulfil the eligibility criteria.

If you are eligible and had given your consent to participate, you will receive SIR-sphere therapy followed by Sorafenib therapy. SIR-sphere therapy wil be administered at the Singapore General Hospital. If you are a patient from outside Singapore, you will be sponsored for a trip to Singapore to receive SIR-sphere therapy. Before Sir-sphere therapy you will be asked to undergo a special scan (MAA Scan) to identify if you are suitable for SIR-sphere therapy. While this scan is fairly safe with low risk of side effects, about 15% of patients will be found to be unsuitable for SIR-sphere therapy and will not be eligible for the trial.

Sorafenib therapy will follow SIR-sphere therapy after either 11 or 14 days (this will be decided by the doctors involved in the trial). This oral treatment will be prescribed to you by your own doctors involved in the trial. If you are from outside Singapore, your doctor in the trial-center in your own country will be the one who will prescribe this medicine to you and will look after you for the duration of the trial. You are required to take the study medication for one year (unless your doctors decide that you suffer from significant adverse effects from the drugs and you should not continue). You must also see your trial doctor for regular follow-up at the clinic. During each visit, the doctor will do a physical examination on you and assess your functional status. You will also need to undergo some blood and radiological investigations to show the status of your liver cancer.

During each visit, you have to bring back your study medications even though the bottle may be partially filled or empty. A new supply of study medication will be given to you at each visit. You also have to fill in the quality of life questionnaire at each visit.

Any patient in the trial has the option to stop participation in the trial any time he wishes with no obligations to the doctor treating him. He can continue to be seen and treated by his doctor using alternative treatment.

Possible Side Effects

While the side effects of these 2 therapies drugs are expected to be minimal there is limited data on eth effect of a combination of these therapies.

From the total experience with SIR-Spheres, major complications have included:

- In approximately one-third of patients, administration of SIRT causes immediate short term abdominal pain requiring narcotic analgesia and is typically self-limiting.
- Post-SIRT lethargy and nausea are common symptoms and can last up to two weeks and may require medication.
- Most patients develop a mild-moderate fever that may last for several days following SIRT administration. This fever does not usually require treatment.
- The most common potential serious complications result from either (i) inadvertent administration of SIR-Spheres into the gastrointestinal tract resulting in gastritis/duodenitis or (ii) radiation induced liver disease resulting from a radiation overdose to the normal liver parenchyma. The incidence of gastritis/duodenitis can be reduced by meticulous attention to the administration procedure so as to ensure that there is a minimal chance of SIR-Spheres entering the numerous small arteries supplying the gastrointestinal tract (Salem 2006; Liu 2005). Radiation induced liver disease is largely, but not totally, preventable by using appropriate SIRT doses and making allowances for dose reduction when there is increased risk of causing radiation damage such as in pre-existing liver damage, poor liver reserve or small volume tumour mass in the liver. The reported incidence of gastritis/duodenitis is <10%, while the reported rate of radiation induced liver disease is < 1%.
- Rare complications that have been reported include acute pancreatitis resulting from SIR-Spheres refluxing in the hepatic artery and lodging in the pancreas, and liver abscess from infection of necrotic tumour.
- Previously reported radiation pneumonitis has not been observed where appropriate pre-treatment workup and dose reductions are followed.
- There is some evidence that there is a decrease in leukocyte levels, with a nadir 8 weeks after

implantation. This has been evident in both first line and refractory studies with studies reporting a median number of leukocytes of 3.55 x 10^9/L (NCIC CTC3 grade 1). Leukocyte levels usually recover from this point, with the median value rising to normal levels 4 - 8 weeks after the decline.

The rate of treatment related complications has been shown to run at 2 – 10%, with outcomes related to the skill and experience of the Interventional Radiologist and Authorized User.

Although Sorafenib is generally well tolerated, some serious adverse events have occurred in some patients treated with Sorafenib in clinical trials, including:

- Diarrhea
- Hand-foot syndrome
- Fatigue
- Rashes
- Anorexia
- Hypertension

The hand and foot symptoms may be resolved with discontinuation of Sorafenib. Symptoms can sometimes be successfully treated with non-steroidal anti-inflammatory agents and urea-containing cream

Anticipated expense

The two therapies administered in the trial will be sponsored and the patient will not have to bear the cost of these therapies. The tests done and the number of visits required for the study is normally what every patient have to undergo even though they are not in the study. Such expenses will be borne by you. However you may have to undergo additional investigations such as CT scan during the trial that your doctor may not order if you are not in the trial. These additional investigations will be paid for by you.

Confidentiality

Only the investigators and government regulatory agencies have access to confidential information, which identifies you by name. You will not be identified in any reports or publications resulting from the study.

Contact person

If you have any additional questions during the course of the study about this research or your rights as a research subject, you may address them to ______________________(name) at ________________(Tel /pager no.)

If you experience a research related injury or any other problems, you may contact ________________________(name) at __________________ (tel. / pager no.)

Please sign the consent form after you have the opportunity to ask questions and have received satisfactory answers to all your questions.

Voluntary participation

Your participation in this study is entirely voluntary. If you do not wish to take part in this study, then your own doctor will decide what treatment they feel is best for you. If you take part in this study and later change your mind, you are free to withdraw from the study without any penalty or loss of benefits to your future care.

We thank you for reading this leaflet and considering helping us with this study.

APPENDIX 13 Abbreviations

Acronyms

5-FU	5-fluorouracil
AIMD	Active Implantable Medical Device
ALT	alanine aminotransferase
AST	aspartate aminotransferase
CRA	Clinical Research Associate
CRF	Case Report Form
CT	computed tomography
EQ-5D	EuroQol Quality of Life Instrument
EU	European Union
FBC	Full Blood Count
FDA	United States Food and Drug Administration
FOLFOX	Oxaliplatin + Leucovorin + 5-Fluorouracil systemic chemotherapy
FUDR	Floxuridine
GCP	Good Clinical Practice
GLP	Good Laboratory Practice
HAC	Hepatic Arterial Chemotherapy
HREC	Human research ethics committee
ICF	Informed consent form
ICH	International Conference on Harmonisation
IDSMB	Independent Data and Safety Monitoring Board
IRCP	International Commission on Radiological Protection
IROX	Oxaliplatin 85 and Irinotecan 200
ISO	International Standards Organization
IVRS	Interactive voice response system
LD	Longest diameter
LV	Leucovorin (folinic acid)
MAA	Macro-aggregated albumin
NCIC CTC	National Cancer Institute of Canada Common Toxicity Criteria
OS	Overall survival
PET	Positron Emission Tomography
PFS	Progression free survival
PMA	PreMarket Approval
RECIST	Response Evaluation Criteria In Solid Tumours
SAE	Serious Adverse Event
SIRT	Selective Internal Radiation Therapy
SMC	Study Management Committee
TGA	Therapeutic Goods Administration
WBC	White Blood Count
WHO	World Health Organisation

RECIST outcome abbreviations

CR	Complete Response
PR	Partial Response
SD	Stable Disease

PD	Progressive Disease

Units of measurement

GBq	GigaBequerel
dL	decaliter
hr	hour
kg	kilogram
L	Liter
mg	milligram
mL	millilitre
mSv	millisieverts
μSv	microsieverts
v	volume
